# leBIBI^QBPP^: a set of databases and a webtool for automatic phylogenetic analysis of prokaryotic sequences

**DOI:** 10.1186/s12859-015-0692-z

**Published:** 2015-08-12

**Authors:** Jean-Pierre Flandrois, Guy Perrière, Manolo Gouy

**Affiliations:** Laboratoire de Biométrie et Biologie Evolutive, UMR CNRS 5558, Université Claude Bernard – Lyon 1, 43 bd. du 11 Novembre 1918, Villeurbanne, 69622 France

**Keywords:** Phylogeny, Taxonomic identification, Prokaryotes

## Abstract

**Background:**

Estimating the phylogenetic position of bacterial and archaeal organisms by genetic sequence comparisons is considered as the gold-standard in taxonomy. This is also a way to identify the species of origin of the sequence. The quality of the reference database used in such analyses is crucial: the database must reflect the up-to-date bacterial nomenclature and accurately indicate the species of origin of its sequences.

**Description:**

leBIBI^QBPP^ is a web tool taking as input a series of nucleotide sequences belonging to one of a set of reference markers (*e.g.*, SSU rRNA, *rpoB*, *groEL*2) and automatically retrieving closely related sequences, aligning them, and performing phylogenetic reconstruction using an approximate maximum likelihood approach. The system returns a set of quality parameters and, if possible, a suggested taxonomic assigment for the input sequences. The reference databases are extracted from GenBank and present four degrees of stringency, from the “superstringent” degree (one type strain per species) to the loosely parsed degree (“lax” database). A set of one hundred to more than a thousand sequences may be analyzed at a time. The speed of the process has been optimized through careful hardware selection and database design.

**Conclusion:**

leBIBI^QBPP^ is a powerful tool helping biologists to position bacterial or archaeal sequence commonly used markers in a phylogeny. It is a diagnostic tool for clinical, industrial and environmental microbiology laboratory, as well as an exploratory tool for more specialized laboratories. Its main advantages, relatively to comparable systems are: i) the use of a broad set of databases covering diverse markers with various degrees of stringency; ii) the use of an approximate Maximum Likelihood approach for phylogenetic reconstruction; iii) a speed compatible with on-line usage; and iv) providing fully documented results to help the user in decision making.

**Electronic supplementary material:**

The online version of this article (doi:10.1186/s12859-015-0692-z) contains supplementary material, which is available to authorized users.

## Background

In clinical microbiology laboratories, but also in environmental and industrial microbiology laboratories, microbial identification is done daily to identify pathogens, food-spoilage linked species, water borne bacteria, or environmental prokaryotes. Conventional phenotypic tests used for species differentiation are progressively replaced by MALDI-TOF identification, although this approach is not designed for universal use but optimized for clinical microbiology [1, 2]. Carl Woese’s seminal work [3] has introduced the 16S ribosomal RNA (SSU rRNA) as the foundation of the modern taxonomy and systematics of prokaryotes. Therefore microbial identification rapidly took into account this innovation [4]. Identification of Bacteria and Archaea by comparing their 16S DNA sequences (SSU rDNA) to those of well determined organisms is now of common use as a diagnostic tool for clinical, industrial and environmental microbiology laboratory, as well as an exploratory tool for more specialized laboratories [5–11]. This practice is *de facto* a gold standard that is used as a control when studying a new identification method [12–17].

The 97 % pragmatic threshold of 16S sequence identity percentage defining the separation between bacterial species and/or genera [18, 19] is widely accepted as a determination criterion, but it was initially designed to define new species, not for strain identification. It is prone to variations due to the quality of the reference and of the unknown sequences [19], its length and the method used to compute percentages of identity [20, 21]. The practical use of this species definition was so important that the Clinical and Laboratory Standards Institute has set-up guidelines for the interpretation of 16S sequence identity percentages [22].

The bioinformatical identification process is basically a comparison of a nucleotide sequence to a database generally involving a BLAST search [20, 23] to find the most similar sequences, and returns pairwise alignments and their statistical analysis. BLAST *E*-value, estimate the level of similarity between sequence pairs but not their evolutionary relatedness [24, 25]. As the first BLAST hit is often not the nearest phylogenetic neighbor [26], this may lead to erroneous species assignment [27]. To overcome this problem, the RDP (Ribosomal Database Project) uses a naive Bayesian rRNA classifier and returns the most probable genus given an input rRNA sequence [21]. For a more accurate identification, it uses a sequence match method that finds sequences most similar to a query sequence using a word matching strategy that does not require prior alignment [28]. This approach seems more accurate than BLAST to find evolutionarily closely related sequences.

Taking results of taxonomic identification from molecular sequence data without perspective may be misleading [29], especially if the identification procedure is based on raw BLAST identity ratios computed from partial sequence alignments [20, 25, 26, 30]. As for other organisms, the delineation of prokaryotic species is phylogenetically-based [31, 32]. A consequence of the fact that “species is the only taxonomic unit that can be defined in phylogenetic terms” [18] is that “additional strains could be affiliated to the species on the basis of partial sequences or a complete gene sequence of one gene of the gene set” [31]. Phylogeny is used to confirm the 97 % similarity rule of thumb, to correct it in the case of very closely related species, or to resolve marginal situations [27]. Phylogeny is also of a great help for taxonomic identification with non SSU rDNA genes [33–38], even if no similarity thresholds were set for those markers. This use of phylogeny was stimulated by the possibility to search for similar sequences and to easily compute a phylogenetic tree using either pairwise [39] or multiple sequence alignments. For that purpose, it is possible to use on-line tools [20, 28, 40–42], locally installed user-friendly programs [43, 44] or more specialized programs like ARB [45]. Note that a commercial solution including the construction of a phylogenetic tree is also available [46, 47].

Applications of taxonomic identification tools are diverse, and their number of citations betrays their every-day use in microbiology laboratories. SSU rDNA is of very common use to identify non-cultivated prokaryotes from various sources and has been validated for environmental or clinical specimens [6, 8, 48–52]. LSU rDNA (or 23S) is less frequently used, mainly because of amplification difficulties and of the resulting lack of reference sequences in the databases [53, 54]. Ribosomal DNA is not the only target gene used for identification purposes, and a wide variety of housekeeping genes have been explored for bacterial identification [55]. The lack of general primers and universal database limits the use of such markers to a genus or a group of species, with the exception of *rpoB* [56]. The use of Multi-Locus Sequence Analysis (MLSA) is promoted to get a more precise identification [57–59] but is not practical in diagnostic (clinical, industrial or environmental) laboratory conditions [60–63]. The time and cost for a complete genome sequencing and the lack of available general databases limit the use of complete genome based MLSA even if bioinformatics tools are now available [64, 65].

Improvement of the efficiency of systems for the identification of prokaryotic organisms requires attention to the quality of the sequences in the reference database, the exact labelling of their species of origin, and a rigorous use of the bacterial nomenclature [20, 41, 42]. More than ten years ago, we developed BIBI, a BioInformatics Bacterial Identification tool [42]. This tool combines a BLAST search with the alignment of resulting similar sequences, and proposes an identification of the species of origin of the input sequence through phylogenetic reconstruction. The reference databases used by BIBI contain tags for sequences of Type strains, and this improves the accuracy of sequence identification by this system. An SSU-rDNA database as well as a database of various housekeeping gene sequences were developed and used by BIBI’s identification pipeline. Since its introduction, the system has evolved to a more sophisticated version called leBIBI, and it has become widely used for identification of Bacteria and Archaea, with about 150,000 identifications annually. The website is also mentioned as the source of bacterial identification in more than 100 articles dealing with the identification of newly described or rarely encountered pathogens in humans [66–69], animals [70–75], or environmental microbiology [76, 77]. It has also been analyzed in reviews of the sequence based identification approach [5, 55, 78–82]. The main difference between BIBI and other alignment-based sequence identification tools is the fact that it promotes a phylogenetic approach. It gives hints for a correct interpretation of its results, and points-out conflicting factors to the microbiologist. The user can thus take a reasoned decision on his/her own. Although BIBI is an assistance to identification and not an automated identification system, it has been compared with other systems that claim to identify down to the species level [83].

Because the workflow for database construction, the program pipelines and the post-treatment scripts used by BIBI were extensively changed since the publication of the original paper, we present here a completely new version of this webware called leBIBI^QBPP^. It is an automated system to produce the phylogenetic analysis of the most closely related sequences in the reference database around a query sequence, using a approximate Maximum Likelihood (ML) approach. It delivers also useful quantitative information to deduce the phylogenetic position of the query sequence in a reference phylogeny.

## Construction and contents

Several databases devoted to various markers are integrated in leBIBI^QBPP^. The largest one is for SSU rDNA. Others are smaller databases of general interest (*rpoB*) and databases that are relevant for a restricted spectrum of bacteria or for niche applications (*e.g., sodA*, *groEL*2). Note that other databases devoted to specific applications or research projects are also available upon request.

### Databases typology

The SSU rDNA databases have five “flavors”. (i) The “lax” database contains all bacterial and archaeal SSU rDNA sequences of GenBank except those for which no taxonomical information more specific than Bacteria or Archaea is reported. It is very comprehensive but contains a large amount of not fully identified sequences. The coverage of genovars is maximum in the “lax” database. (ii) The “stringent” database contains sequences that are identified at the species level with a valid name according to the bacterial nomenclature. It also contains sequences of type strains of newly described bacteria or archaea, an indication that their names are under consideration for eventual validation. These two databases contain a lot of identical sequences and are rather frequently affected by erroneous species identifications. (iii) The “TS-stringent” database contains only sequences of type strains (TS), so that newly described or non validly published species may be missing. This database is less susceptible to be contaminated by erroneous species identifications. (iv) The “superstringent” database is a subset of the previous one where only one or a very small number of sequences is retained for each species. The sequences are those labelled in the List of Prokaryotic names with Standing in Nomenclature (LPSN) as reference sequence for a given species. Identification errors are almost absent there, but newly described species or non validly published species are mostly absent. (v) Lastly, the “genus-level” database is a subset of the “superstringent” database containing only one sequence for each genus: the sequence of the TS of the genus type-species.

As of december 2014, the five databases contain respectively 1,309,339, 234,263, 21,451, 11,289 and 2291 SSU rRNA sequences.

### Construction of the “lax” database

The GenBank database structured under the ACNUC format [84] is the source of the sequences and their annotations. The sequence and annotations of each gene of interest are extracted in a GenBank format flat file using the RAA_Query communication protocol [85]. For each nucleotide sequence longer than 300 bp, the name of the species and other information about the nature of the strain, like the NCBI TaxId, the strain collection Id or the taxonomic rank are extracted from flat files.

Next, a script extracts relevant information concerning the nomenclature compliance and the TS status of each sequence. Species names are checked against the DSMZ (http://www.dsmz.de) and LPSN databases [86] (http://www.bacterio.net) devoted to prokaryotic nomenclature. In some cases, the TaxId allows to correct species names. If a name is validly published, the strain is marked as nomenclature compliant (tag v for valid). In the opposite case, the name is marked as not compliant to nomenclature (tag ?). A list of GenBank Ids that have an erroneous species name has been constructed by hand since 2007 and extended when the evidence of error is reported. GenBank Ids of each sequence are compared to this list, and the occurrence of the erroneous species assignment is flagged (tag X). Tags corresponding to the Nomenclature information for a given sequence are therefore [v/?/X].

The TS status of each sequence is deduced by comparison of the collection Id of the corresponding strain to a database constructed by using the current DSMZ “Prokaryotic Nomenclature up-to-date” Excel file, the LPSN website and a home-made list locally maintained since 2004. The TS status of the strain may be expressed by a T following the strain Id (for example CIP8828T). If this tag is found, and if the name is grammatically correct (two words, the first being capitalized, or four words, one being “subsp.”) the sequence is considered to be that of a TS. In some cases the T flag is used without a grammatically correct name; this may be the preliminary indication of a new species, the sequence is therefore tagged t to prevent any loss of information. At the end of this process, a list of missing TS is built (type strains present in the above-mentioned TS database but not found at this point), and the StrainInfo database is used to manually correct the sequence TS status. A sequence strain quoted as “reference sequence” in LPSN is tagged TT. Other sequences are tagged N. The tags corresponding to the TS information are therefore [TT/T/t/N]. A Fasta-formatted sequence file is then produced using a descriptor constructed according to this grammar:>Genus_species[_subsp_subspecies] ∼[v/?/X] ∼[TT/T/t/N] ∼GenBankId=NCBI taxonomic levels

Other database flavors are constructed extracting corresponding sequences from the “lax” database by searching for regular expressions with the Unix command grep. For instance, the motif TT is used to collect sequences of the “superstringent” database. For each database, a BLAST database is then constructed. Databases for the other markers, for which only three flavors (“lax”, “stringent”, “TS-stringent”) are considered, are constructed by the same procedure. Table 1 presents the databases flavors and specifications.

**Table 1:** List of the genes included in leBIBI^QBPP^

	Prokaryotes	RNA/Protein	Stringency	Nb of sequences
SSU rDNA lax	Archaea+Bacteria	RNA	All sequences	1,309,339
SSU rDNA stringent	Archaea+Bacteria	RNA	Valid names	234,263
SSU rDNA TS stringent	Archaea+Bacteria	RNA	TS sequences	21,451
SSU rDNA superstringent	Archaea+Bacteria	RNA	1 TS/species	11,289
SSU rDNA genus-level	Archaea+Bacteria	RNA	1 TS/genus	2291
LSU rDNA lax	Archaea+Bacteria	RNA	All sequences	19,357
LSU rDNA stringent	Archaea+Bacteria	RNA	Valid names	9735
LSU rDNA TS-stringent	Archaea+Bacteria	RNA	TS/species	2031
tmRNA lax	Bacteria	RNA	All sequences	1273
tmrNA stringent	Bacteria	RNA	Valid names	1044
rpoB lax	Bacteria	Protein	All sequences	29,101
rpoB stringent	Bacteria	Protein	Valid names	20,062
dnaJ+dnak lax	Bacteria	Protein	All sequences	12,780
dnaJ+dnaK stringent	Bacteria	Protein	Valid names	9606
fusA lax	Bacteria	Protein	All sequences	4009
fusA stringent	Bacteria	Protein	Valid names	3463
groEL lax	Bacteria	Protein	All sequences	24,344
groEL stringent	Bacteria	Protein	Valid names	11,845
groES lax	Bacteria	Protein	All sequences	335
groES stringent	Bacteria	Protein	Valid names	277
glyA lax	Bacteria	Protein	All sequences	3155
glyA stringent	Bacteria	Protein	Valid names	2732
gyrB lax	Bacteria	Protein	All sequences	30,537
gyrB stringent	Bacteria	Protein	Valid names	23,803
recA lax	Bacteria	Protein	All sequences	25,616
recA stringent	Bacteria	Protein	Valid names	16,526
sodA lax	Bacteria	Protein	All sequences	3975
sodA stringent	Bacteria	Protein	Valid names	3736
tuf lax	Bacteria	Protein	All sequences	7930
tuf stringent	Bacteria	Protein	Valid names	6756
groEL2 lax	Actinobacteria	Protein	All sequences	2942
groEL2 stringent	Actinobacteria	Protein	Valid names	2086
groEL2 TS-stringent	Actinobacteria	Protein	TS sequences	521

### Quality control of reference databases

The quality control of the SSU rDNA database is done by a script that searches missing TSs by comparing the DSMZ list of species and the TSs of the database. This is both a proof of the correct extraction of all genera, and an indicator of the exhaustivity of the database. As TSs of non-cultivated species are not defined, one sequence for each medically important, non-cultivated species is manually introduced. The increase of the number of sequences for each prokaryotic phylum between two extractions is also checked, as well as the global increase of all TS sequences. Because the other databases are not exhaustive in term of taxonomic coverage, we only check for them the global increase of the number of sequences between two extractions and the global increase of TS sequences.

## System use

A query sequence, or a set of query sequences, is submitted to the server. The user first selects the relevant database for input sequences. Only two parameters are under user control: the number of closely related sequences to be included in the tree; the alignment mode (speed or accuracy).

### Tree building

The first step towards the construction of a phylogenetic tree including the query sequence consists in a BLAST search [23]. BLASTN is run with the query against the selected database with an expectation value set to *E*≤0.1 and without filtering for low-complexity regions. The requested number of sequences with the highest similarity scores is extracted. This is not done if the number of BLAST hits is <10 because it is the sign that something went wrong.

Selected sequences are multiply aligned by MAFFT [87]. Then, the BMGE program [88] with its default parameters is used to trim sequences in the multiple sequence alignment in order to select blocks of sites that are optimally suited for phylogenetic inference.

FastTree [89] is then used to reconstruct the tree by approximate maximum likelihood. The General Time Reversible (GTR) model is used for phylogenetic reconstruction [90] with the Gamma correction for across sites evolutionary rate variation. FastTree also uses the SH (Shimodaira-Hasegawa) test [91] to quickly estimate the reliability of each split in the tree rooted at the middle of the largest tip-to-tip distance.

### Tree visualization

A Scalable Vector Graphic (SVG) version of the tree is computed by SeaView [44] and is modified by a set of Python scripts to decorate sequence labels (species name and GenBank IDs) with hypertext links to LPSN, a local copy of GenBank, and StrainInfo. The positions in the tree of the three best BLAST hits are shown by colored dots. LeBIBI^QBPP^ also outputs another tree where the species name part of sequence labels is replaced by taxonomic information as follows: the full taxonomic classification string of each sequence (with all taxon levels from Bacteria or Archaea to family, genus and species) is computed, the part of these strings that is shared by all sequences of the tree is removed, and what remains is used to build sequence labels. Application of the “branch width as support” option of SeaView allows to graphically display branch support through branch widths: widest branches correspond to SH≥0.95, while thin ones correspond to SH≤0.80. A version of the tree with numerical support values is also available.

### Tree analysis

The sequence with the smallest patristic distance (that is the sum of the length of the branches connecting two leaves) from the query sequence is displayed. A query sequence at small topological distance of database sequences suggests a high evolutionary relatedness between them. This distance allows to define the proximal cluster, that is, all sequences at a topological distance of two nodes or less from the query.

If the distance between the query sequence and the closest species is under the 75 % percentile of the distribution of intra-species distances, leBIBI^QBPP^ outputs a message stating that the query sequence putatively belongs to this species. Next, if the same distance is under the 75 % percentile of the distribution of genus inter-species distances, the message reports the putative belonging of the query sequence to the genus of the closest species. This information depends of course of which reference database is used as wrongly assigned sequences may destroy its accuracy.

A warning message is issued when the closest sequence in terms of patristic distance does not belong to the proximal cluster. The system also issues a message when the closest sequence is not among the five best BLAST hits to point out the relatively frequent cases where the best BLAST hit does not match the evolutionary closest neighbor. Also, the taxonomic diversity within the tree is estimated by collating genera of the tree leaves. If they are all identical, a warning is returned indicating that no outgroup is available. In such case, it is advisable to repeat the analysis after having increased the number of BLAST hits retained for tree building.

### Quality control of taxonomic assignments

We have tested leBIBI^QBPP^ by using some sets of sequences deposited in NCBI PopSets (http://www.ncbi.nlm.nih.gov/popset/). A PopSet is “a set of DNA sequences that have been collected to analyse the evolutionary relatedness of a population. The population could originate from different members of the same species, or from organisms from different species”. Twenty-two PopSets have been used (the sequences are available from the website). We have also used a set of sequences from our laboratory that are available on the leBIBI^QBPP^ website. For each sequence, we have verified that the relevance of the selection by BLAST, of the most closely related sequences and that the phylogenetic reconstruction was of good quality.

The possible recruitment of sequences that are not closely phylogenetically linked to the query is one of the identified weakness of the BLAST approach [26]. The result of this kind of event is the corruption of the phylogeny by an outlier sequence. The outlier usually appears at the end of a long branch, and, additionally, the long-branch attraction effect may attract unrelated sequences and complexify the interpretation. The tree using taxonomic ranks as sequence labels has been specifically developed to identify such BLAST recruitment errors. When the taxonomic rank of the corrupting sequence does not correspond at all to surrounding ones, a “reverse QBPP” (see *infra*) can be performed to clarify this undesirable situation.

## Utility and discussion

The use of leBIBI^QBPP^ is straightforward because it only requires to paste a sequence (or a set of sequences) in a webpage. It is also possible to use a test sequence, randomly chosen in a predefined set, for demonstration purposes or to verify if the system is operational.

### Data analysis

LeBIBI^QBPP^ results appear in a page containing a summary of all computations. All files that were used or generated by leBIBI^QBPP^ are also accessible.

#### Report

**Fig. 1:**
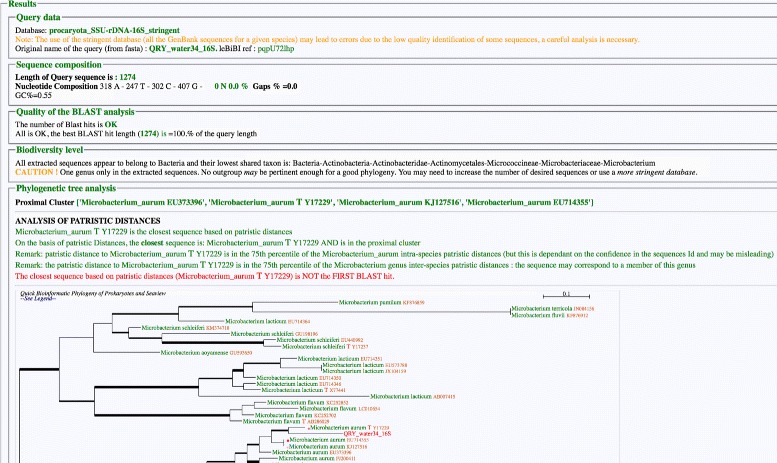
LeBIBI^QBPP^ report summarizes the analysis and gives additional informations that may be useful to interprete the phylogenetic tree

The report (Fig. 1) summarizes the analysis and gives information relevant for the interpretation of the phylogeny and the taxonomic assignment of the query sequence. A summary of the database used is given along with statements about the consequences of the database stringency on the obtained phylogeny.

The nucleotide composition of the query sequence is given. If it contains any undetermined bases, their amount is an indicator of the quality of the sequencing process. Too many undetermined bases have a negative impact on the quality of the phylogeny and this leads to a warning message. The length of the matching section of the first BLAST hit is expected to be close to the length of the query (≥95 %). Even if this does not impair phylogenetic reconstruction, it may indicate a global or local low quality of the query sequence. This indication may point out, for those that continue to use the 97 % identity rule to identify bacterial or archaeal 16S sequences, that this rule is not without shortcomings.

Phylogenetic reconstruction by leBIBI^QBPP^ can be expected to be reliable when the output tree contains sequence clusters from various species and genera around the query sequence. Ideally, an outgroup belonging to another, closely related, genus is required to interpretate the phylogeny. Such an outgroup genus is however not absolutely needed if the genus under consideration contains multiple and phylogenetically distant species.

The goal of the identification of the proximal cluster is to indicate if the query is inside, or close, to a taxonomically homogeneous cluster. Patristic distances between different sequences belonging to the same species or genus help determining whether the query sequence belongs to a given taxon. The closest TS to the query sequence is also shown, as well as its presence in the closest cluster, this to potentially link the query sequence to an existing classification. Even if this is somewhat an approximation, a strain is not expected to be phylogenetically far from the strains of the same species in term of patristic distance; the same is expected for a species within the genus. Therefore, the position of the query sequence in the distribution of intra-species and intra-genus patristic distances is given. The 75 % percentile of these distributions was chosen because of the possible presence of outliers, essentially ill-identified sequences.

The warning that may be output by the comparison between the proximal cluster and the closest sequence indicates a possible phylogenetic reconstruction problem and a careful reading of the tree, taking care of SH support values, may be useful.

#### Phylogenetic trees and alignment

The phylogenetic tree is labelled with sequence names that reflect the compliance of species names with nomenclature and whether they originate from type strains. The expression of the SH support level through branch width enables a direct interpretation of branch robustness. A similar tree with SH support as numerical values is also accessible.

The “Taxo-Tree” has been designed to rapidly identify whether outliers have been erroneously recruited by BLAST, but it may be also useful in the case where nomenclature does not rigorously follow phylogeny, pointing out incoherences.

The sequence alignment is provided as a SVG file to enable a survey of alignment quality.

#### Mitigation of BLAST-induced unexpected phylogenetic tree

The BLAST algorithm searches a database for similar sequences, not for the phylogenetically closest ones. Consequently, a very loosely related sequence gets sometimes recruited. This kind of outlier sequences may lead to difficulties in tree interpretation and is frequently characterized by the apparition of a long branch. A “reverse QBPP” procedure may identify the problem: submitting the outlier sequence, easily accessed through the tree hypertext links, to leBIBI^QBPP^ will lead to a completely different set of selected species and tree.

### Usability

#### Strategy of exploration and databases

The leBIBI^QBPP^ databases and web tools are designed to quickly reconstruct a phylogeny with a SSU rDNA (16S) or a housekeeping protein gene sequence. It also provides elements to help the biologist to interpret the tree and especially to place the query sequence within a known taxonomy rank. As underlined in the databases section above, several different databases are available and QBPP gives a more informative answer if an adapted querying strategy is followed.

The optimal strategy is a trade-off between the advantages of a large number of recruited sequences (it increases the likelihood of having recruited all closely related sequences, and the quality of the coverage of diversity and of the phylogeny), processing speed, and ease in interpretation. The best protocol is to begin with a rather stringent database to maximize phylogenetic and taxonomic diversity. Retaining at least 50 sequences around the query reduces the risk of not recruiting phylogenetically closest strains because they are far in the BLAST hit list. If a broad variety of taxa is obtained (*i.e.*, with external groups, especially genera), it is possible to reduce the number of extracted sequences for better readability, but the user will have to verify that there is no change in the closest clusters. On the contrary, if the tree does not contain enough species diversity, it is necessary to increase the number of extracted sequences or to try a more stringent database. It is always very important to test the “lax” database because some sequences of important uncultured species are present in this collection only.

The “lax” SSU rRNA database is the broadest, so its processing is the slowest. This database contains a lot of sequences that are approximately or wrongly identified or of poor quality and short (albeit >300 bp in length). It should be used to build a phylogeny of the query sequence versus any prokaryote (cultivated, environmental), but often the taxonomy will be difficult to interpret. It is exhaustive, but the phylogenetic signal may be blurred by a swarm of approximately identified sequences, of low quality and possibly redundant. The “lax” SSU rRNA database is therefore essentially exploratory, more suited for research than for routine analyses.

The “stringent” SSU rRNA database is the best when it comes to the quality of the phylogenetic reconstruction because it contains less sequences and has globally better characterized items. It contains sequences of strains that are validly denominated and represent all the biological diversity of species (besides another diversity created by errors in naming or publishing of sequences). Some strains belonging to a species whose members can be phylogenetically very distant (such lack of homogeneity is mostly due to lack of strains or taxonomical studies) cannot be processed without using this “stringent” database. On the contrary it may be impossible to use it in the case of species that are highly represented in GenBank because the phylogeny cannot be computed due to a large number of nearly identical sequences. In such a case the tree is saturated by one species (or genetic variant) and cannot be interpreted. This database is also the only one allowing to compute the distribution of the distances between sequences within one species because it contains often more than one sequence for a given species. Unfortunately it also contains incorrectly identified sequences, introducing uncertainty or errors in the interpretation of the phylogeny.

The “TS-stringent” database (only available for SSU rDNA sequences) is less contaminated by erroneous species names and generally the identification of the sequences is of high quality. This is obtained by a decrease of the variety (mostly one strain per species, the TS being present) that may lead to poor phylogenetic reconstructions in the case of high genomic variations among the species and a TS that is not representative of this diversity. The technical uncertainties in sequencing or possible contaminations or tube-switching explains the observed incoherences of the position of multiple sequences of the same type strain in the phylogenies. Unknown species are also more difficult to position among the already validated species clusters. Uncultured species are mostly missing as their TS are not defined.

The “superstringent” database (also only available for SSU rDNA sequences) is the smallest and fastest to run. The sequences are selected to be representative of a given species and are of high quality. Neither the diversity within a species, nor technically induced biodiversity is represented. As in the similar BLAST database entitled “rRNA typestrains/prokaryotic 16S ribosomal RNA” developed at the NCBI, uncultivated prokaryotes are not present because of the absence of TSs for these species. This is a database giving accurate phylogenies but that may be sometimes incorrect or not representative of the biological reality. The uncultured species are missing as in the “TS-stringent” database.

The “genus-level” database (also only available for SSU rDNA sequences) is the oddest of all. Its sequences are selected to represent all recognized genera. This is mostly useful to build very large phylogenies around a well identified query. Interpretation of the resulting tree may be difficult without studies with less stringent databases.

#### Comparison with other functionally close solutions

LeBIBI^QBPP^ is somewhat similar to other webtools combining the selection of sequences similar to the query (by BLAST or other approaches), and a pipeline that performs multiple alignment, and finally computes a phylogeny. The closest equivalent is the NCBI BLASTN using the “16S ribosomal RNA sequence (Bacteria and Archaea)” database. This database is similar to our “superstringent” database and it is possible to compute a phylogenetic tree using a distance matrix built with BLAST pairwise alignments and either the Fast Minimum Evolution [92] or Neighbor Joining (NJ) [93] algorithms. The alignment between the query sequence and sequences issued from the BLAST search is not a multiple alignment and may only partially cover the query sequence. This is not a true phylogenetic reconstruction unlike done by leBIBI^QBPP^ where a global alignment is computed and the tree is built with the ML approach which outperforms distance methods. The same research on the NCBI site may use the whole GenBank database with the option of suppressing “environmental samples”. This database is then close to our “lax” database but this does not repair the absence of true phylogenetic reconstruction, and in many situations the tree is overcrowded by very similar sequences.

The RDP web site also offers the possibility to load a query sequence, find the closest neighbours in terms of 7-mer sharing percentage (by using Sequence Match) and to build a phylogenetic tree (*via* Tree Builder). The databases provided by this service are known to be of high quality and it is possible to restrict it to cultured bacteria, uncultured or both as well as to TSs, non TSs or both. These selections thus correspond to our “lax” (selection of cultured and non-cultured) or “superstringent” (selection of type-strain sequences only) databases and to other, intermediate choices. The maximum number of matches is limited to 20, but it is possible to select more closest taxa by another procedure (Hierarchy Browser or Sequence Match), and to proceed to their phylogenetic analysis. Alignment is done by the fast, rRNA secondary-structure aware Infernal aligner [94], and the phylogeny is obtained by distance methods such as NJ or Weighbor [95] with bootstrap support computation. At most 50 sequences can be put in the tree. A good knowledge of bacterial taxonomy is required to select the more phylogenetically related neighbours and to select a pertinent outgroup if wanted. Apart from these requirements, the phylogeny obtained is subject to the intrinsic limitations of distance reconstruction methods. This website requires numerous selection and data transfer steps that are not needed in leBIBI^QBPP^ The selection of the recruited sequences that will be used for alignment and phylogeny is not needed in leBIBI^QBPP^, where this is done by choosing the reference database and the number of retained matching sequences.

The last similar tool is provided by the Phylogeny.fr web site [96]. This system allows to perform a BLASTN search and then to compute a phylogeny on a set of homologous sequences. The first main difference with leBIBI^QBPP^ is the fact that the submission of several sequences is not possible. Also, the database choice is limited to GenBank. Consequently, ill-identified sequences and large numbers of nearly identical sequences are often recruited in the resulting phylogenetic trees. In its simplest protocol, this web tool performs multiple sequence alignment computation by Muscle, alignment trimming by GBlocks, phylogenetic reconstruction by PhyML and tree rendering with TreeDyn [97]. Many options allow to customize this process. The main differences between the service provided by Phylogeny.fr and the present tool is that leBIBI^QBPP^ performs all its analyses in one step from the user’s viewpoint, and that its databases are optimized for microbial phylogeny.

### Case Studies

A short SSU rDNA gene fragment of an unknown bacterium was recently sequenced, and studied in our laboratory. Using the “stringent” database with 50 recruited sequences led to an unexpected phylogenetic tree with multiple warnings (Additional file 1). The interpretation was that the tree was unbalanced due to a large number of *Mycobacterium tuberculosis* sequences. *M. lepromatosis* was the closest species in terms of patristic and node distances. As this species has not been validly published yet (this is denoted by the t after the name), the “superstringent” database could not be used. The chosen solution was to increase the number of recruited sequences to 100. The resulting phylogenetic tree was considerably improved (Additional file 2), especially through the recruitment of an outgroup sequence. The *M. leprae* cluster is phylogenetically positioned close to *M. lepromatosis* and *M. haemophilum*, as expected. The query sequence is that of a new species of *Mycobacterium* [98]. This was confirmed by analysis of the *rpoB* sequence obtained from the same bacterial extract and the *rpoB* “stringent” database (Additional file 3).

A set of 44 partial SSU rDNA sequences (1200–1450 bp) have been obtained from bacteria cultivated from filtrated ion-exchanged tap water. The 44 sequences have been processed batch-wise by leBIBI^QBPP^ with the “TS-stringent” database as reference in a five-minute run (Additional file 4). In most cases, the closest sequence to the query belongs to its proximal cluster. Therefore, the taxonomic assignment of 41 strains was highly reliable according to the criteria presented above. In three cases, the query was not clearly inside or close to a cluster. These three sequences require further expertise as they may belong to new taxonomic entities, species or genera.

## Conclusion

LeBIBI^QBPP^ is a unique tool helping biologists to build phylogenies involving prokaryotic species, in order to achieve the taxonomic assignment of sequences of interest. It is unique in the sense that it provides nomenclature-driven specialized databases, as well as a set of efficient programs allowing to reconstruct robust phylogenies, without sacrificing the speed required for an on-line service. First, leBIBI^QBPP^ is different due to the use of a broad range of databases devoted to prokaryotes, and the careful selection of relevant sequences. Also, the emphasis on annotation quality for prokaryotic taxonomy cannot be found elsewhere. Second, multiple alignment trimming, as well as the use of an ML approach improve the quality of reconstructed trees. It could be argued that there are programs that perform better than FastTree for reconstructing phylogenies, but this comes with an important price in terms of processing speed. Lastly, a very important point is the fact that leBIBI^QBPP^ does not impose an automated, fixed taxonomic assignment, but rather a panel of possible choices, open to interpretation.

## Availability and requirements

The databases and identification tools can be accessed at http://umr5558-bibiserv.univ-lyon1.fr/lebibi/lebibi.cgi and any recent web browser can be used.

## Additional files

Additional file 1
**Phylogenetic placement of an undescribed bacterial sequence.** The analysis of a short SSU rDNA gene fragment of an unknown bacterium using the “stringent” database with 50 recruited sequences led to an unexpected phylogenetic tree with multiple warnings. (784 Kb)

Additional file 2
**Improvement of the phylogenetic placement of an undescribed bacterial sequence.** When the short SSU rDNA gene fragment of an unknown bacterium is studied with 100 recruited sequences, the interpretation is greatly improved especially through the recruitment of an outgroup sequence. (781 Kb)

Additional file 3
**Phylogenetic placement of an undescribed bacterial sequence using another gene and database.** The query sequence is suspected to be a new species of Mycobacterium. This was confirmed by the analysis of the rpoB sequence obtained from the same bacterial extract and the rpoB “stringent” database. (773 Kb)

Additional file 4
**Placing bacteria cultivated from tap water in the phylogeny.** A set of 44 partial SSU rDNA sequences have been obtained from bacteria cultivated from filtrated ion-exchanged tap water. The 44 sequences have been processed batch-wise. The html file is the concatenation of the 44 reports. It is stored in the “globalbibiresults.html” in the result directory. (5 529 Kb)
