# Supplementary figures and images for: leBIBIQBPP: a set of databases and a webtool for automatic phylogenetic analysis of prokaryotic sequences
Source: BMC Bioinformatics. 2015 Aug 12;16(1):251. doi: 10.1186/s12859-015-0692-z (PMC4531848; doi:10.1186/s12859-015-0692-z)

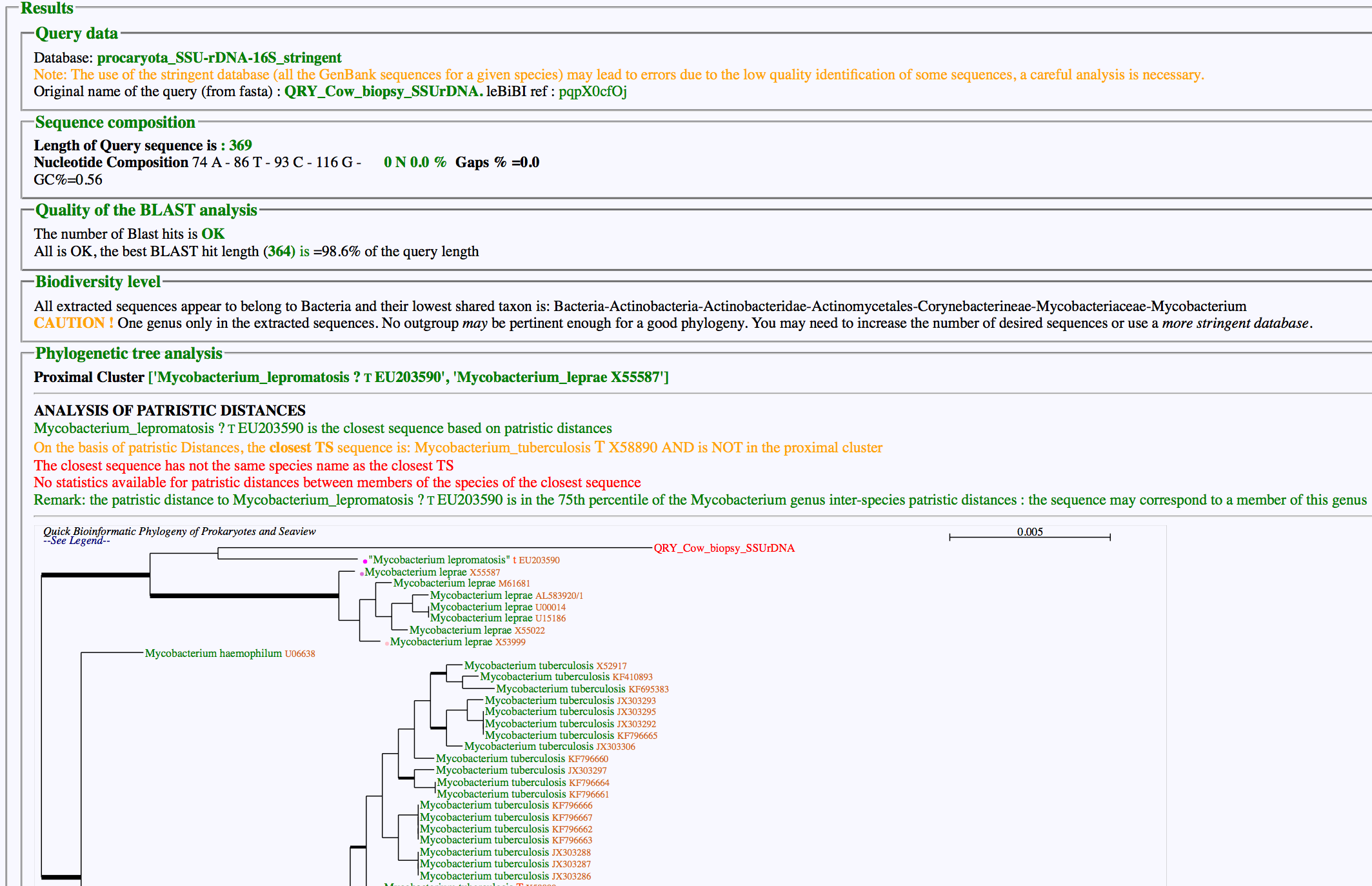

Supplement: Additional file 1 — Phylogenetic placement of an undescribed bacterial sequence. The analysis of a short SSU rDNA gene fragment of an unknown bacterium using the “stringent” database with 50 recruited sequences led to an unexpected phylogenetic tree with multiple warnings. (784 Kb) [file 12859_2015_692_MOESM1_ESM.tiff]

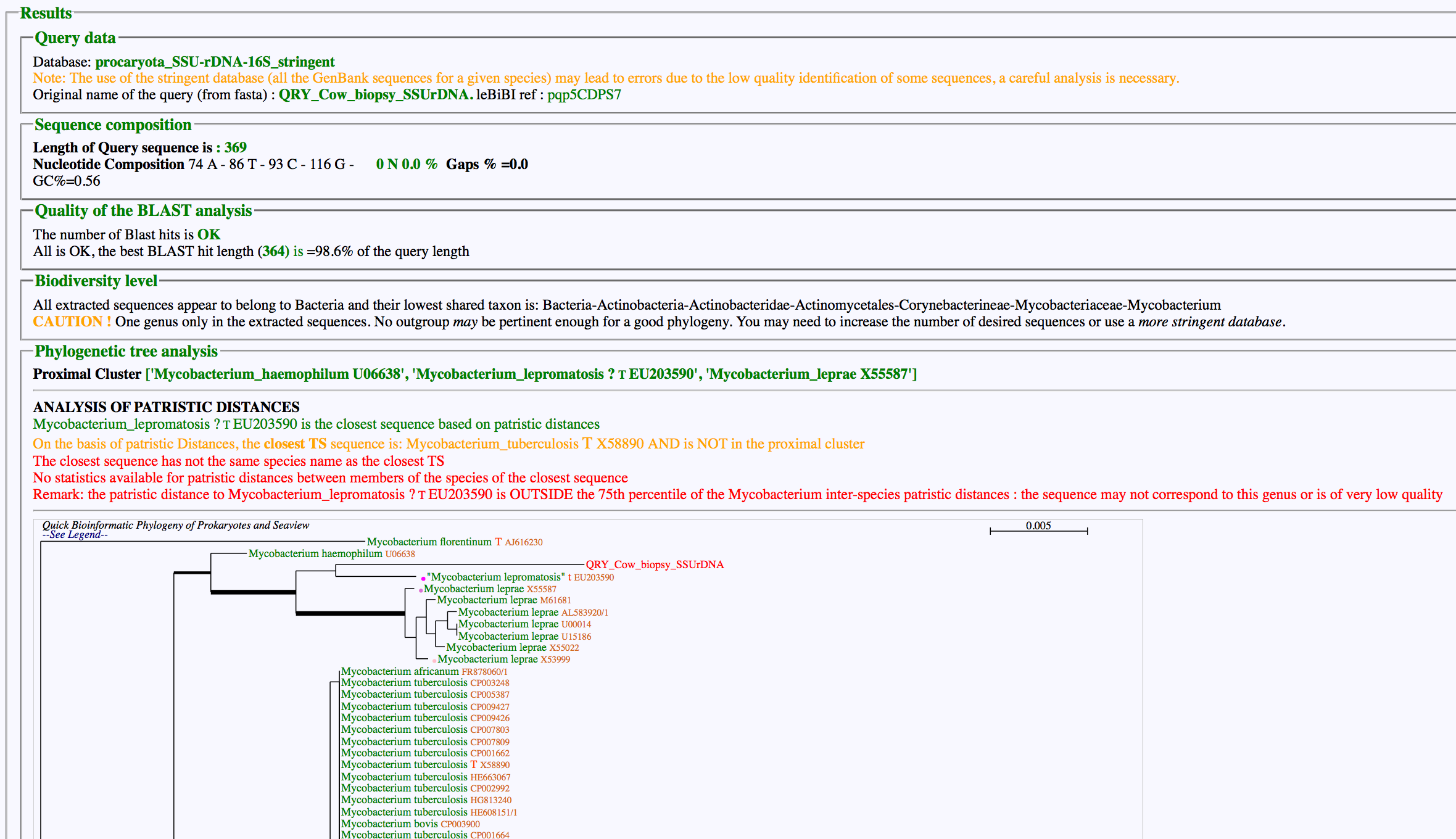

Supplement: Additional file 2 — Improvement of the phylogenetic placement of an undescribed bacterial sequence. When the short SSU rDNA gene fragment of an unknown bacterium is studied with 100 recruited sequences, the interpretation is greatly improved especially through the recruitment of an outgroup sequence. (781 Kb) [file 12859_2015_692_MOESM2_ESM.tiff]

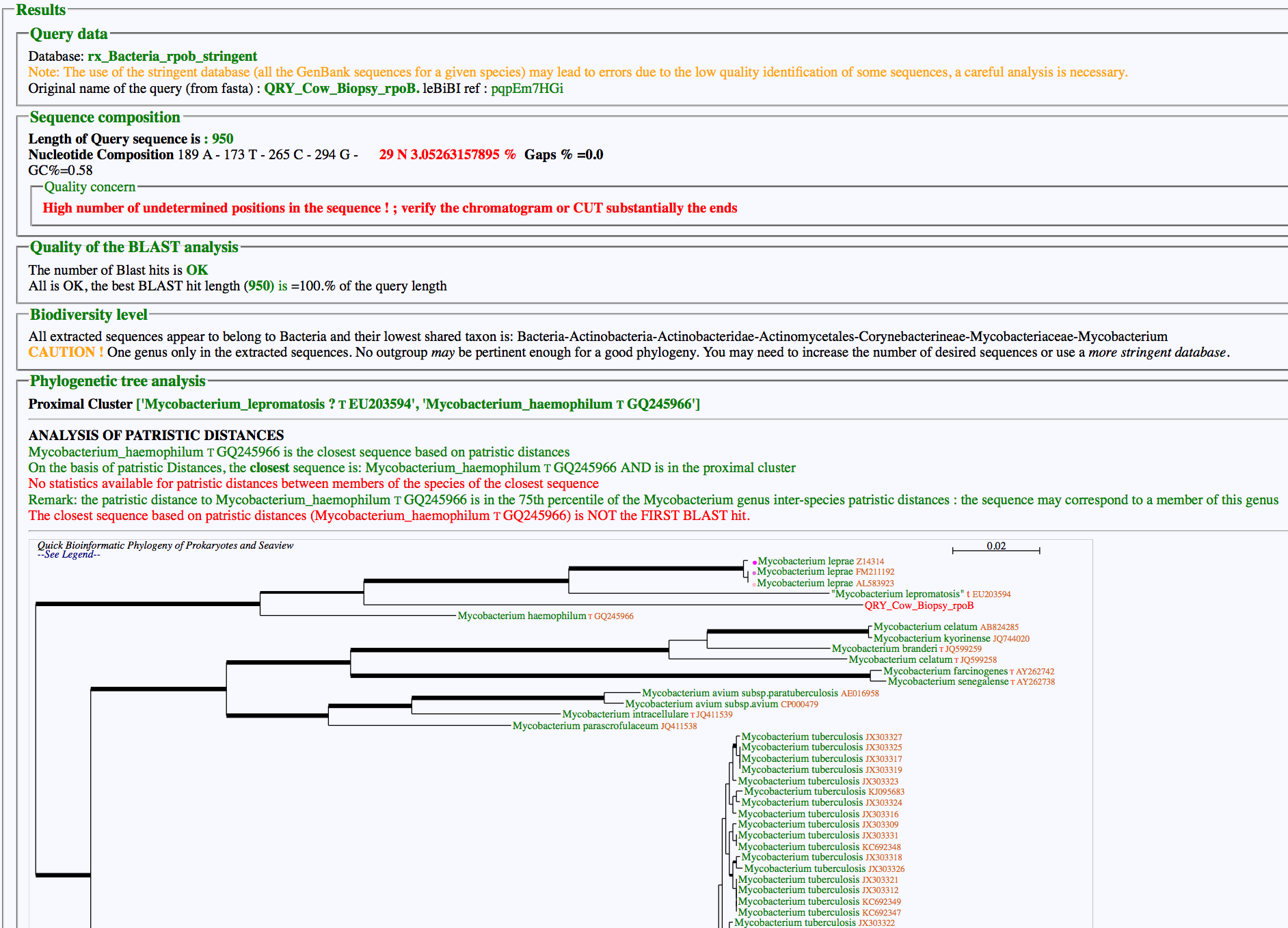

Supplement: Additional file 3 — Phylogenetic placement of an undescribed bacterial sequence using another gene and database. The query sequence is suspected to be a new species of Mycobacterium. This was confirmed by the analysis of the rpoB sequence obtained from the same bacterial extract and the rpoB “stringent” database. (773 Kb) [file 12859_2015_692_MOESM3_ESM.tiff]
