# Supplementary material for: leBIBIQBPP: a set of databases and a webtool for automatic phylogenetic analysis of prokaryotic sequences
Source: BMC Bioinformatics. 2015 Aug 12;16(1):251. doi: 10.1186/s12859-015-0692-z (PMC4531848; doi:10.1186/s12859-015-0692-z)
Supplement: Additional file 4 — Placing bacteria cultivated from tap water in the phylogeny. A set of 44 partial SSU rDNA sequences have been obtained from bacteria cultivated from filtrated ion-exchanged tap water. The 44 sequences have been processed batch-wise. The html file is the concatenation of the 44 reports. It is stored in the “globalbibiresults.html” in the result directory. (5 529 Kb) [file 12859_2015_692_MOESM4_ESM.pdf]

lebibi Quick BioInformatic Phylogeny of Prokaryotes

Summary of the methods and database used during this session

Analysis Extraction of similar sequences for ingroup sequences: Query
There are 44 sequences in your batch
BLAST using the procaryota_SSU-rDNA-16S_TS-stringent database. Trying to extract 50 sequences with expect lower than 0.1
ALIGNMENT using mafft Options : speed oriented
BMGE use for alignment optimization Options :defaults parameters
TREE : phylogenetic tree using fasttree
Evolution model GTR -gamma

RESULTS

Results

Query data

Database: **procaryota_SSU-rDNA-16S_TS-stringent**
Note: The use of the stringent database (all the GenBank sequences for a given species) may lead to errors due to the low quality identification of some sequences, a careful analysis is necessary.
Original name of the query (from fasta) : **QRY_water45_16S.** leBiBI ref : pqpIvwPtL

Sequence composition

Length of Query sequence is : 1279
Nucleotide Composition 307 A - 249 T - 303 C - 420 G - **0 N 0.0 %** **Gaps % =0.0**
GC%=0.56

Quality of the BLAST analysis

The number of Blast hits is **OK**
All is OK, the best BLAST hit length (**1273**) is =99.5% of the query length

Biodiversity level

All extracted sequences appear to belong to Bacteria and their lowest shared taxon is: Bacteria-Actinobacteria-Actinobacteridae-Actinomycetales-Micrococcineae-Microbacteriaceae-Microbacterium
CAUTION ! One genus only in the extracted sequences. No outgroup *may* be pertinent enough for a good phylogeny. You may need to increase the number of desired sequences or use a *more stringent database*.

Phylogenetic tree analysis

Proximal Cluster ['Microbacterium_maritypicum T AM181506']

ANALYSIS OF PATRISTIC DISTANCES
Microbacterium_maritypicum T AM181506 is the closest sequence based on patristic distances
On the basis of patristic Distances, the **closest** sequence is: Microbacterium_maritypicum T AM181506 AND is in the proximal cluster
No statistics available for patristic distances between members of the species of the closest sequence
Remark: the patristic distance to Microbacterium_maritypicum T AM181506 is in the 75th percentile of the Microbacterium genus inter-species patristic distances : the sequence may correspond to a member of this genus

---

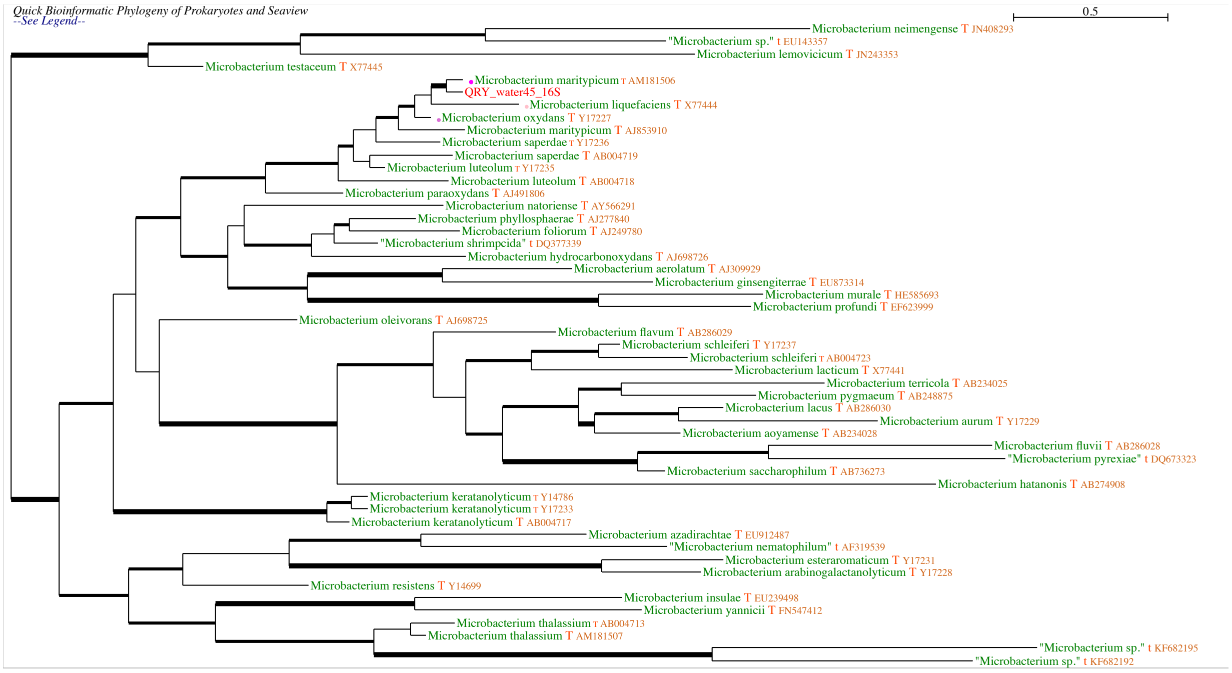

in short | How-to | Cite | Tests sets | Databases | Contact us

lebibi Quick BioInformatic Phylogeny of Prokaryotes

Summary of the methods and database used during this session

Analysis Extraction of similar sequences for ingroup sequences: Query
There are 44 sequences in your batch
BLAST using the procaryota_SSU-rDNA-16S_TS-stringent database. Trying to extract 50 sequences with expect lower than 0.1
ALIGNMENT using mafft Options : speed oriented
BMGE use for alignment optimization Options :defaults parameters
TREE : phylogenetic tree using fasttree
Evolution model GTR -gamma

RESULTS

Results

Query data

Database: **procaryota_SSU-rDNA-16S_TS-stringent**
Note: The use of the stringent database (all the GenBank sequences for a given species) may lead to errors due to the low quality identification of some sequences, a careful analysis is necessary.
Original name of the query (from fasta) : **QRY_water46_16S.** leBiBI ref : pqpIvwPtL

Sequence composition

Length of Query sequence is : 1280
Nucleotide Composition 317 A - 245 T - 305 C - 413 G - **0 N 0.0 %** **Gaps % =0.0**
GC%=0.56

Quality of the BLAST analysis

The number of Blast hits is **OK**
All is OK, the best BLAST hit length (**1280**) is =100.% of the query length

Biodiversity level

All extracted sequences appear to belong to Bacteria and their lowest shared taxon is: Bacteria-Actinobacteria-Actinobacteridae-Actinomycetales-Micrococcineae-Microbacteriaceae-Microbacterium
CAUTION ! One genus only in the extracted sequences. No outgroup *may* be pertinent enough for a good phylogeny. You may need to increase the number of desired sequences or use a *more stringent database*.

Phylogenetic tree analysis

Proximal Cluster ['Microbacterium_lacticum T X77441']

ANALYSIS OF PATRISTIC DISTANCES
Microbacterium_lacticum T X77441 is the closest sequence based on patristic distances
On the basis of patristic Distances, the **closest** sequence is: Microbacterium_lacticum T X77441 AND is in the proximal cluster
No statistics available for patristic distances between members of the species of the closest sequence
Remark: the patristic distance to Microbacterium_lacticum T X77441 is in the 75th percentile of the Microbacterium genus inter-species patristic distances : the sequence may correspond to a member of this genus

---

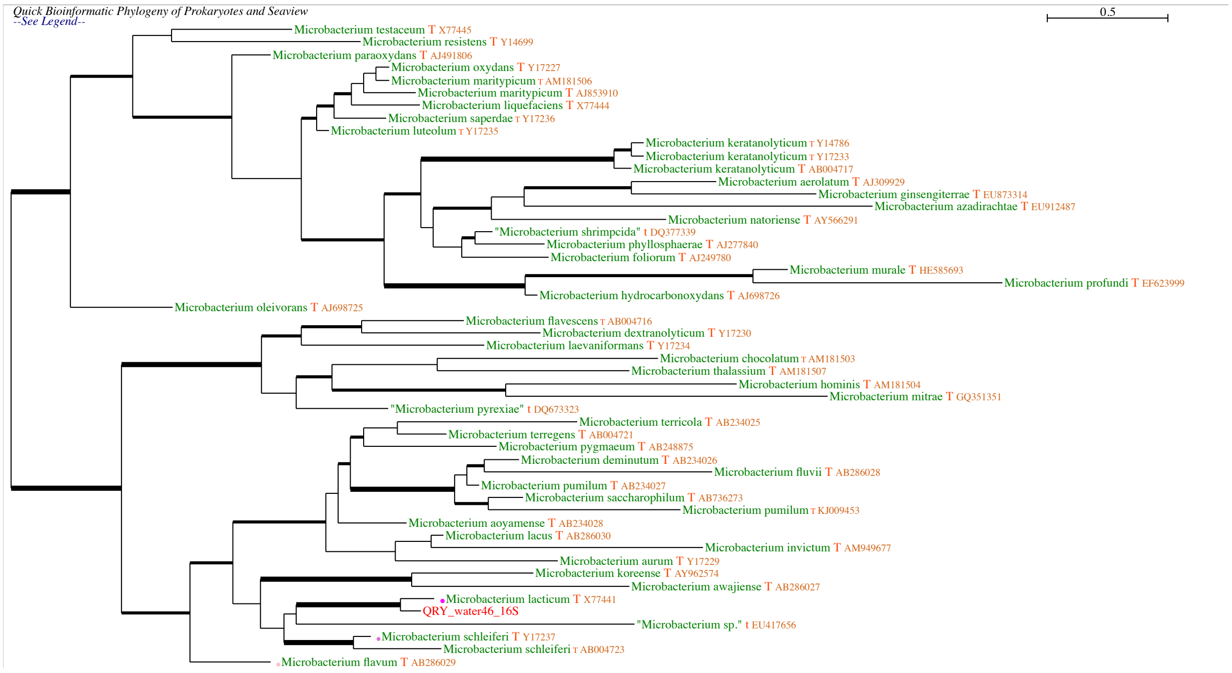

lebibi QBPP Quick Bioinformatic Phylogeny of Prokaryotes | in short | How-to | Cite | Tests sets | Databases | Contact us | LBBE | prabi

lebibi Quick BioInformatic Phylogeny of Prokaryotes

Summary of the methods and database used during this session

Analysis Extraction of similar sequences for ingroup sequences: Query
There are 44 sequences in your batch
BLAST using the procaryota_SSU-rDNA-16S_TS-stringent database. Trying to extract 50 sequences with expect lower than 0.1
ALIGNMENT using mafft Options : speed oriented
BMGE use for alignment optimization Options :defaults parameters
TREE : phylogenetic tree using fasttree
Evolution model GTR -gamma

RESULTS

Results

Query data

Database: **procaryota_SSU-rDNA-16S_TS-stringent**
Note: The use of the stringent database (all the GenBank sequences for a given species) may lead to errors due to the low quality identification of some sequences, a careful analysis is necessary.
Original name of the query (from fasta) : **QRY_water47_16S.** leBiBI ref : pqpIvwPtL

Sequence composition

Length of Query sequence is : 1267
Nucleotide Composition 300 A - 253 T - 298 C - 416 G - **0 N 0.0 % Gaps % =0.0**
GC%=0.56

Quality of the BLAST analysis

The number of Blast hits is **OK**
All is OK, the best BLAST hit length **(1267)** is =100.% of the query length

Biodiversity level

All extracted sequences appear to belong to Bacteria and their lowest shared taxon is: Bacteria-Actinobacteria-Actinobacteridae-Actinomycetales-Micrococcineae-Microbacteriaceae-Microbacterium
CAUTION ! One genus only in the extracted sequences. No outgroup *may* be pertinent enough for a good phylogeny. You may need to increase the number of desired sequences or use a *more stringent database*.

Phylogenetic tree analysis

Proximal Cluster ['Microbacterium_hydrothermale T HM222660', 'Microbacterium_takaoensis ? T AB201047']

ANALYSIS OF PATRISTIC DISTANCES
Microbacterium_takaoensis ? T AB201047 is the closest sequence based on patristic distances
On the basis of patristic Distances, the **closest TS** sequence is: Microbacterium_trichothecenolyticum T Y17240 AND is NOT in the proximal cluster
The closest sequence has not the same species name as the closest TS
No statistics available for patristic distances between members of the species of the closest sequence
Remark: the patristic distance to Microbacterium_takaoensis ? T AB201047 is in the 75th percentile of the Microbacterium genus inter-species patristic distances : the sequence may correspond to a member of this genus

---

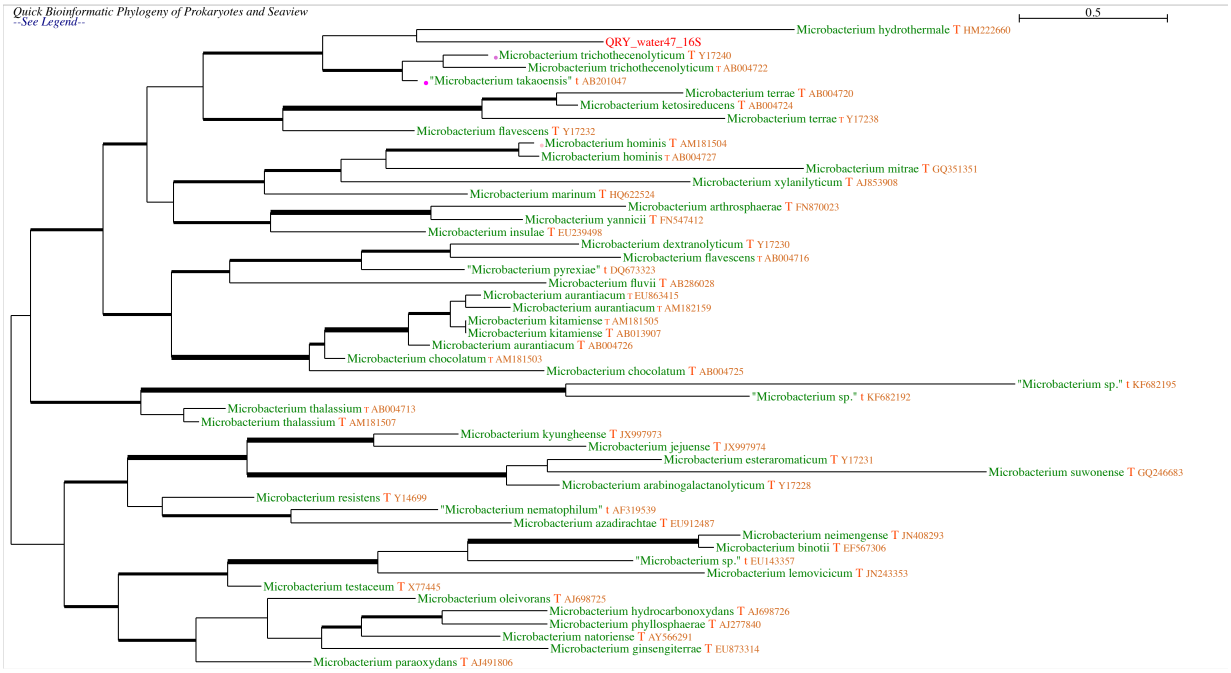

lebibi Quick BioInformatic Phylogeny of Prokaryotes

Summary of the methods and database used during this session

Analysis Extraction of similar sequences for ingroup sequences: Query
There are 44 sequences in your batch
BLAST using the procaryota_SSU-rDNA-16S_TS-stringent database. Trying to extract 50 sequences with expect lower than 0.1
ALIGNMENT using mafft Options : speed oriented
BMGE use for alignment optimization Options :defaults parameters
TREE : phylogenetic tree using fasttree
Evolution model GTR -gamma

RESULTS

Results

Query data

Database: **procaryota_SSU-rDNA-16S_TS-stringent**
Note: The use of the stringent database (all the GenBank sequences for a given species) may lead to errors due to the low quality identification of some sequences, a careful analysis is necessary.
Original name of the query (from fasta) : **QRY_water48_16S.** leBiBI ref : pqpIvwPtL

Sequence composition

Length of Query sequence is : 1233
Nucleotide Composition 292 A - 245 T - 295 C - 401 G - **0 N 0.0 %** **Gaps % =0.0**
GC%=0.56

Quality of the BLAST analysis

The number of Blast hits is **OK**
All is OK, the best BLAST hit length (**1233**) is =100.% of the query length

Biodiversity level

All extracted sequences appear to belong to Bacteria and their lowest shared taxon is: Bacteria-Proteobacteria-Alphaproteobacteria-Rhizobiales-Methylobacteriaceae-Methylobacterium
CAUTION ! One genus only in the extracted sequences. No outgroup *may* be pertinent enough for a good phylogeny. You may need to increase the number of desired sequences or use a *more stringent database*.

Phylogenetic tree analysis

Proximal Cluster ['Methylobacterium_thiocyanatum T AB175646', 'Methylobacterium_thiocyanatum T U58018', 'Methylobacterium_rhodesianum T AB175643', 'Methylobacterium_rhodesianum T AB175642']

ANALYSIS OF PATRISTIC DISTANCES
Methylobacterium_thiocyanatum T AB175646 is the closest sequence based on patristic distances
On the basis of patristic Distances, the **closest** sequence is: Methylobacterium_thiocyanatum T AB175646 AND is in the proximal cluster
No statistics available for patristic distances between members of the species of the closest sequence
Remark: the patristic distance to Methylobacterium_thiocyanatum T AB175646 is in the 75th percentile of the Methylobacterium genus inter-species patristic distances : the sequence may correspond to a member of this genus
The closest sequence based on patristic distances (Methylobacterium_thiocyanatum T AB175646) is NOT the FIRST BLAST hit.

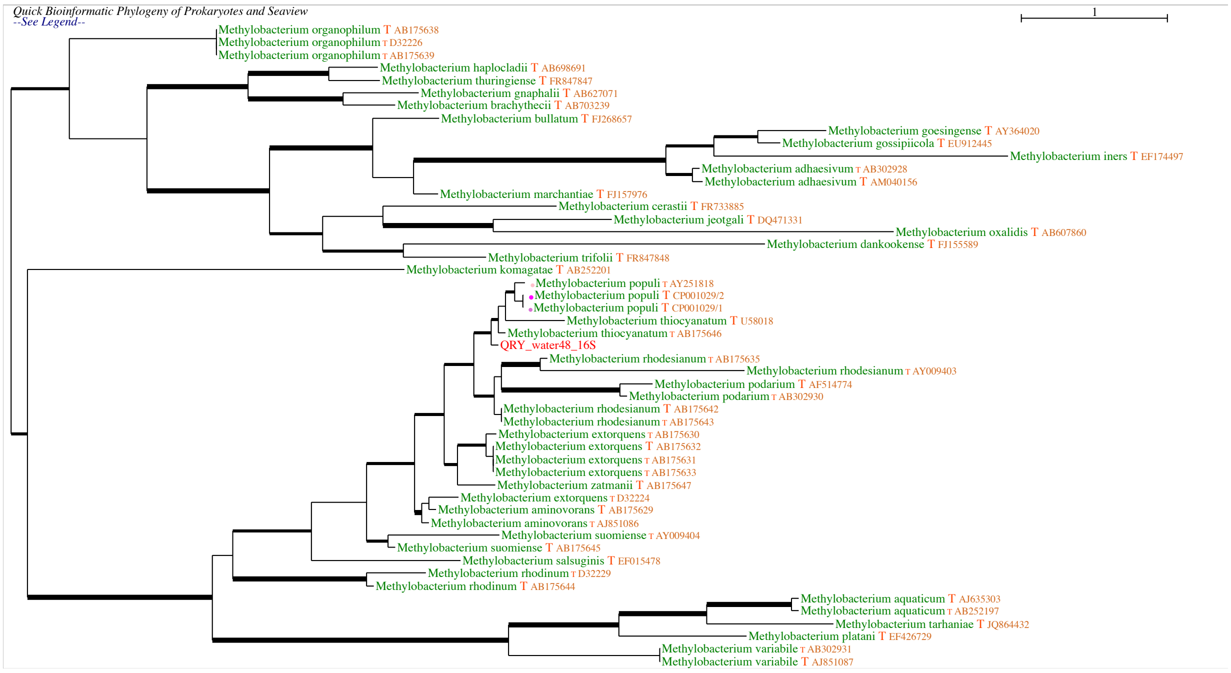

in short | How-to | Cite | Tests sets | Databases | Contact us

lebibi Quick BioInformatic Phylogeny of Prokaryotes

Summary of the methods and database used during this session

Analysis Extraction of similar sequences for ingroup sequences: Query
There are 44 sequences in your batch
BLAST using the procaryota_SSU-rDNA-16S_TS-stringent database. Trying to extract 50 sequences with expect lower than 0.1
ALIGNMENT using mafft Options : speed oriented
BMGE use for alignment optimization Options :defaults parameters
TREE : phylogenetic tree using fasttree
Evolution model GTR -gamma

RESULTS

Results

Query data

Database: **procaryota_SSU-rDNA-16S_TS-stringent**
Note: The use of the stringent database (all the GenBank sequences for a given species) may lead to errors due to the low quality identification of some sequences, a careful analysis is necessary.
Original name of the query (from fasta) : **QRY_water49_16S.** leBiBI ref : pqpIvwPtL

Sequence composition

Length of Query sequence is : 1269
Nucleotide Composition 312 A - 243 T - 305 C - 409 G - **0 N 0.0 %** **Gaps % =0.0**
GC%=0.56

Quality of the BLAST analysis

The number of Blast hits is **OK**
All is OK, the best BLAST hit length (**1267**) is =99.8% of the query length

Biodiversity level

All extracted sequences appear to belong to Bacteria and their lowest shared taxon is: Bacteria-Actinobacteria-Actinobacteridae-Actinomycetales-Micrococcineae-Microbacteriaceae-Microbacterium
CAUTION ! One genus only in the extracted sequences. No outgroup *may* be pertinent enough for a good phylogeny. You may need to increase the number of desired sequences or use a *more stringent database*.

Phylogenetic tree analysis

Proximal Cluster ['Microbacterium_lacticum T X77441']

ANALYSIS OF PATRISTIC DISTANCES
Microbacterium_lacticum T X77441 is the closest sequence based on patristic distances
On the basis of patristic Distances, the **closest** sequence is: Microbacterium_lacticum T X77441 AND is in the proximal cluster
No statistics available for patristic distances between members of the species of the closest sequence
Remark: the patristic distance to Microbacterium_lacticum T X77441 is in the 75th percentile of the Microbacterium genus inter-species patristic distances : the sequence may correspond to a member of this genus

---

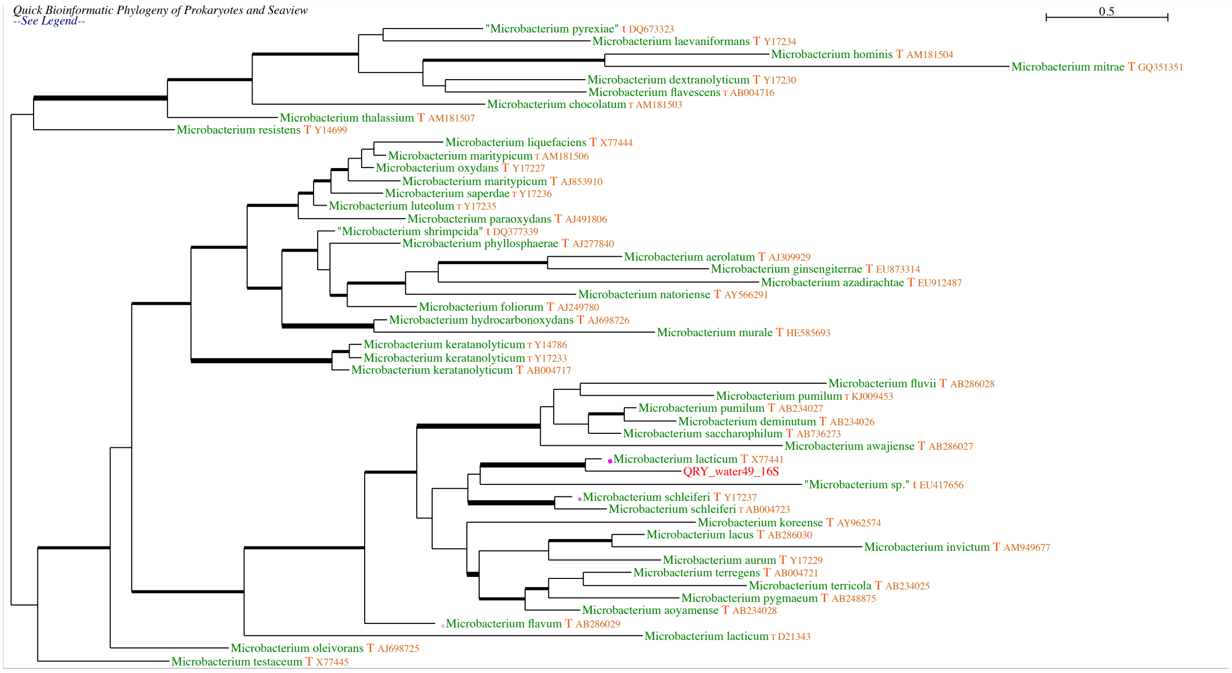

in short | How-to | Cite | Tests sets | Databases | Contact us

lebibi Quick BioInformatic Phylogeny of Prokaryotes

Summary of the methods and database used during this session

Analysis Extraction of similar sequences for ingroup sequences: Query
There are 44 sequences in your batch
BLAST using the procaryota_SSU-rDNA-16S_TS-stringent database. Trying to extract 50 sequences with expect lower than 0.1
ALIGNMENT using mafft Options : speed oriented
BMGE use for alignment optimization Options :defaults parameters
TREE : phylogenetic tree using fasttree
Evolution model GTR -gamma

RESULTS

Results

Query data

Database: **procaryota_SSU-rDNA-16S_TS-stringent**

Note: The use of the stringent database (all the GenBank sequences for a given species) may lead to errors due to the low quality identification of some sequences, a careful analysis is necessary.

Original name of the query (from fasta) : **QRY_water50_16S.** leBiBI ref : pqpIvwPtL

Sequence composition

Length of Query sequence is : 1281
Nucleotide Composition 316 A - 245 T - 306 C - 414 G - **0 N 0.0 % Gaps % =0.0**
GC%=0.56

Quality of the BLAST analysis

The number of Blast hits is **OK**
All is OK, the best BLAST hit length (**1281**) is =100.% of the query length

Biodiversity level

All extracted sequences appear to belong to Bacteria and their lowest shared taxon is: Bacteria-Actinobacteria-Actinobacteridae-Actinomycetales-Micrococcineae-Microbacteriaceae-Microbacterium

CAUTION ! One genus only in the extracted sequences. No outgroup *may* be pertinent enough for a good phylogeny. You may need to increase the number of desired sequences or use a *more stringent database*.

Phylogenetic tree analysis

Proximal Cluster ['Microbacterium_lacticum T X77441']

ANALYSIS OF PATRISTIC DISTANCES

Microbacterium_lacticum T X77441 is the closest sequence based on patristic distances

On the basis of patristic Distances, the **closest** sequence is: Microbacterium_lacticum T X77441 AND is in the proximal cluster

No statistics available for patristic distances between members of the species of the closest sequence

Remark: the patristic distance to Microbacterium_lacticum T X77441 is in the 75th percentile of the Microbacterium genus inter-species patristic distances : the sequence may correspond to a member of this genus

---

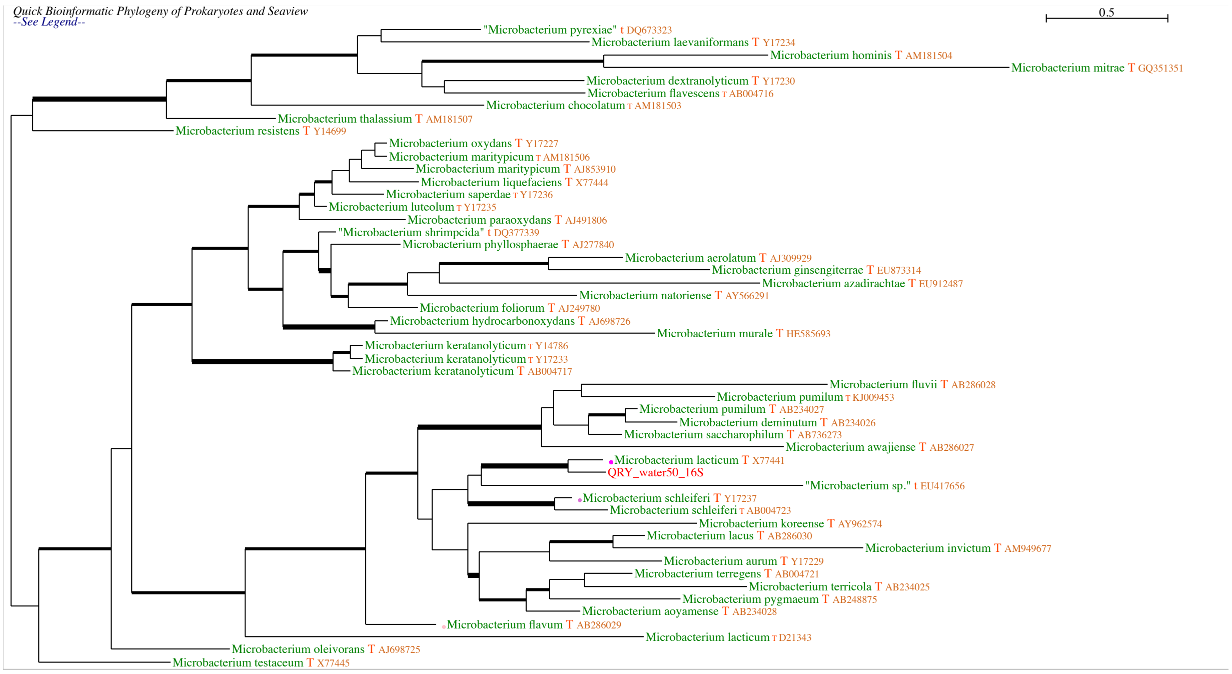

le BIBI QBPP Quick Bioinformatic Phylogeny of Prokaryotes | in short | How-to | Cite | Tests sets | Databases | Contact us | LBBE | prabi

lebibi Quick BioInformatic Phylogeny of Prokaryotes

Summary of the methods and database used during this session

Analysis Extraction of similar sequences for ingroup sequences: Query
There are 44 sequences in your batch
BLAST using the procaryota_SSU-rDNA-16S_TS-stringent database. Trying to extract 50 sequences with expect lower than 0.1
ALIGNMENT using mafft Options : speed oriented
BMGE use for alignment optimization Options :defaults parameters
TREE : phylogenetic tree using fasttree
Evolution model GTR -gamma

RESULTS

Results

Query data

Database: **procaryota_SSU-rDNA-16S_TS-stringent**
Note: The use of the stringent database (all the GenBank sequences for a given species) may lead to errors due to the low quality identification of some sequences, a careful analysis is necessary.
Original name of the query (from fasta) : **QRY_water51_16S.** leBiBI ref : pqpIvwPtL

Sequence composition

Length of Query sequence is : 1281
Nucleotide Composition 338 A - 284 T - 269 C - 390 G - **0 N 0.0 % Gaps % =0.0**
GC%=0.51

Quality of the BLAST analysis

The number of Blast hits is **OK**
All is OK, the best BLAST hit length (**1279**) is =99.8% of the query length

Biodiversity level

All extracted sequences appear to belong to Bacteria and their lowest shared taxon is: Bacteria-Proteobacteria-Gammaproteobacteria-Pseudomonadales-Moraxellaceae
More than one genus in the extracted sequences. An outgroup will be present in the tree.

Phylogenetic tree analysis

Proximal Cluster ['Moraxella_pluranimalium T AM884564', 'Moraxella_lincolnii T FR822735', 'Moraxella_lincolnii T HF558363'] (and 2 others)

ANALYSIS OF PATRISTIC DISTANCES
Absence of the closest sequence based on patristic distances Moraxella_osloensis T JN175341 from the proximal cluster.
Look at the tree and branch support
On the basis of patristic Distances, the **closest TS** sequence is: Moraxella_osloensis T JN175341 AND is NOT in the proximal cluster
The closest sequence has not the same species name as the closest TS
No statistics available for patristic distances between members of the species of the closest sequence
Remark: the patristic distance to Moraxella_osloensis T JN175341 is in the 75th percentile of the Moraxella genus inter-species patristic distances : the sequence may correspond to a member of this genus

---

Quick Bioinformatic Phylogeny of Prokaryotes and Seaview
--See Legend--

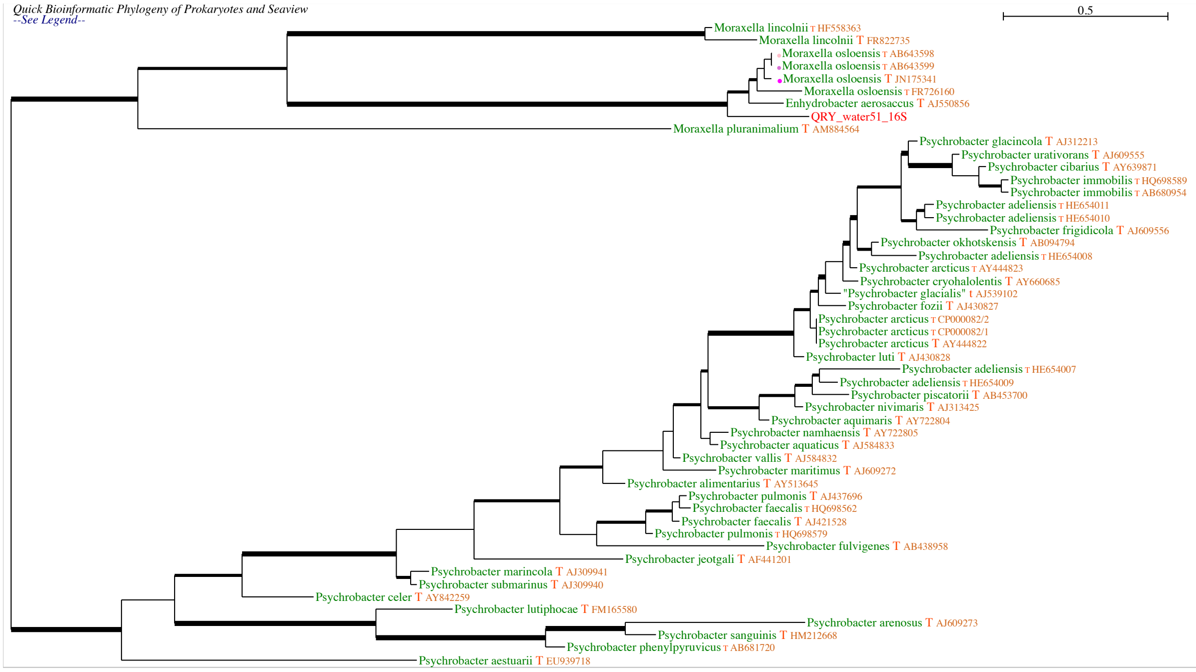

leBIBI QBPP Quick Bioinformatic Phylogeny of Prokaryotes | in short | How-to | Cite | Tests sets | Databases | Contact us | LBBE | prabi

lebibi Quick BioInformatic Phylogeny of Prokaryotes

Summary of the methods and database used during this session

Analysis Extraction of similar sequences for ingroup sequences: Query
There are 44 sequences in your batch
BLAST using the procaryota_SSU-rDNA-16S_TS-stringent database. Trying to extract 50 sequences with expect lower than 0.1
ALIGNMENT using mafft Options : speed oriented
BMGE use for alignment optimization Options :defaults parameters
TREE : phylogenetic tree using fasttree
Evolution model GTR -gamma

RESULTS

Results

Query data

Database: **procaryota_SSU-rDNA-16S_TS-stringent**
Note: The use of the stringent database (all the GenBank sequences for a given species) may lead to errors due to the low quality identification of some sequences, a careful analysis is necessary.
Original name of the query (from fasta) : **QRY_water52_16S.** leBiBI ref : pqpIvwPtL

Sequence composition

Length of Query sequence is : 1278
Nucleotide Composition 307 A - 250 T - 304 C - 417 G - **0 N 0.0 %** **Gaps % =0.0**
GC%=0.56

Quality of the BLAST analysis

The number of Blast hits is **OK**
All is OK, the best BLAST hit length (**1280**) is =100.% of the query length

Biodiversity level

All extracted sequences appear to belong to Bacteria and their lowest shared taxon is: Bacteria-Actinobacteria-Actinobacteridae-Actinomycetales-Micrococcineae-Microbacteriaceae-Microbacterium
CAUTION ! One genus only in the extracted sequences. No outgroup *may* be pertinent enough for a good phylogeny. You may need to increase the number of desired sequences or use a *more stringent database*.

Phylogenetic tree analysis

Proximal Cluster ['Microbacterium_oxydans T Y17227', 'Microbacterium_maritypicum T AJ853910', 'Microbacterium_maritypicum T AM181506']

ANALYSIS OF PATRISTIC DISTANCES
Microbacterium_maritypicum T AM181506 is the closest sequence based on patristic distances
On the basis of patristic Distances, the **closest** sequence is: Microbacterium_maritypicum T AM181506 AND is in the proximal cluster
No statistics available for patristic distances between members of the species of the closest sequence
Remark: the patristic distance to Microbacterium_maritypicum T AM181506 is in the 75th percentile of the Microbacterium genus inter-species patristic distances : the sequence may correspond to a member of this genus

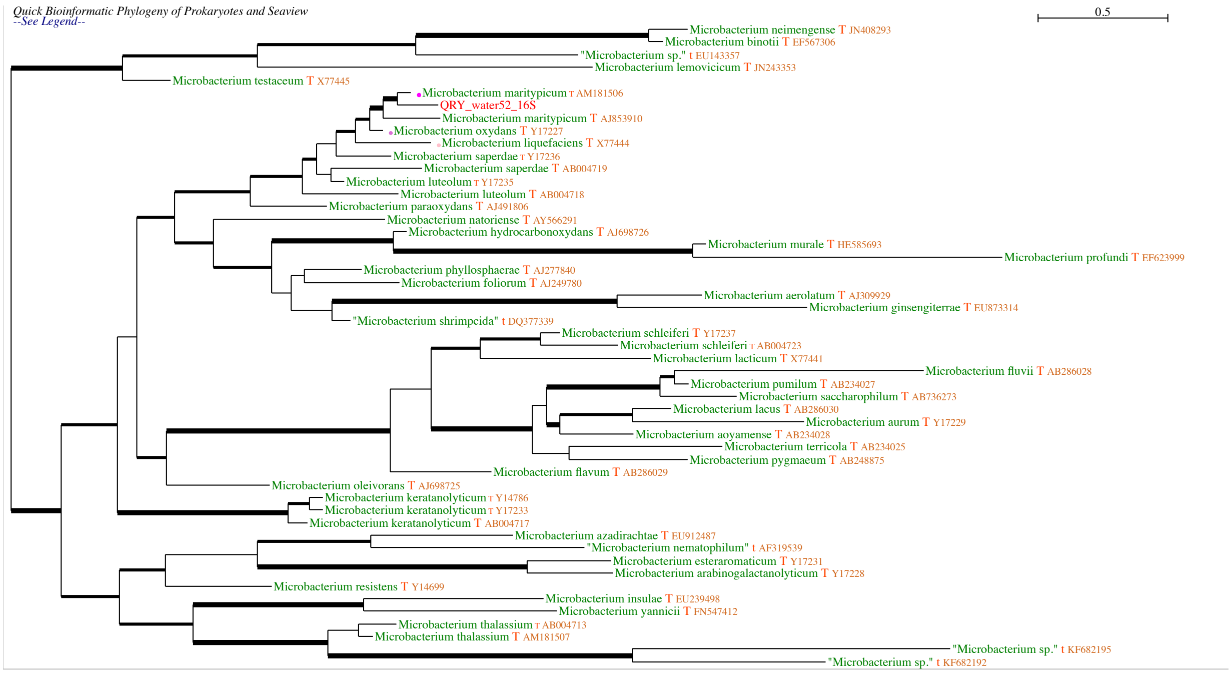

Quick Bioinformatic Phylogeny of Prokaryotes | in short | How-to | Cite | Tests sets | Databases | Contact us

lebibi Quick BioInformatic Phylogeny of Prokaryotes

Summary of the methods and database used during this session

Analysis Extraction of similar sequences for ingroup sequences: Query
There are 44 sequences in your batch
BLAST using the procaryota_SSU-rDNA-16S_TS-stringent database. Trying to extract 50 sequences with expect lower than 0.1
ALIGNMENT using mafft Options : speed oriented
BMGE use for alignment optimization Options :defaults parameters
TREE : phylogenetic tree using fasttree
Evolution model GTR -gamma

RESULTS

Results

Query data

Database: **procaryota_SSU-rDNA-16S_TS-stringent**
Note: The use of the stringent database (all the GenBank sequences for a given species) may lead to errors due to the low quality identification of some sequences, a careful analysis is necessary.
Original name of the query (from fasta) : **QRY_water54_16S.** leBiBI ref : pqpIvwPtL

Sequence composition

Length of Query sequence is : 1256
Nucleotide Composition 314 A - 243 T - 307 C - 380 G - **0 N 0.0 % Gaps % =12.0**
GC%=0.55

Quality of the BLAST analysis

The number of Blast hits is **OK**
The fragment given by BLAST (1097) is shorter than expected: 0.87% of the query length
POSSIBLY DIFFICULT INTERPRETATION
Are you sure of the quality of the query sequence ? Look at the aligment below and to blast suggestions

Biodiversity level

All extracted sequences appear to belong to Bacteria and their lowest shared taxon is: Bacteria-Proteobacteria-Gammaproteobacteria-Xanthomonadales-Xanthomonadaceae
More than one genus in the extracted sequences. An outgroup will be present in the tree.

Phylogenetic tree analysis

Proximal Cluster ['Pseudoxanthomonas_kaohsiungensis T AY650027']

ANALYSIS OF PATRISTIC DISTANCES
Pseudoxanthomonas_kaohsiungensis T AY650027 is the closest sequence based on patristic distances
On the basis of patristic Distances, the **closest** sequence is: Pseudoxanthomonas_kaohsiungensis T AY650027 AND is in the proximal cluster
No statistics available for patristic distances between members of the species of the closest sequence
Remark: the patristic distance to Pseudoxanthomonas_kaohsiungensis T AY650027 is OUTSIDE the 75th percentile of the Pseudoxanthomonas inter-species patristic distances : the sequence may not correspond to this genus or is of very low quality

---

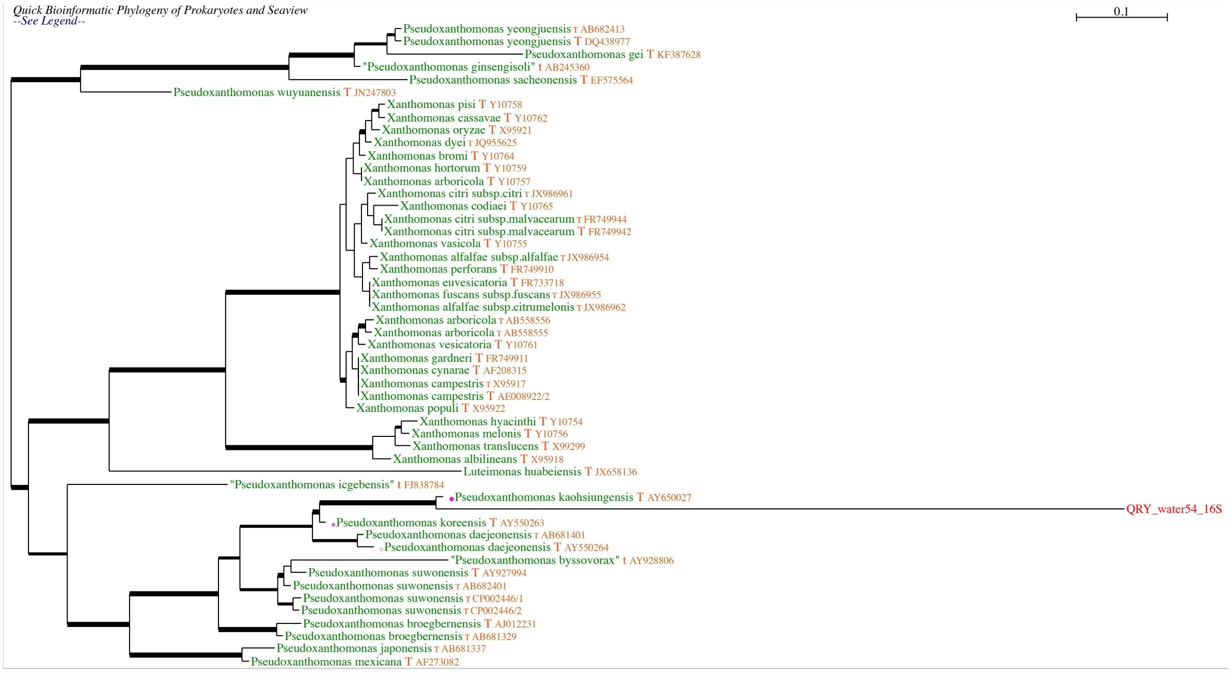

lebibi Quick BioInformatic Phylogeny of Prokaryotes

Summary of the methods and database used during this session

Analysis Extraction of similar sequences for ingroup sequences: Query
There are 44 sequences in your batch
BLAST using the procaryota_SSU-rDNA-16S_TS-stringent database. Trying to extract 50 sequences with expect lower than 0.1
ALIGNMENT using mafft Options : speed oriented
BMGE use for alignment optimization Options :defaults parameters
TREE : phylogenetic tree using fasttree
Evolution model GTR -gamma

RESULTS

Results

Query data

Database: **procaryota_SSU-rDNA-16S_TS-stringent**
Note: The use of the stringent database (all the GenBank sequences for a given species) may lead to errors due to the low quality identification of some sequences, a careful analysis is necessary.
Original name of the query (from fasta) : **QRY_water55_16S.** leBiBI ref : pqpIvwPtL

Sequence composition

Length of Query sequence is : 1212
Nucleotide Composition 292 A - 253 T - 282 C - 385 G - **0 N 0.0 % Gaps % =0.0**
GC%=0.55

Quality of the BLAST analysis

The number of Blast hits is **OK**
All is OK, the best BLAST hit length (**1213**) is =100.% of the query length

Biodiversity level

All extracted sequences appear to belong to Bacteria and their lowest shared taxon is: Bacteria-Proteobacteria-Alphaproteobacteria
More than one genus in the extracted sequences. An outgroup will be present in the tree.

Phylogenetic tree analysis

Proximal Cluster ['Sphingomonas_adhaesiva T AB680766', 'Sphingomonas_adhaesiva T D16146', 'Sphingomonas_ginsenosidimutans T HM204925']

ANALYSIS OF PATRISTIC DISTANCES
Sphingomonas_ginsenosidimutans T HM204925 is the closest sequence based on patristic distances
On the basis of patristic Distances, the **closest** sequence is: Sphingomonas_ginsenosidimutans T HM204925 AND is in the proximal cluster
No statistics available for patristic distances between members of the species of the closest sequence
Remark: the patristic distance to Sphingomonas_ginsenosidimutans T HM204925 is in the 75th percentile of the Sphingomonas genus inter-species patristic distances : the sequence may correspond to a member of this genus

---

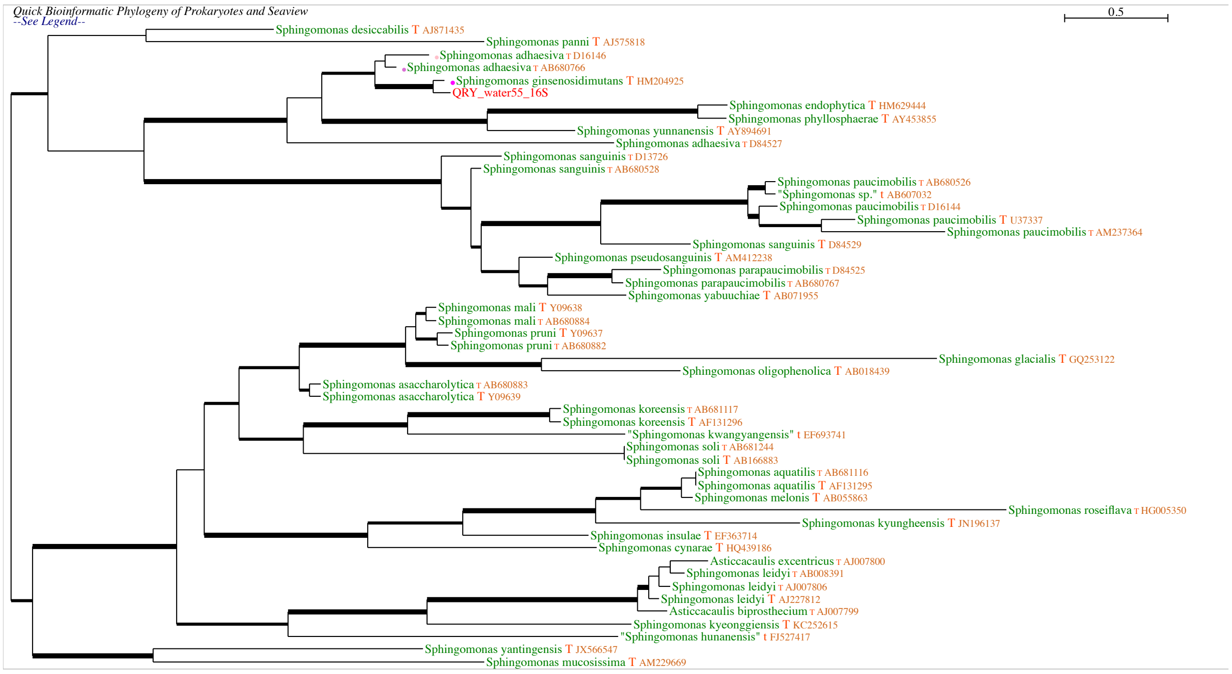

in short | How-to | Cite | Tests sets | Databases | Contact us

lebibi Quick BioInformatic Phylogeny of Prokaryotes

Summary of the methods and database used during this session

Analysis Extraction of similar sequences for ingroup sequences: Query
There are 44 sequences in your batch
BLAST using the procaryota_SSU-rDNA-16S_TS-stringent database. Trying to extract 50 sequences with expect lower than 0.1
ALIGNMENT using mafft Options : speed oriented
BMGE use for alignment optimization Options :defaults parameters
TREE : phylogenetic tree using fasttree
Evolution model GTR -gamma

RESULTS

Results

Query data

Database: **procaryota_SSU-rDNA-16S_TS-stringent**
Note: The use of the stringent database (all the GenBank sequences for a given species) may lead to errors due to the low quality identification of some sequences, a careful analysis is necessary.
Original name of the query (from fasta) : **QRY_water34_16S.** leBiBI ref : pqpIvwPtL

Sequence composition

Length of Query sequence is : 1274
Nucleotide Composition 318 A - 247 T - 302 C - 407 G - **0 N 0.0 % Gaps % =0.0**
GC%=0.55

Quality of the BLAST analysis

The number of Blast hits is **OK**
All is OK, the best BLAST hit length (**1274**) is =100.% of the query length

Biodiversity level

All extracted sequences appear to belong to Bacteria and their lowest shared taxon is: Bacteria-Actinobacteria-Actinobacteridae-Actinomycetales-Micrococcineae-Microbacteriaceae
More than one genus in the extracted sequences. An outgroup will be present in the tree.

Phylogenetic tree analysis

Proximal Cluster ['Microbacterium_hatanonis T AB274908', 'Microbacterium_aurum T Y17229']

ANALYSIS OF PATRISTIC DISTANCES
Microbacterium_aurum T Y17229 is the closest sequence based on patristic distances
On the basis of patristic Distances, the **closest** sequence is: Microbacterium_aurum T Y17229 AND is in the proximal cluster
No statistics available for patristic distances between members of the species of the closest sequence
Remark: the patristic distance to Microbacterium_aurum T Y17229 is in the 75th percentile of the Microbacterium genus inter-species patristic distances : the sequence may correspond to a member of this genus

---

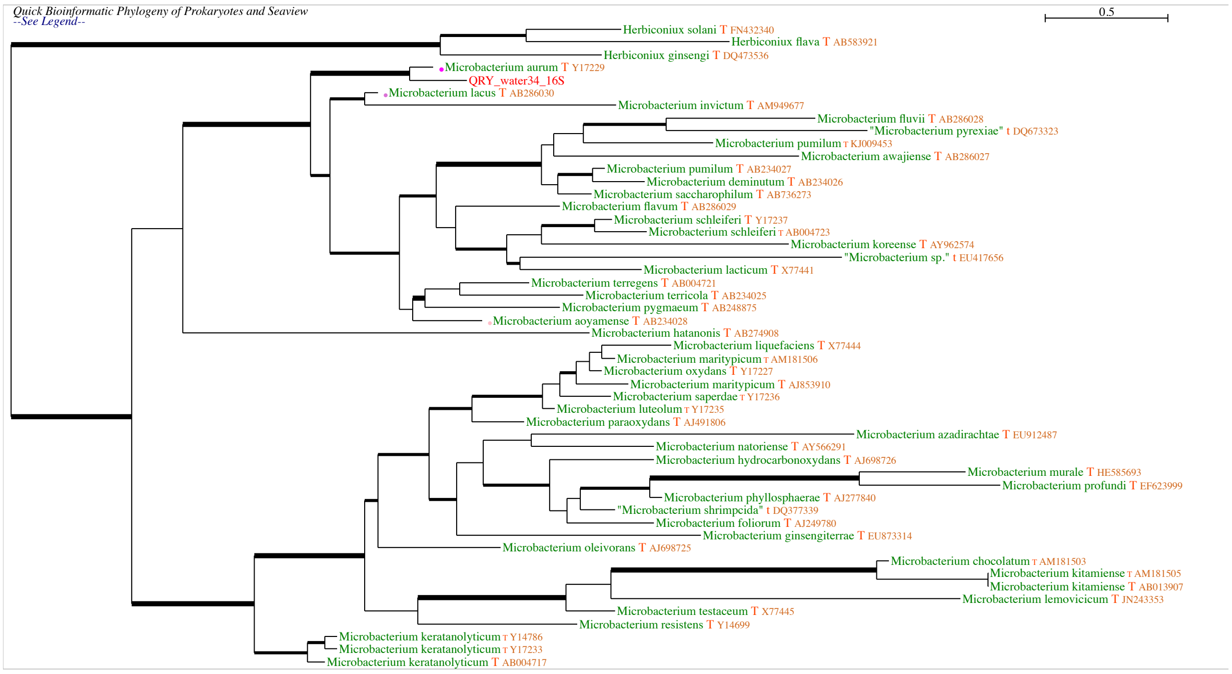

le BIBI QBPP Quick Bioinformatic Phylogeny of Prokaryotes | in short | How-to | Cite | Tests sets | Databases | Contact us | LBBE | prabi

lebibi Quick BioInformatic Phylogeny of Prokaryotes

Summary of the methods and database used during this session

Analysis Extraction of similar sequences for ingroup sequences: Query
There are 44 sequences in your batch
BLAST using the procaryota_SSU-rDNA-16S_TS-stringent database. Trying to extract 50 sequences with expect lower than 0.1
ALIGNMENT using mafft Options : speed oriented
BMGE use for alignment optimization Options :defaults parameters
TREE : phylogenetic tree using fasttree
Evolution model GTR -gamma

RESULTS

Results

Query data

Database: **procaryota_SSU-rDNA-16S_TS-stringent**
Note: The use of the stringent database (all the GenBank sequences for a given species) may lead to errors due to the low quality identification of some sequences, a careful analysis is necessary.
Original name of the query (from fasta) : **QRY_water57_16S.** leBiBI ref : pqpIvwPtL

Sequence composition

Length of Query sequence is : 1276
Nucleotide Composition 298 A - 254 T - 300 C - 424 G - **0 N 0.0 %** **Gaps % =0.0**
GC%=0.56

Quality of the BLAST analysis

The number of Blast hits is **OK**
All is OK, the best BLAST hit length (**1271**) is =99.6% of the query length

Biodiversity level

All extracted sequences appear to belong to Bacteria and their lowest shared taxon is: Bacteria-Actinobacteria-Actinobacteridae-Actinomycetales-Micrococcineae-Microbacteriaceae-Microbacterium
CAUTION ! One genus only in the extracted sequences. No outgroup *may* be pertinent enough for a good phylogeny. You may need to increase the number of desired sequences or use a *more stringent database*.

Phylogenetic tree analysis

Incertae sedis **['NO_ACCEPTABLE_CLUSTER']**
No clear proximal cluster; use the tree for determination, if possible
No acceptable solution

ANALYSIS OF PATRISTIC DISTANCES
Closest sequence based on patristic distances Microbacterium_trichothecenolyticum T AB004722
Remark: the patristic distance to Microbacterium_trichothecenolyticum T AB004722 is in the 75th percentile of the Microbacterium genus inter-species patristic distances : the sequence may correspond to a member of this genus
The closest sequence based on patristic distances Microbacterium_trichothecenolyticum T AB004722 is NOT the FIRST BLAST hit
The closest sequence based on patristic distances Microbacterium_trichothecenolyticum T AB004722 is NOT in the top 5 BLAST hits

---

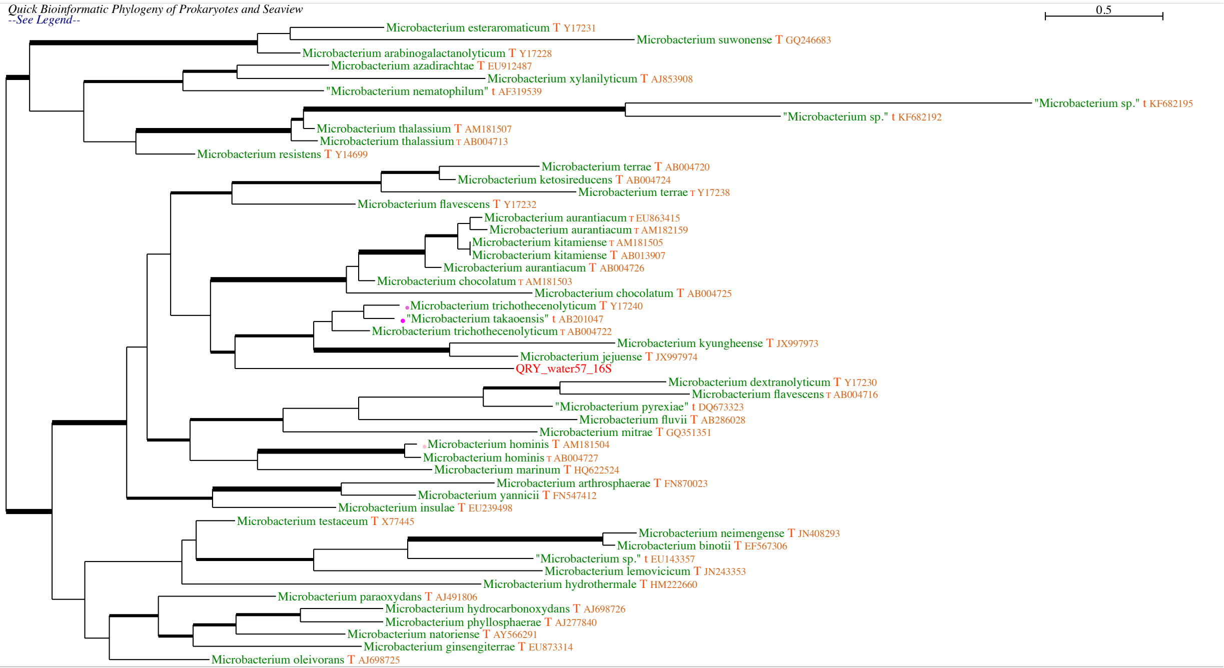

in short | How-to | Cite | Tests sets | Databases | Contact us

lebibi Quick BioInformatic Phylogeny of Prokaryotes

Summary of the methods and database used during this session

Analysis Extraction of similar sequences for ingroup sequences: Query
There are 44 sequences in your batch
BLAST using the procaryota_SSU-rDNA-16S_TS-stringent database. Trying to extract 50 sequences with expect lower than 0.1
ALIGNMENT using mafft Options : speed oriented
BMGE use for alignment optimization Options :defaults parameters
TREE : phylogenetic tree using fasttree
Evolution model GTR -gamma

RESULTS

Results

Query data

Database: **procaryota_SSU-rDNA-16S_TS-stringent**
Note: The use of the stringent database (all the GenBank sequences for a given species) may lead to errors due to the low quality identification of some sequences, a careful analysis is necessary.
Original name of the query (from fasta) : **QRY_water58_16S.** leBiBI ref : pqpIvwPtL

Sequence composition

Length of Query sequence is : 1269
Nucleotide Composition 312 A - 243 T - 303 C - 411 G - **0 N 0.0 %** **Gaps % =0.0**
GC%=0.56

Quality of the BLAST analysis

The number of Blast hits is **OK**
All is OK, the best BLAST hit length (**1269**) is =100.% of the query length

Biodiversity level

All extracted sequences appear to belong to Bacteria and their lowest shared taxon is: Bacteria-Actinobacteria-Actinobacteridae-Actinomycetales-Micrococcineae-Microbacteriaceae-Microbacterium
CAUTION ! One genus only in the extracted sequences. No outgroup *may* be pertinent enough for a good phylogeny. You may need to increase the number of desired sequences or use a *more stringent database*.

Phylogenetic tree analysis

Proximal Cluster ['Microbacterium_flavum T AB286029', 'Microbacterium_lacticum T X77441']

ANALYSIS OF PATRISTIC DISTANCES
Microbacterium_lacticum T X77441 is the closest sequence based on patristic distances
On the basis of patristic Distances, the **closest** sequence is: Microbacterium_lacticum T X77441 AND is in the proximal cluster
No statistics available for patristic distances between members of the species of the closest sequence
Remark: the patristic distance to Microbacterium_lacticum T X77441 is in the 75th percentile of the Microbacterium genus inter-species patristic distances : the sequence may correspond to a member of this genus

---

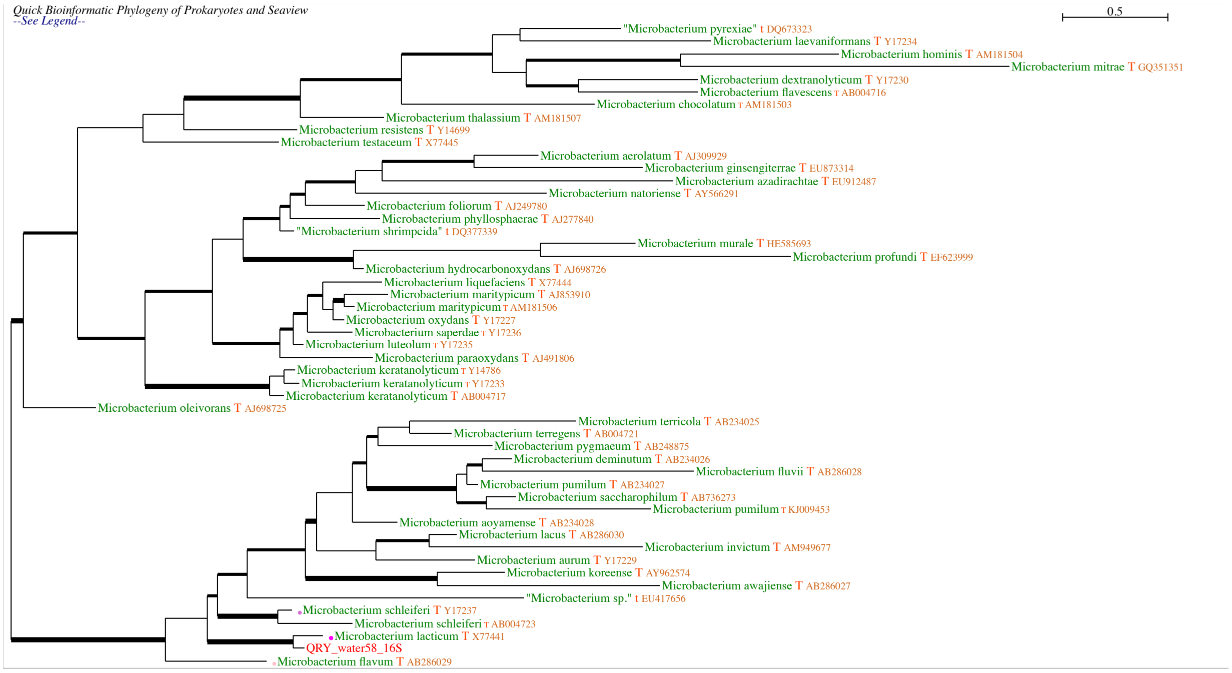

in short | How-to | Cite | Tests sets | Databases | Contact us

lebibi Quick BioInformatic Phylogeny of Prokaryotes

Summary of the methods and database used during this session

Analysis Extraction of similar sequences for ingroup sequences: Query
There are 44 sequences in your batch
BLAST using the procaryota_SSU-rDNA-16S_TS-stringent database. Trying to extract 50 sequences with expect lower than 0.1
ALIGNMENT using mafft Options : speed oriented
BMGE use for alignment optimization Options :defaults parameters
TREE : phylogenetic tree using fasttree
Evolution model GTR -gamma

RESULTS

Results

Query data

Database: **procaryota_SSU-rDNA-16S_TS-stringent**
Note: The use of the stringent database (all the GenBank sequences for a given species) may lead to errors due to the low quality identification of some sequences, a careful analysis is necessary.
Original name of the query (from fasta) : **QRY_water60_16S.** leBiBI ref : pqpIvwPtL

Sequence composition

Length of Query sequence is : 1283
Nucleotide Composition 316 A - 248 T - 306 C - 413 G - **0 N 0.0 %** **Gaps % =0.0**
GC%=0.56

Quality of the BLAST analysis

The number of Blast hits is **OK**
All is OK, the best BLAST hit length (**1281**) is =99.8% of the query length

Biodiversity level

All extracted sequences appear to belong to Bacteria and their lowest shared taxon is: Bacteria-Actinobacteria-Actinobacteridae-Actinomycetales-Micrococcineae-Microbacteriaceae-Microbacterium
CAUTION ! One genus only in the extracted sequences. No outgroup *may* be pertinent enough for a good phylogeny. You may need to increase the number of desired sequences or use a *more stringent database*.

Phylogenetic tree analysis

Proximal Cluster ['Microbacterium_lacticum T X77441']

ANALYSIS OF PATRISTIC DISTANCES
Microbacterium_lacticum T X77441 is the closest sequence based on patristic distances
On the basis of patristic Distances, the **closest** sequence is: Microbacterium_lacticum T X77441 AND is in the proximal cluster
No statistics available for patristic distances between members of the species of the closest sequence
Remark: the patristic distance to Microbacterium_lacticum T X77441 is in the 75th percentile of the Microbacterium genus inter-species patristic distances : the sequence may correspond to a member of this genus

---

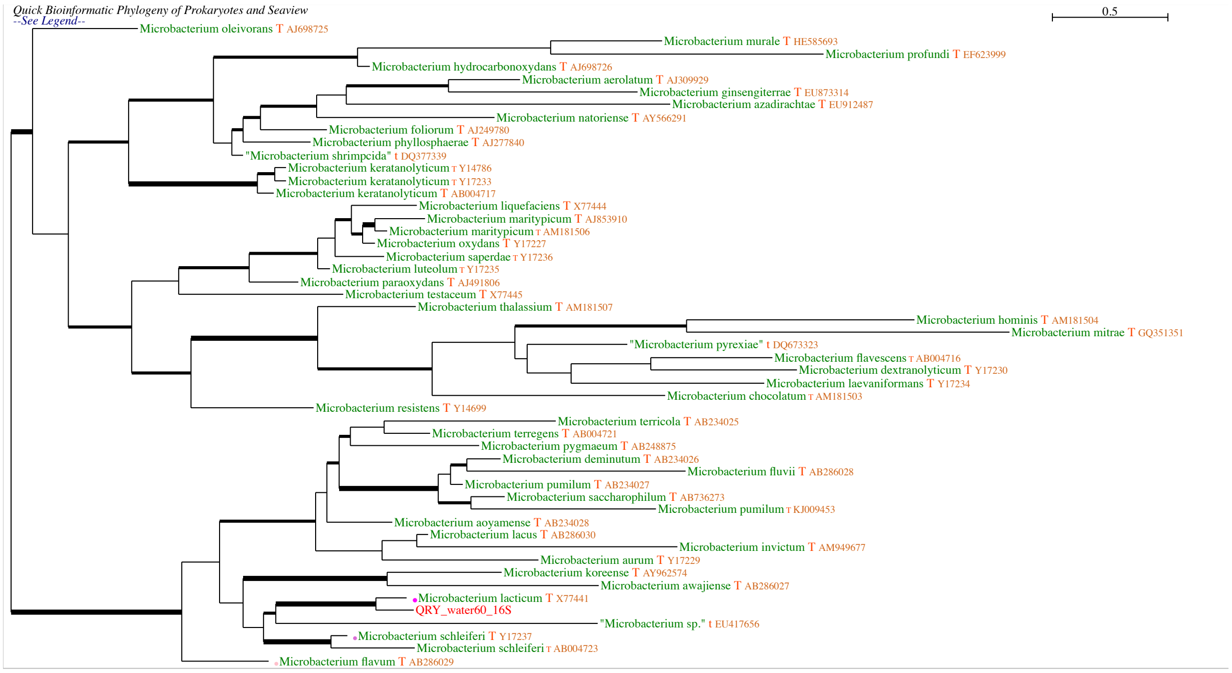

in short | How-to | Cite | Tests sets | Databases | Contact us

lebibi Quick BioInformatic Phylogeny of Prokaryotes

Summary of the methods and database used during this session

Analysis Extraction of similar sequences for ingroup sequences: Query
There are 44 sequences in your batch
BLAST using the procaryota_SSU-rDNA-16S_TS-stringent database. Trying to extract 50 sequences with expect lower than 0.1
ALIGNMENT using mafft Options : speed oriented
BMGE use for alignment optimization Options :defaults parameters
TREE : phylogenetic tree using fasttree
Evolution model GTR -gamma

RESULTS

Results

Query data

Database: **procaryota_SSU-rDNA-16S_TS-stringent**
Note: The use of the stringent database (all the GenBank sequences for a given species) may lead to errors due to the low quality identification of some sequences, a careful analysis is necessary.
Original name of the query (from fasta) : **QRY_water61_16S.** leBiBI ref : pqpIvwPtL

Sequence composition

Length of Query sequence is : 1275
Nucleotide Composition 307 A - 249 T - 304 C - 415 G - **0 N 0.0 % Gaps % =0.0**
GC%=0.56

Quality of the BLAST analysis

The number of Blast hits is **OK**
All is OK, the best BLAST hit length (**1273**) is =99.8% of the query length

Biodiversity level

All extracted sequences appear to belong to Bacteria and their lowest shared taxon is: Bacteria-Actinobacteria-Actinobacteridae-Actinomycetales-Micrococcineae-Microbacteriaceae-Microbacterium
CAUTION ! One genus only in the extracted sequences. No outgroup *may* be pertinent enough for a good phylogeny. You may need to increase the number of desired sequences or use a *more stringent database*.

Phylogenetic tree analysis

Proximal Cluster ['Microbacterium_maritypicum T AM181506']

ANALYSIS OF PATRISTIC DISTANCES
Microbacterium_maritypicum T AM181506 is the closest sequence based on patristic distances
On the basis of patristic Distances, the **closest** sequence is: Microbacterium_maritypicum T AM181506 AND is in the proximal cluster
No statistics available for patristic distances between members of the species of the closest sequence
Remark: the patristic distance to Microbacterium_maritypicum T AM181506 is in the 75th percentile of the Microbacterium genus inter-species patristic distances : the sequence may correspond to a member of this genus

---

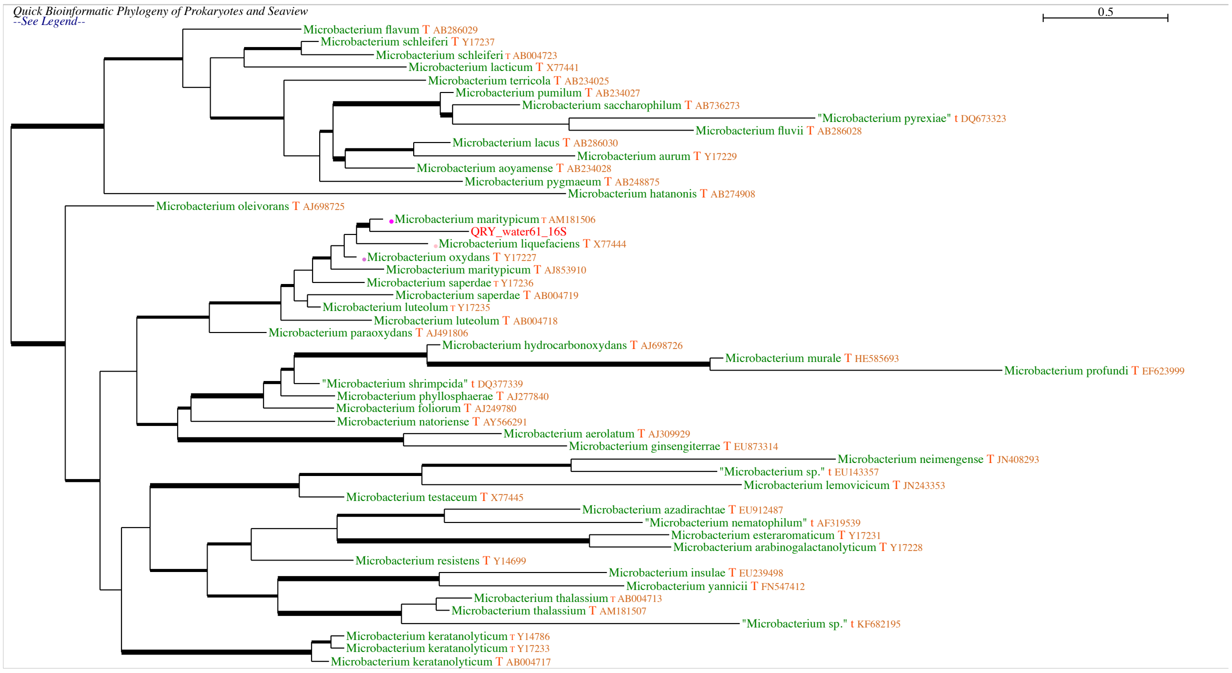

in short | How-to | Cite | Tests sets | Databases | Contact us

lebibi Quick BioInformatic Phylogeny of Prokaryotes

Summary of the methods and database used during this session

Analysis Extraction of similar sequences for ingroup sequences: Query
There are 44 sequences in your batch
BLAST using the procaryota_SSU-rDNA-16S_TS-stringent database. Trying to extract 50 sequences with expect lower than 0.1
ALIGNMENT using mafft Options : speed oriented
BMGE use for alignment optimization Options :defaults parameters
TREE : phylogenetic tree using fasttree
Evolution model GTR -gamma

RESULTS

Results

Query data

Database: **procaryota_SSU-rDNA-16S_TS-stringent**
Note: The use of the stringent database (all the GenBank sequences for a given species) may lead to errors due to the low quality identification of some sequences, a careful analysis is necessary.
Original name of the query (from fasta) : **QRY_water62_16S.** leBiBI ref : pqpIvwPtL

Sequence composition

Length of Query sequence is : 1279
Nucleotide Composition 315 A - 243 T - 306 C - 415 G - **0 N 0.0 %** **Gaps % =0.0**
GC%=0.56

Quality of the BLAST analysis

The number of Blast hits is **OK**
All is OK, the best BLAST hit length (**1277**) is =99.8% of the query length

Biodiversity level

All extracted sequences appear to belong to Bacteria and their lowest shared taxon is: Bacteria-Actinobacteria-Actinobacteridae-Actinomycetales-Micrococcineae-Microbacteriaceae-Microbacterium
CAUTION ! One genus only in the extracted sequences. No outgroup *may* be pertinent enough for a good phylogeny. You may need to increase the number of desired sequences or use a *more stringent database*.

Phylogenetic tree analysis

Proximal Cluster ['Microbacterium_lacticum T X77441']

ANALYSIS OF PATRISTIC DISTANCES
Microbacterium_lacticum T X77441 is the closest sequence based on patristic distances
On the basis of patristic Distances, the **closest** sequence is: Microbacterium_lacticum T X77441 AND is in the proximal cluster
No statistics available for patristic distances between members of the species of the closest sequence
Remark: the patristic distance to Microbacterium_lacticum T X77441 is in the 75th percentile of the Microbacterium genus inter-species patristic distances : the sequence may correspond to a member of this genus

---

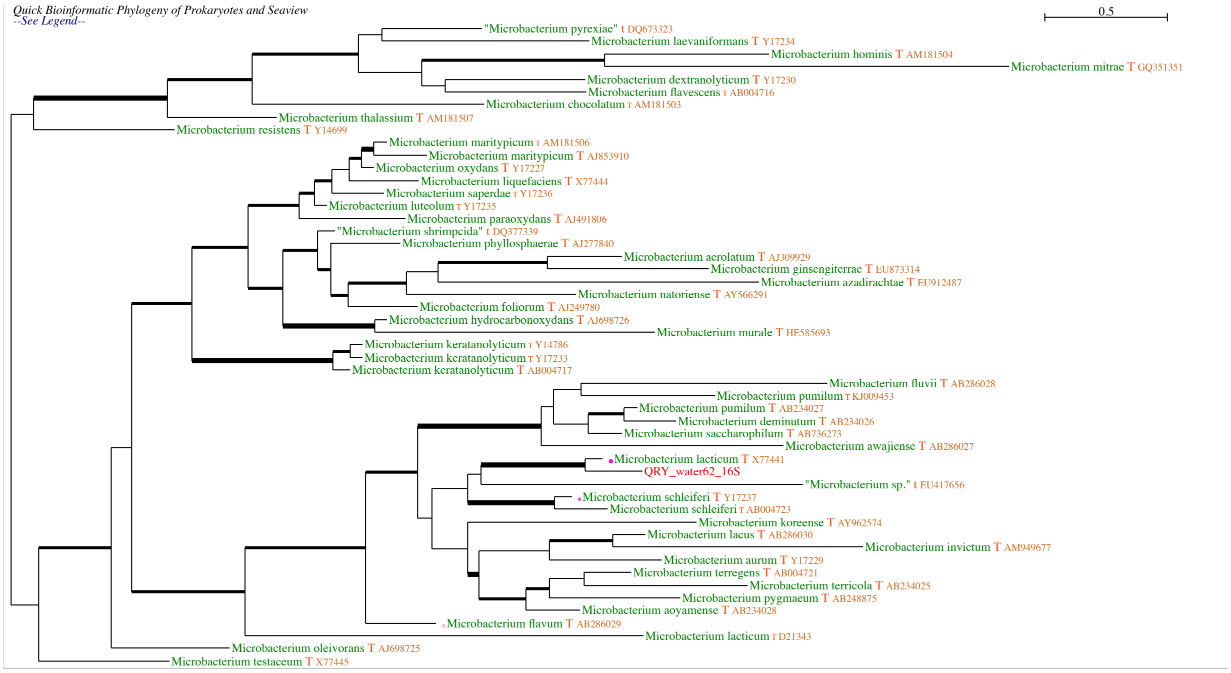

lebibi Quick BioInformatic Phylogeny of Prokaryotes

Summary of the methods and database used during this session

Analysis Extraction of similar sequences for ingroup sequences: Query
There are 44 sequences in your batch
BLAST using the procaryota_SSU-rDNA-16S_TS-stringent database. Trying to extract 50 sequences with expect lower than 0.1
ALIGNMENT using mafft Options : speed oriented
BMGE use for alignment optimization Options :defaults parameters
TREE : phylogenetic tree using fasttree
Evolution model GTR -gamma

RESULTS

Results

Query data

Database: **procaryota_SSU-rDNA-16S_TS-stringent**
Note: The use of the stringent database (all the GenBank sequences for a given species) may lead to errors due to the low quality identification of some sequences, a careful analysis is necessary.
Original name of the query (from fasta) : **QRY_water63_16S.** leBiBI ref : pqpIvwPtL

Sequence composition

Length of Query sequence is : 1229
Nucleotide Composition 297 A - 259 T - 282 C - 387 G - **0 N 0.0 % Gaps % =4.0**
GC%=0.54

Quality of the BLAST analysis

The number of Blast hits is **OK**
All is OK, the best BLAST hit length (**1211**) is =98.5% of the query length

Biodiversity level

All extracted sequences appear to belong to Bacteria and their lowest shared taxon is: Bacteria-Proteobacteria-Alphaproteobacteria-Sphingomonadales-Sphingomonadaceae
More than one genus in the extracted sequences. An outgroup will be present in the tree.

Phylogenetic tree analysis

Proximal Cluster ['Sphingomonas_sp. ? T AB607032', 'Sphingomonas_paucimobilis T AM237364', 'Sphingomonas_paucimobilis T U20776']

ANALYSIS OF PATRISTIC DISTANCES
Sphingomonas_sp. ? T AB607032 is the closest sequence based on patristic distances
On the basis of patristic Distances, the **closest TS** sequence is: Sphingomonas_paucimobilis T AB680526 AND is NOT in the proximal cluster
The closest sequence has not the same species name as the closest TS
No statistics available for patristic distances between members of the species of the closest sequence
Remark: the patristic distance to Sphingomonas_sp. ? T AB607032 is in the 75th percentile of the Sphingomonas genus inter-species patristic distances : the sequence may correspond to a member of this genus

---

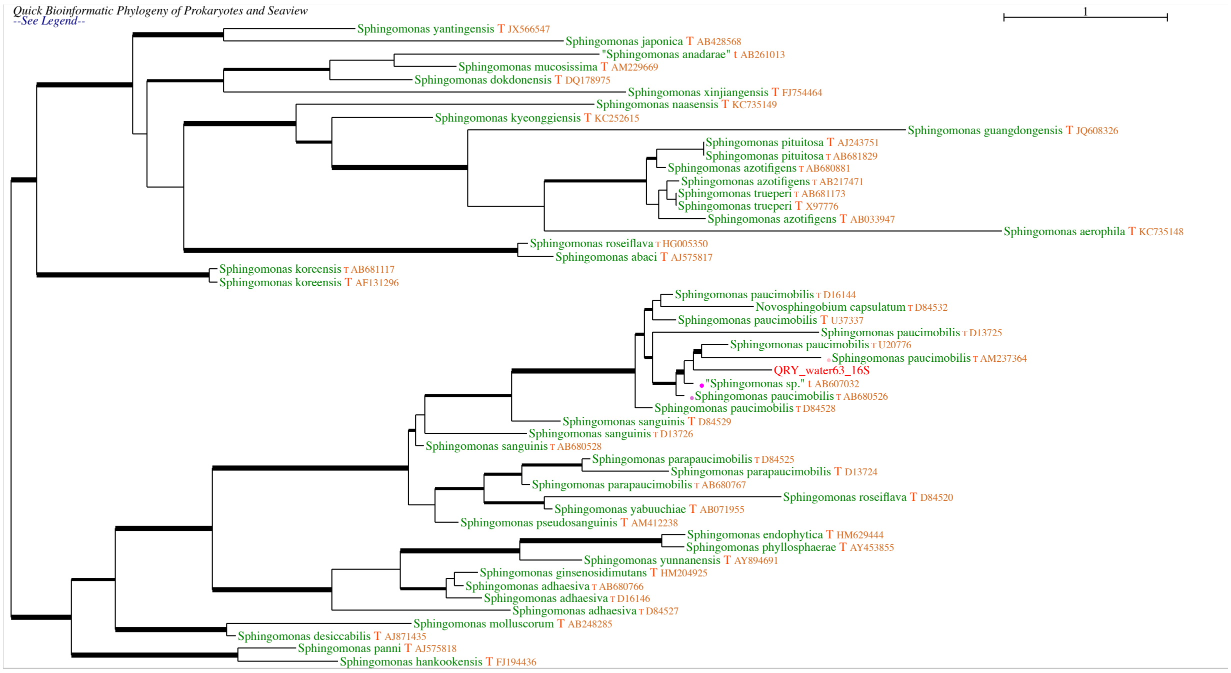

le BIBI QBPP Quick Bioinformatic Phylogeny of Prokaryotes | in short | How-to | Cite | Tests sets | Databases | Contact us | LBBE | prabi

lebibi Quick BioInformatic Phylogeny of Prokaryotes

Summary of the methods and database used during this session

Analysis Extraction of similar sequences for ingroup sequences: Query
There are 44 sequences in your batch
BLAST using the procaryota_SSU-rDNA-16S_TS-stringent database. Trying to extract 50 sequences with expect lower than 0.1
ALIGNMENT using mafft Options : speed oriented
BMGE use for alignment optimization Options :defaults parameters
TREE : phylogenetic tree using fasttree
Evolution model GTR -gamma

RESULTS

Results

Query data

Database: **procaryota_SSU-rDNA-16S_TS-stringent**
Note: The use of the stringent database (all the GenBank sequences for a given species) may lead to errors due to the low quality identification of some sequences, a careful analysis is necessary.
Original name of the query (from fasta) : **QRY_water64_16S.** leBiBI ref : pqpIvwPtL

Sequence composition

Length of Query sequence is : 1282
Nucleotide Composition 335 A - 254 T - 297 C - 396 G - **0 N 0.0 %** **Gaps % =0.0**
GC%=0.54

Quality of the BLAST analysis

The number of Blast hits is **OK**
All is OK, the best BLAST hit length (**1280**) is =99.8% of the query length

Biodiversity level

All extracted sequences appear to belong to Bacteria and their lowest shared taxon is: Bacteria-Proteobacteria-Betaproteobacteria-Burkholderiales
More than one genus in the extracted sequences. An outgroup will be present in the tree.

Phylogenetic tree analysis

Proximal Cluster ['Variovorax_ginsengisoli T AB245358', 'Variovorax_paradoxus T AB680784', 'Variovorax_paradoxus T AB622219'] (and 3 others)

ANALYSIS OF PATRISTIC DISTANCES
Variovorax_boronicumulans T AB681954 is the closest sequence based on patristic distances
On the basis of patristic Distances, the **closest** sequence is: Variovorax_boronicumulans T AB681954 AND is in the proximal cluster
No statistics available for patristic distances between members of the species of the closest sequence
Remark: the patristic distance to Variovorax_boronicumulans T AB681954 is in the 75th percentile of the Variovorax genus inter-species patristic distances : the sequence may correspond to a member of this genus

---

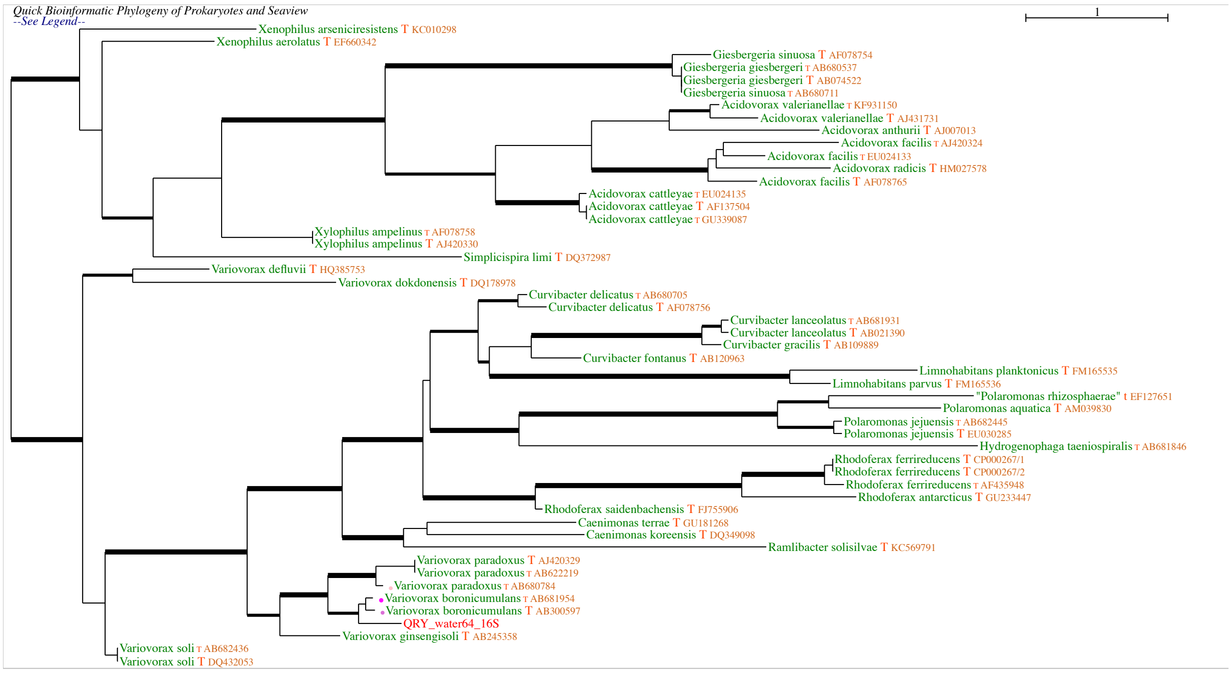

lebibi Quick BioInformatic Phylogeny of Prokaryotes

Summary of the methods and database used during this session

Analysis Extraction of similar sequences for ingroup sequences: Query
There are 44 sequences in your batch
BLAST using the procaryota_SSU-rDNA-16S_TS-stringent database. Trying to extract 50 sequences with expect lower than 0.1
ALIGNMENT using mafft Options : speed oriented
BMGE use for alignment optimization Options :defaults parameters
TREE : phylogenetic tree using fasttree
Evolution model GTR -gamma

RESULTS

Results

Query data

Database: **procaryota_SSU-rDNA-16S_TS-stringent**
Note: The use of the stringent database (all the GenBank sequences for a given species) may lead to errors due to the low quality identification of some sequences, a careful analysis is necessary.
Original name of the query (from fasta) : **QRY_water66_16S.** leBiBI ref : pqpIvwPtL

Sequence composition

Length of Query sequence is : 1282
Nucleotide Composition 335 A - 254 T - 296 C - 397 G - **0 N 0.0 %** **Gaps % =0.0**
GC%=0.54

Quality of the BLAST analysis

The number of Blast hits is **OK**
All is OK, the best BLAST hit length (**1282**) is =100.% of the query length

Biodiversity level

All extracted sequences appear to belong to Bacteria and their lowest shared taxon is: Bacteria-Proteobacteria-Betaproteobacteria-Burkholderiales
More than one genus in the extracted sequences. An outgroup will be present in the tree.

Phylogenetic tree analysis

Proximal Cluster ['Variovorax_boronicumulans T AB300597', 'Variovorax_boronicumulans T AB681954']

ANALYSIS OF PATRISTIC DISTANCES
Variovorax_boronicumulans T AB681954 is the closest sequence based on patristic distances
On the basis of patristic Distances, the **closest** sequence is: Variovorax_boronicumulans T AB681954 AND is in the proximal cluster
No statistics available for patristic distances between members of the species of the closest sequence
Remark: the patristic distance to Variovorax_boronicumulans T AB681954 is in the 75th percentile of the Variovorax genus inter-species patristic distances : the sequence may correspond to a member of this genus

---

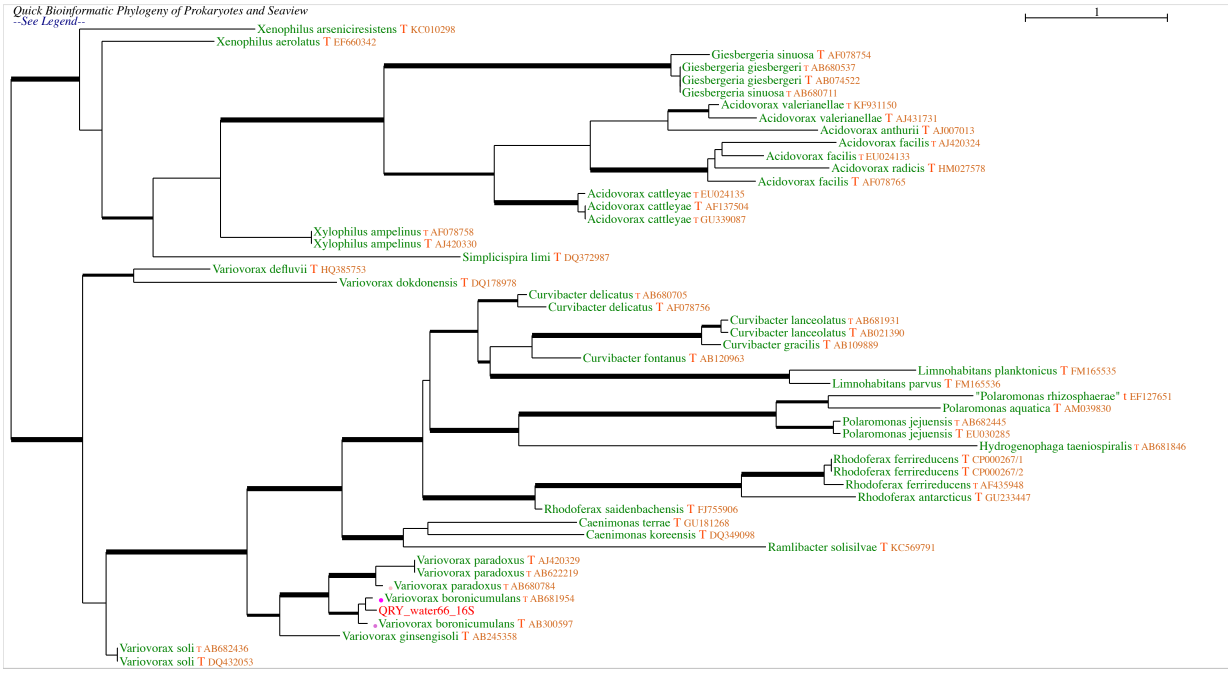

in short | How-to | Cite | Tests sets | Databases | Contact us

lebibi Quick BioInformatic Phylogeny of Prokaryotes

Summary of the methods and database used during this session

Analysis Extraction of similar sequences for ingroup sequences: Query
There are 44 sequences in your batch
BLAST using the procaryota_SSU-rDNA-16S_TS-stringent database. Trying to extract 50 sequences with expect lower than 0.1
ALIGNMENT using mafft Options : speed oriented
BMGE use for alignment optimization Options :defaults parameters
TREE : phylogenetic tree using fasttree
Evolution model GTR -gamma

RESULTS

Results

Query data

Database: **procaryota_SSU-rDNA-16S_TS-stringent**
Note: The use of the stringent database (all the GenBank sequences for a given species) may lead to errors due to the low quality identification of some sequences, a careful analysis is necessary.
Original name of the query (from fasta) : **QRY_water67_16S.** leBiBI ref : pqpIvwPtL

Sequence composition

Length of Query sequence is : 1269
Nucleotide Composition 304 A - 247 T - 302 C - 416 G - **0 N 0.0 %** **Gaps % =0.0**
GC%=0.56

Quality of the BLAST analysis

The number of Blast hits is **OK**
All is OK, the best BLAST hit length (**1269**) is =100.% of the query length

Biodiversity level

All extracted sequences appear to belong to Bacteria and their lowest shared taxon is: Bacteria-Actinobacteria-Actinobacteridae-Actinomycetales-Micrococcineae-Microbacteriaceae-Microbacterium
CAUTION ! One genus only in the extracted sequences. No outgroup *may* be pertinent enough for a good phylogeny. You may need to increase the number of desired sequences or use a *more stringent database*.

Phylogenetic tree analysis

Proximal Cluster ['Microbacterium_saperdae T Y17236', 'Microbacterium_oxydans T Y17227', 'Microbacterium_maritypicum T AM181506']

ANALYSIS OF PATRISTIC DISTANCES
Microbacterium_maritypicum T AM181506 is the closest sequence based on patristic distances
On the basis of patristic Distances, the **closest** sequence is: Microbacterium_maritypicum T AM181506 AND is in the proximal cluster
No statistics available for patristic distances between members of the species of the closest sequence
Remark: the patristic distance to Microbacterium_maritypicum T AM181506 is in the 75th percentile of the Microbacterium genus inter-species patristic distances : the sequence may correspond to a member of this genus

---

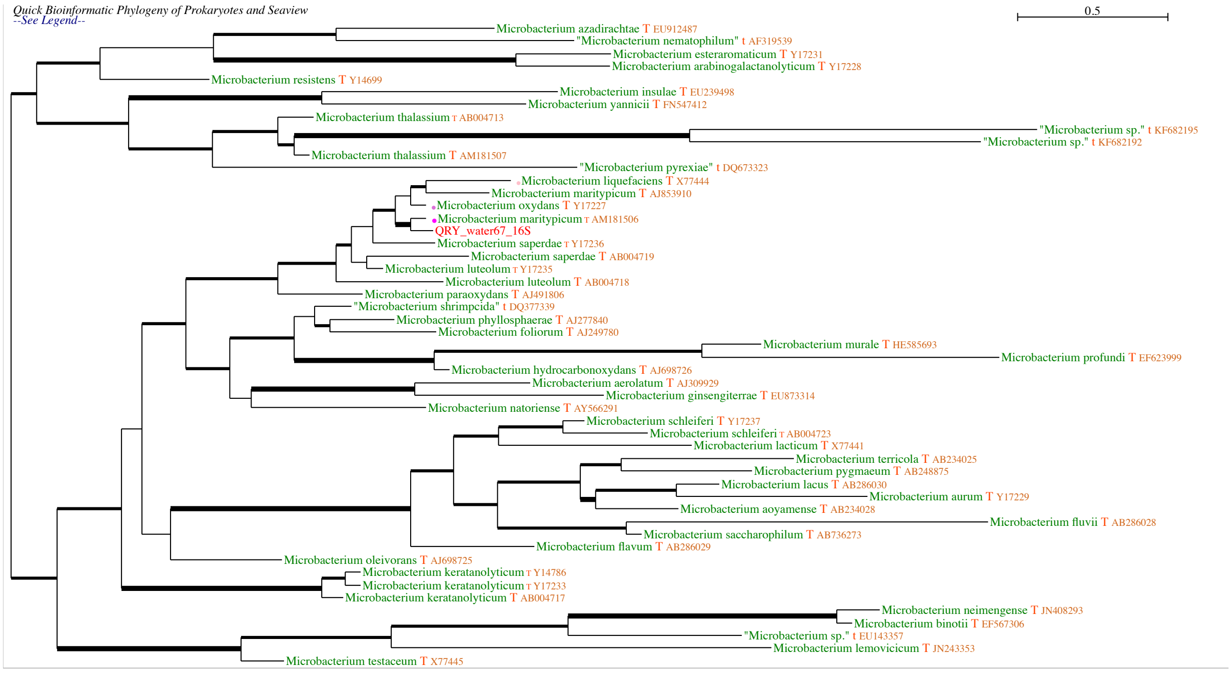

lebibi Quick BioInformatic Phylogeny of Prokaryotes

Summary of the methods and database used during this session

Analysis Extraction of similar sequences for ingroup sequences: Query
There are 44 sequences in your batch
BLAST using the procaryota_SSU-rDNA-16S_TS-stringent database. Trying to extract 50 sequences with expect lower than 0.1
ALIGNMENT using mafft Options : speed oriented
BMGE use for alignment optimization Options :defaults parameters
TREE : phylogenetic tree using fasttree
Evolution model GTR -gamma

RESULTS

Results

Query data

Database: **procaryota_SSU-rDNA-16S_TS-stringent**
Note: The use of the stringent database (all the GenBank sequences for a given species) may lead to errors due to the low quality identification of some sequences, a careful analysis is necessary.
Original name of the query (from fasta) : **QRY_water69_16S.** leBiBI ref : pqpIvwPtL

Sequence composition

Length of Query sequence is : 1278
Nucleotide Composition 307 A - 250 T - 306 C - 415 G - **0 N 0.0 % Gaps % =0.0**
GC%=0.56

Quality of the BLAST analysis

The number of Blast hits is **OK**
All is OK, the best BLAST hit length (**1270**) is =99.3% of the query length

Biodiversity level

All extracted sequences appear to belong to Bacteria and their lowest shared taxon is: Bacteria-Actinobacteria-Actinobacteridae-Actinomycetales-Micrococcineae-Microbacteriaceae-Microbacterium
CAUTION ! One genus only in the extracted sequences. No outgroup *may* be pertinent enough for a good phylogeny. You may need to increase the number of desired sequences or use a *more stringent database*.

Phylogenetic tree analysis

Proximal Cluster ['Microbacterium_oxydans T Y17227', 'Microbacterium_maritypicum T AJ853910', 'Microbacterium_maritypicum T AM181506']

ANALYSIS OF PATRISTIC DISTANCES
Microbacterium_maritypicum T AM181506 is the closest sequence based on patristic distances
On the basis of patristic Distances, the **closest** sequence is: Microbacterium_maritypicum T AM181506 AND is in the proximal cluster
No statistics available for patristic distances between members of the species of the closest sequence
Remark: the patristic distance to Microbacterium_maritypicum T AM181506 is in the 75th percentile of the Microbacterium genus inter-species patristic distances : the sequence may correspond to a member of this genus

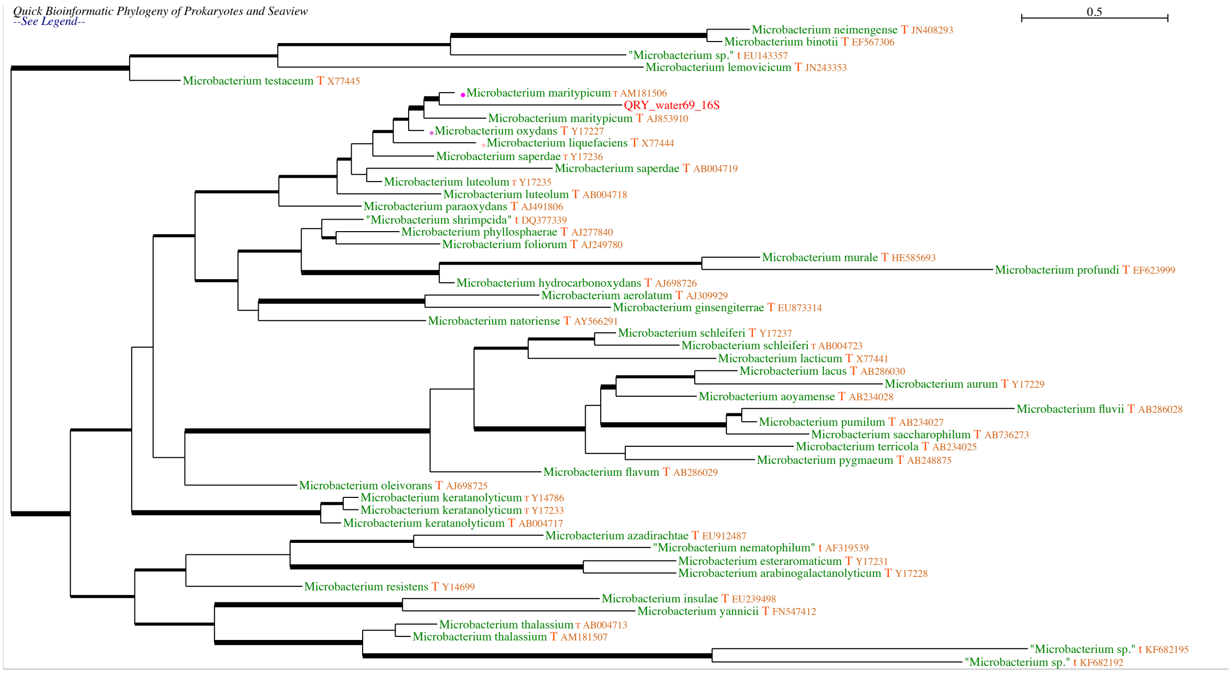

in short | How-to | Cite | Tests sets | Databases | Contact us

lebibi Quick BioInformatic Phylogeny of Prokaryotes

Summary of the methods and database used during this session

Analysis Extraction of similar sequences for ingroup sequences: Query
There are 44 sequences in your batch
BLAST using the procaryota_SSU-rDNA-16S_TS-stringent database. Trying to extract 50 sequences with expect lower than 0.1
ALIGNMENT using mafft Options : speed oriented
BMGE use for alignment optimization Options :defaults parameters
TREE : phylogenetic tree using fasttree
Evolution model GTR -gamma

RESULTS

Results

Query data

Database: **procaryota_SSU-rDNA-16S_TS-stringent**
Note: The use of the stringent database (all the GenBank sequences for a given species) may lead to errors due to the low quality identification of some sequences, a careful analysis is necessary.
Original name of the query (from fasta) : **QRY_water35_16S.** leBiBI ref : pqpIvwPtL

Sequence composition

Length of Query sequence is : 1279
Nucleotide Composition 316 A - 244 T - 306 C - 413 G - **0 N 0.0 % Gaps % =0.0**
GC%=0.56

Quality of the BLAST analysis

The number of Blast hits is **OK**
All is OK, the best BLAST hit length (**1279**) is =100.% of the query length

Biodiversity level

All extracted sequences appear to belong to Bacteria and their lowest shared taxon is: Bacteria-Actinobacteria-Actinobacteridae-Actinomycetales-Micrococcineae-Microbacteriaceae-Microbacterium
CAUTION ! One genus only in the extracted sequences. No outgroup *may* be pertinent enough for a good phylogeny. You may need to increase the number of desired sequences or use a *more stringent database*.

Phylogenetic tree analysis

Proximal Cluster ['Microbacterium_lacticum T X77441']

ANALYSIS OF PATRISTIC DISTANCES
Microbacterium_lacticum T X77441 is the closest sequence based on patristic distances
On the basis of patristic Distances, the **closest** sequence is: Microbacterium_lacticum T X77441 AND is in the proximal cluster
No statistics available for patristic distances between members of the species of the closest sequence
Remark: the patristic distance to Microbacterium_lacticum T X77441 is in the 75th percentile of the Microbacterium genus inter-species patristic distances : the sequence may correspond to a member of this genus

---

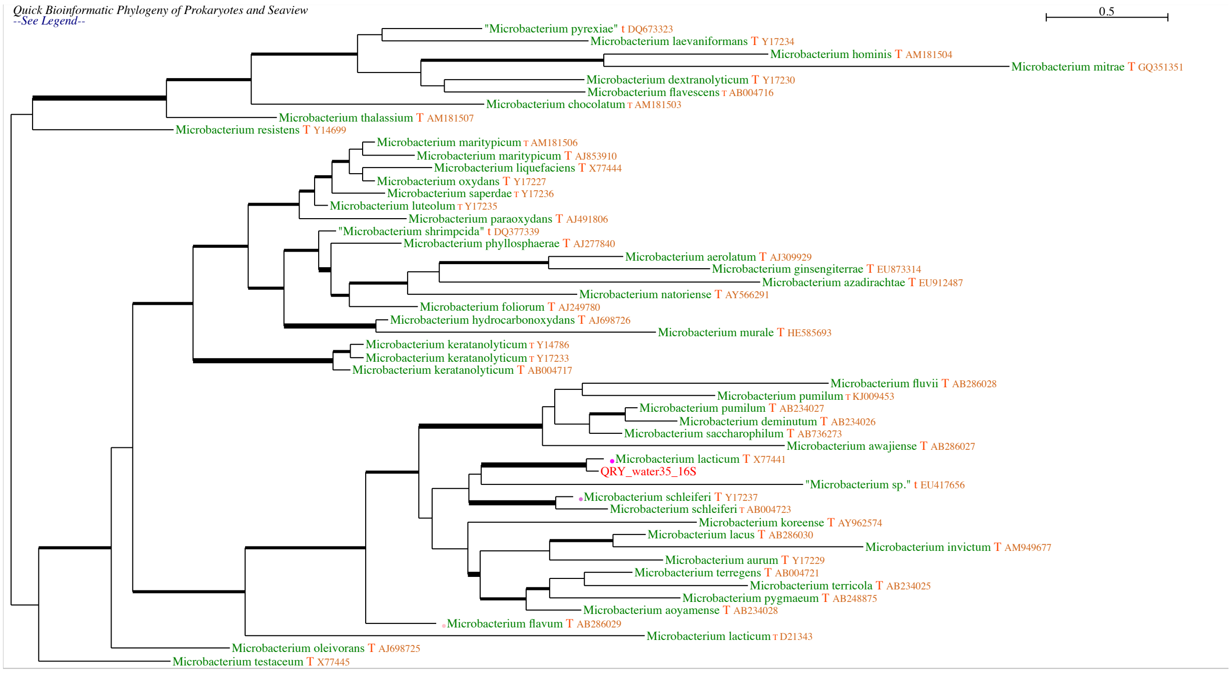

leBIBI QBPP Quick Bioinformatic Phylogeny of Prokaryotes | in short | How-to | Cite | Tests sets | Databases | Contact us | LBBE | prabi

lebibi Quick BioInformatic Phylogeny of Prokaryotes

Summary of the methods and database used during this session

Analysis Extraction of similar sequences for ingroup sequences: Query
There are 44 sequences in your batch
BLAST using the procaryota_SSU-rDNA-16S_TS-stringent database. Trying to extract 50 sequences with expect lower than 0.1
ALIGNMENT using mafft Options : speed oriented
BMGE use for alignment optimization Options :defaults parameters
TREE : phylogenetic tree using fasttree
Evolution model GTR -gamma

RESULTS

Results

Query data

Database: **procaryota_SSU-rDNA-16S_TS-stringent**
Note: The use of the stringent database (all the GenBank sequences for a given species) may lead to errors due to the low quality identification of some sequences, a careful analysis is necessary.
Original name of the query (from fasta) : **QRY_water70_16S.** leBiBI ref : pqpIvwPtL

Sequence composition

Length of Query sequence is : 1284
Nucleotide Composition 317 A - 246 T - 308 C - 413 G - **0 N 0.0 % Gaps % =0.0**
GC%=0.56

Quality of the BLAST analysis

The number of Blast hits is **OK**
All is OK, the best BLAST hit length (**1276**) is =99.3% of the query length

Biodiversity level

All extracted sequences appear to belong to Bacteria and their lowest shared taxon is: Bacteria-Actinobacteria-Actinobacteridae-Actinomycetales-Micrococcineae-Microbacteriaceae-Microbacterium
CAUTION ! One genus only in the extracted sequences. No outgroup *may* be pertinent enough for a good phylogeny. You may need to increase the number of desired sequences or use a *more stringent database*.

Phylogenetic tree analysis

Proximal Cluster ['Microbacterium_lacticum T X77441']

ANALYSIS OF PATRISTIC DISTANCES
Microbacterium_lacticum T X77441 is the closest sequence based on patristic distances
On the basis of patristic Distances, the **closest** sequence is: Microbacterium_lacticum T X77441 AND is in the proximal cluster
No statistics available for patristic distances between members of the species of the closest sequence
Remark: the patristic distance to Microbacterium_lacticum T X77441 is in the 75th percentile of the Microbacterium genus inter-species patristic distances : the sequence may correspond to a member of this genus

---

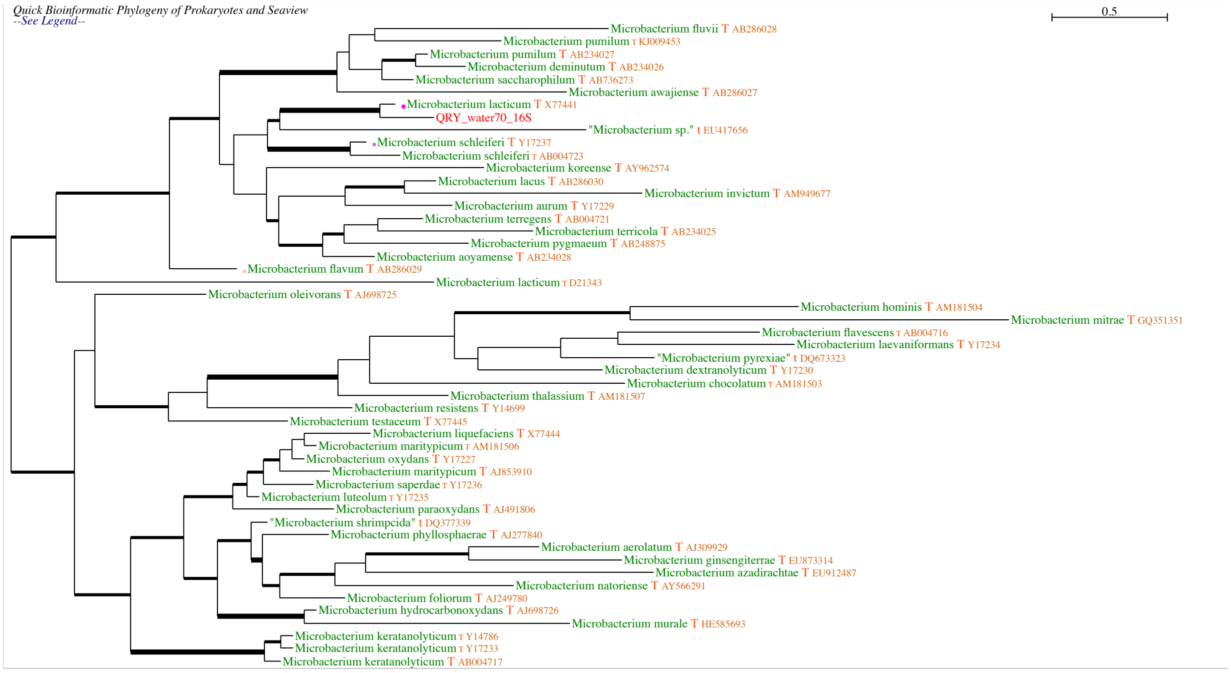

in short | How-to | Cite | Tests sets | Databases | Contact us

lebibi Quick BioInformatic Phylogeny of Prokaryotes

Summary of the methods and database used during this session

Analysis Extraction of similar sequences for ingroup sequences: Query
There are 44 sequences in your batch
BLAST using the procaryota_SSU-rDNA-16S_TS-stringent database. Trying to extract 50 sequences with expect lower than 0.1
ALIGNMENT using mafft Options : speed oriented
BMGE use for alignment optimization Options :defaults parameters
TREE : phylogenetic tree using fasttree
Evolution model GTR -gamma

RESULTS

Results

Query data

Database: **procaryota_SSU-rDNA-16S_TS-stringent**
Note: The use of the stringent database (all the GenBank sequences for a given species) may lead to errors due to the low quality identification of some sequences, a careful analysis is necessary.
Original name of the query (from fasta) : **QRY_water71_16S.** leBiBI ref : pqpIvwPtL

Sequence composition

Length of Query sequence is : 1269
Nucleotide Composition 305 A - 248 T - 299 C - 417 G - **0 N 0.0 % Gaps % =0.0**
GC%=0.56

Quality of the BLAST analysis

The number of Blast hits is **OK**
All is OK, the best BLAST hit length (**1270**) is =100.% of the query length

Biodiversity level

All extracted sequences appear to belong to Bacteria and their lowest shared taxon is: Bacteria-Actinobacteria-Actinobacteridae-Actinomycetales-Micrococcineae-Microbacteriaceae-Microbacterium
CAUTION ! One genus only in the extracted sequences. No outgroup *may* be pertinent enough for a good phylogeny. You may need to increase the number of desired sequences or use a *more stringent database*.

Phylogenetic tree analysis

Proximal Cluster ['Microbacterium_oxydans T Y17227', 'Microbacterium_maritypicum T AJ853910', 'Microbacterium_maritypicum T AM181506']

ANALYSIS OF PATRISTIC DISTANCES
Microbacterium_maritypicum T AM181506 is the closest sequence based on patristic distances
On the basis of patristic Distances, the **closest** sequence is: Microbacterium_maritypicum T AM181506 AND is in the proximal cluster
No statistics available for patristic distances between members of the species of the closest sequence
Remark: the patristic distance to Microbacterium_maritypicum T AM181506 is in the 75th percentile of the Microbacterium genus inter-species patristic distances : the sequence may correspond to a member of this genus

---

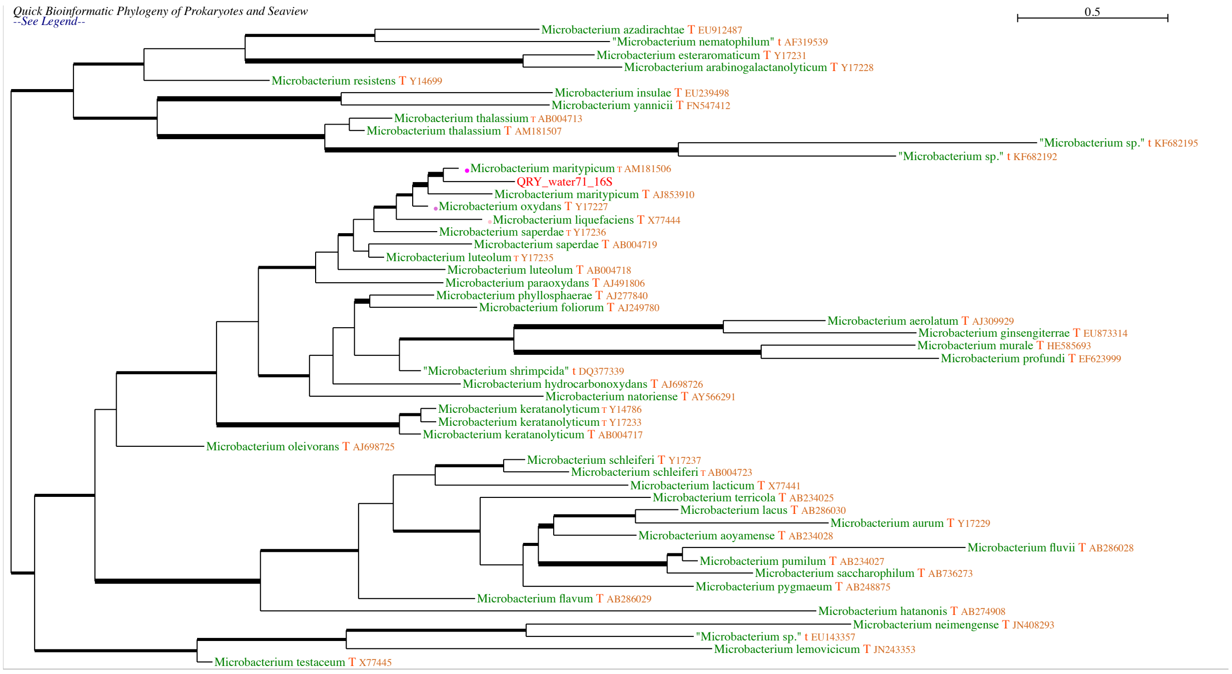

QBPP Quick Bioinformatic Phylogeny of Prokaryotes | in short | How-to | Cite | Tests sets | Databases | Contact us | LBBE | prabi

lebibi Quick BioInformatic Phylogeny of Prokaryotes

Summary of the methods and database used during this session

Analysis Extraction of similar sequences for ingroup sequences: Query
There are 44 sequences in your batch
BLAST using the procaryota_SSU-rDNA-16S_TS-stringent database. Trying to extract 50 sequences with expect lower than 0.1
ALIGNMENT using mafft Options : speed oriented
BMGE use for alignment optimization Options :defaults parameters
TREE : phylogenetic tree using fasttree
Evolution model GTR -gamma

RESULTS

Results

Query data

Database: **procaryota_SSU-rDNA-16S_TS-stringent**
Note: The use of the stringent database (all the GenBank sequences for a given species) may lead to errors due to the low quality identification of some sequences, a careful analysis is necessary.
Original name of the query (from fasta) : **QRY_water72_16S.** leBiBI ref : pqpIvwPtL

Sequence composition

Length of Query sequence is : 1277
Nucleotide Composition 312 A - 246 T - 309 C - 410 G - **0 N 0.0 %** **Gaps % =0.0**
GC%=0.56

Quality of the BLAST analysis

The number of Blast hits is **OK**
All is OK, the best BLAST hit length (**1270**) is =99.4% of the query length

Biodiversity level

All extracted sequences appear to belong to Bacteria and their lowest shared taxon is: Bacteria-Actinobacteria-Actinobacteridae-Actinomycetales-Micrococcineae-Microbacteriaceae-Microbacterium
CAUTION ! One genus only in the extracted sequences. No outgroup *may* be pertinent enough for a good phylogeny. You may need to increase the number of desired sequences or use a *more stringent database*.

Phylogenetic tree analysis

Proximal Cluster ['Microbacterium_sp. ? T EU417656', 'Microbacterium_lacticum T X77441']

ANALYSIS OF PATRISTIC DISTANCES
Microbacterium_lacticum T X77441 is the closest sequence based on patristic distances
On the basis of patristic Distances, the **closest** sequence is: Microbacterium_lacticum T X77441 AND is in the proximal cluster
No statistics available for patristic distances between members of the species of the closest sequence
Remark: the patristic distance to Microbacterium_lacticum T X77441 is in the 75th percentile of the Microbacterium genus inter-species patristic distances : the sequence may correspond to a member of this genus

---

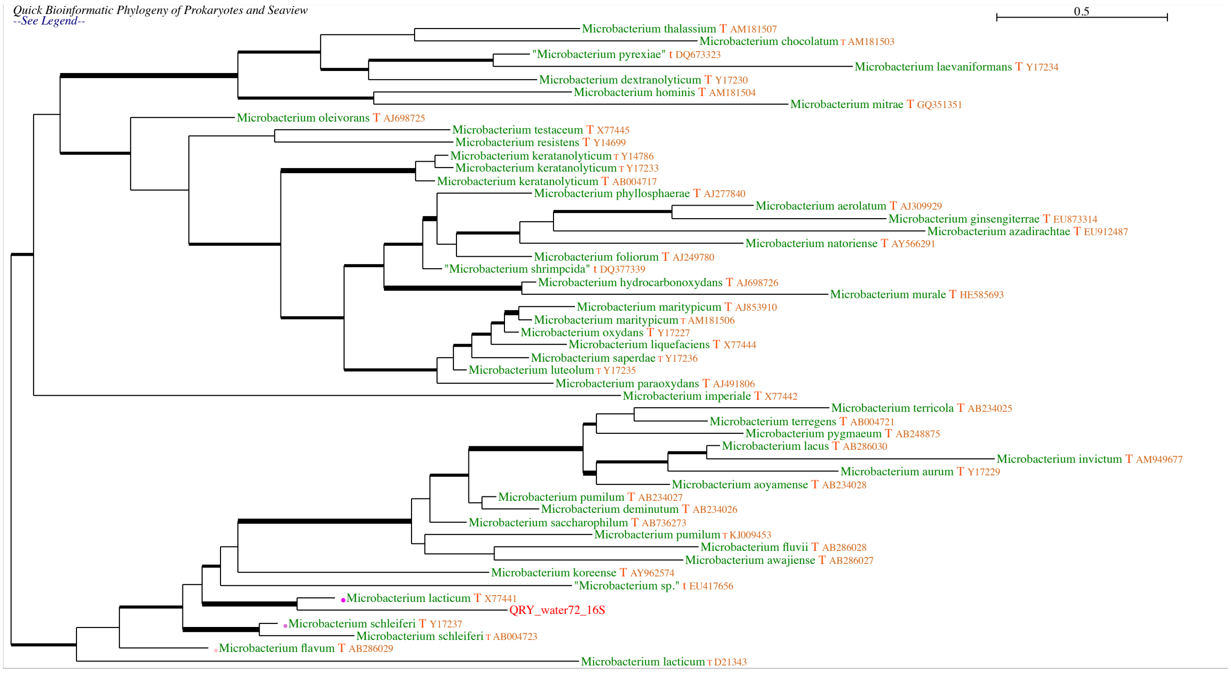

in short | How-to | Cite | Tests sets | Databases | Contact us

lebibi Quick BioInformatic Phylogeny of Prokaryotes

Summary of the methods and database used during this session

Analysis Extraction of similar sequences for ingroup sequences: Query
There are 44 sequences in your batch
BLAST using the procaryota_SSU-rDNA-16S_TS-stringent database. Trying to extract 50 sequences with expect lower than 0.1
ALIGNMENT using mafft Options : speed oriented
BMGE use for alignment optimization Options :defaults parameters
TREE : phylogenetic tree using fasttree
Evolution model GTR -gamma

RESULTS

Results

Query data

Database: **procaryota_SSU-rDNA-16S_TS-stringent**
Note: The use of the stringent database (all the GenBank sequences for a given species) may lead to errors due to the low quality identification of some sequences, a careful analysis is necessary.
Original name of the query (from fasta) : **QRY_water73_16S.** leBiBI ref : pqpIvwPtL

Sequence composition

Length of Query sequence is : 1268
Nucleotide Composition 314 A - 244 T - 302 C - 408 G - **0 N 0.0 %** **Gaps % =0.0**
GC%=0.55

Quality of the BLAST analysis

The number of Blast hits is **OK**
All is OK, the best BLAST hit length (**1266**) is =99.8% of the query length

Biodiversity level

All extracted sequences appear to belong to Bacteria and their lowest shared taxon is: Bacteria-Actinobacteria-Actinobacteridae-Actinomycetales-Micrococcineae-Microbacteriaceae
More than one genus in the extracted sequences. An outgroup will be present in the tree.

Phylogenetic tree analysis

Proximal Cluster ['Microbacterium_aurum T Y17229']

ANALYSIS OF PATRISTIC DISTANCES
Microbacterium_aurum T Y17229 is the closest sequence based on patristic distances
On the basis of patristic Distances, the **closest** sequence is: Microbacterium_aurum T Y17229 AND is in the proximal cluster
No statistics available for patristic distances between members of the species of the closest sequence
Remark: the patristic distance to Microbacterium_aurum T Y17229 is in the 75th percentile of the Microbacterium genus inter-species patristic distances : the sequence may correspond to a member of this genus

---

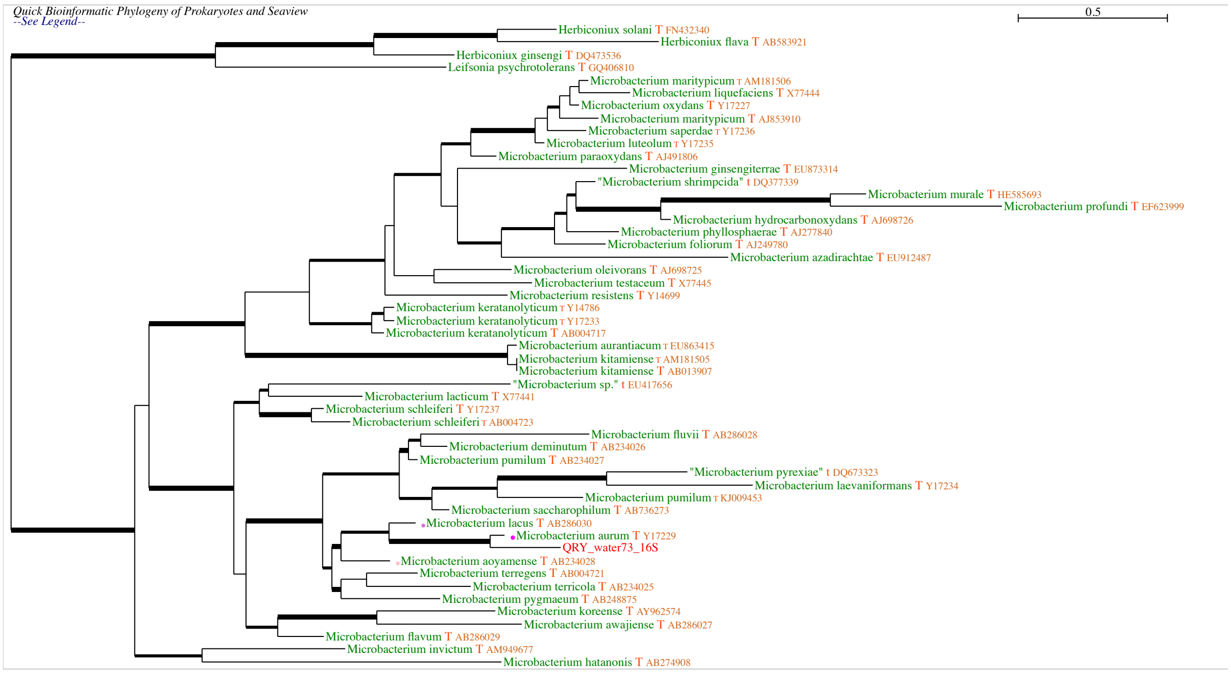

lebibi Quick BioInformatic Phylogeny of Prokaryotes

Summary of the methods and database used during this session

Analysis Extraction of similar sequences for ingroup sequences: Query
There are 44 sequences in your batch
BLAST using the procaryota_SSU-rDNA-16S_TS-stringent database. Trying to extract 50 sequences with expect lower than 0.1
ALIGNMENT using mafft Options : speed oriented
BMGE use for alignment optimization Options :defaults parameters
TREE : phylogenetic tree using fasttree
Evolution model GTR -gamma

RESULTS

Results

Query data

Database: **procaryota_SSU-rDNA-16S_TS-stringent**
Note: The use of the stringent database (all the GenBank sequences for a given species) may lead to errors due to the low quality identification of some sequences, a careful analysis is necessary.
Original name of the query (from fasta) : **QRY_water74_16S.** leBiBI ref : pqpIvwPtL

Sequence composition

Length of Query sequence is : 1269
Nucleotide Composition 316 A - 245 T - 302 C - 406 G - **0 N 0.0 % Gaps % =0.0**
GC%=0.55

Quality of the BLAST analysis

The number of Blast hits is **OK**
All is OK, the best BLAST hit length (**1269**) is =100.% of the query length

Biodiversity level

All extracted sequences appear to belong to Bacteria and their lowest shared taxon is: Bacteria-Actinobacteria-Actinobacteridae-Actinomycetales-Micrococcineae-Microbacteriaceae
More than one genus in the extracted sequences. An outgroup will be present in the tree.

Phylogenetic tree analysis

Proximal Cluster ['Microbacterium_hatanonis T AB274908', 'Microbacterium_aurum T Y17229']

ANALYSIS OF PATRISTIC DISTANCES
Microbacterium_aurum T Y17229 is the closest sequence based on patristic distances
On the basis of patristic Distances, the **closest** sequence is: Microbacterium_aurum T Y17229 AND is in the proximal cluster
No statistics available for patristic distances between members of the species of the closest sequence
Remark: the patristic distance to Microbacterium_aurum T Y17229 is in the 75th percentile of the Microbacterium genus inter-species patristic distances : the sequence may correspond to a member of this genus

---

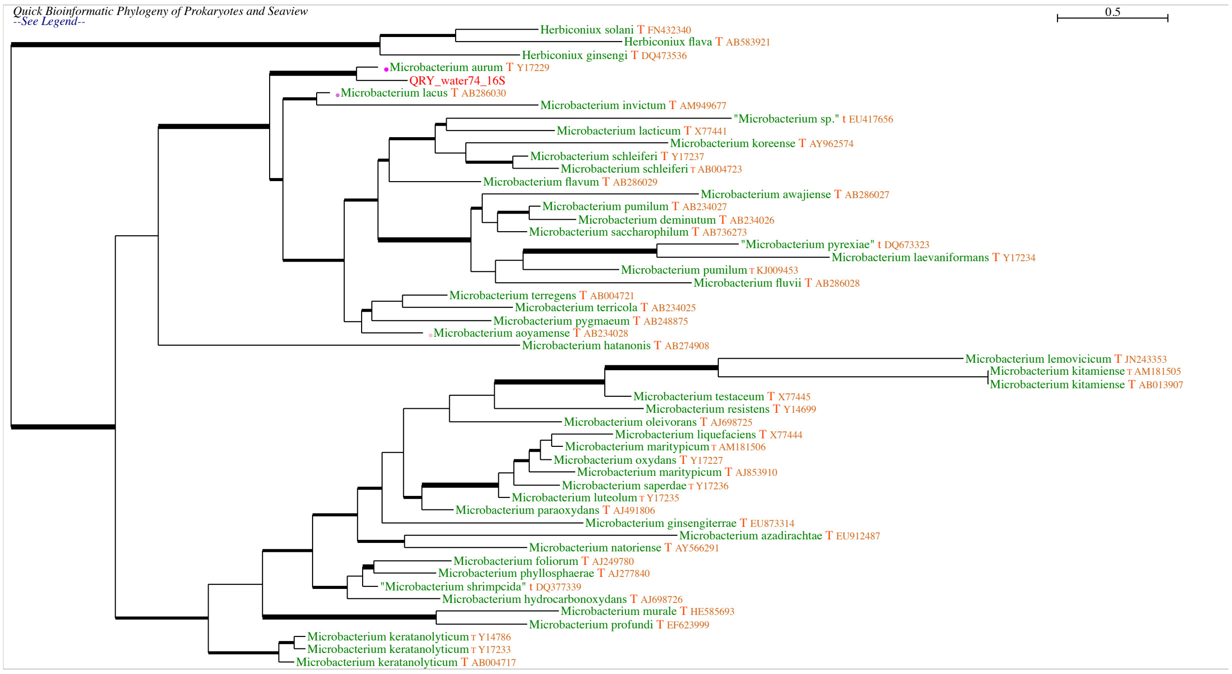

in short | How-to | Cite | Tests sets | Databases | Contact us

lebibi Quick BioInformatic Phylogeny of Prokaryotes

Summary of the methods and database used during this session

Analysis Extraction of similar sequences for ingroup sequences: Query
There are 44 sequences in your batch
BLAST using the procaryota_SSU-rDNA-16S_TS-stringent database. Trying to extract 50 sequences with expect lower than 0.1
ALIGNMENT using mafft Options : speed oriented
BMGE use for alignment optimization Options :defaults parameters
TREE : phylogenetic tree using fasttree
Evolution model GTR -gamma

RESULTS

Results

Query data

Database: **procaryota_SSU-rDNA-16S_TS-stringent**
Note: The use of the stringent database (all the GenBank sequences for a given species) may lead to errors due to the low quality identification of some sequences, a careful analysis is necessary.
Original name of the query (from fasta) : **QRY_water75_16S.** leBiBI ref : pqpIvwPtL

Sequence composition

Length of Query sequence is : 1268
Nucleotide Composition 303 A - 248 T - 300 C - 417 G - **0 N 0.0 % Gaps % =0.0**
GC%=0.56

Quality of the BLAST analysis

The number of Blast hits is **OK**
All is OK, the best BLAST hit length (**1266**) is =99.8% of the query length

Biodiversity level

All extracted sequences appear to belong to Bacteria and their lowest shared taxon is: Bacteria-Actinobacteria-Actinobacteridae-Actinomycetales-Micrococcineae-Microbacteriaceae-Microbacterium
CAUTION ! One genus only in the extracted sequences. No outgroup *may* be pertinent enough for a good phylogeny. You may need to increase the number of desired sequences or use a *more stringent database*.

Phylogenetic tree analysis

Proximal Cluster ['Microbacterium_maritypicum T AM181506']

ANALYSIS OF PATRISTIC DISTANCES
Microbacterium_maritypicum T AM181506 is the closest sequence based on patristic distances
On the basis of patristic Distances, the **closest** sequence is: Microbacterium_maritypicum T AM181506 AND is in the proximal cluster
No statistics available for patristic distances between members of the species of the closest sequence
Remark: the patristic distance to Microbacterium_maritypicum T AM181506 is in the 75th percentile of the Microbacterium genus inter-species patristic distances : the sequence may correspond to a member of this genus

---

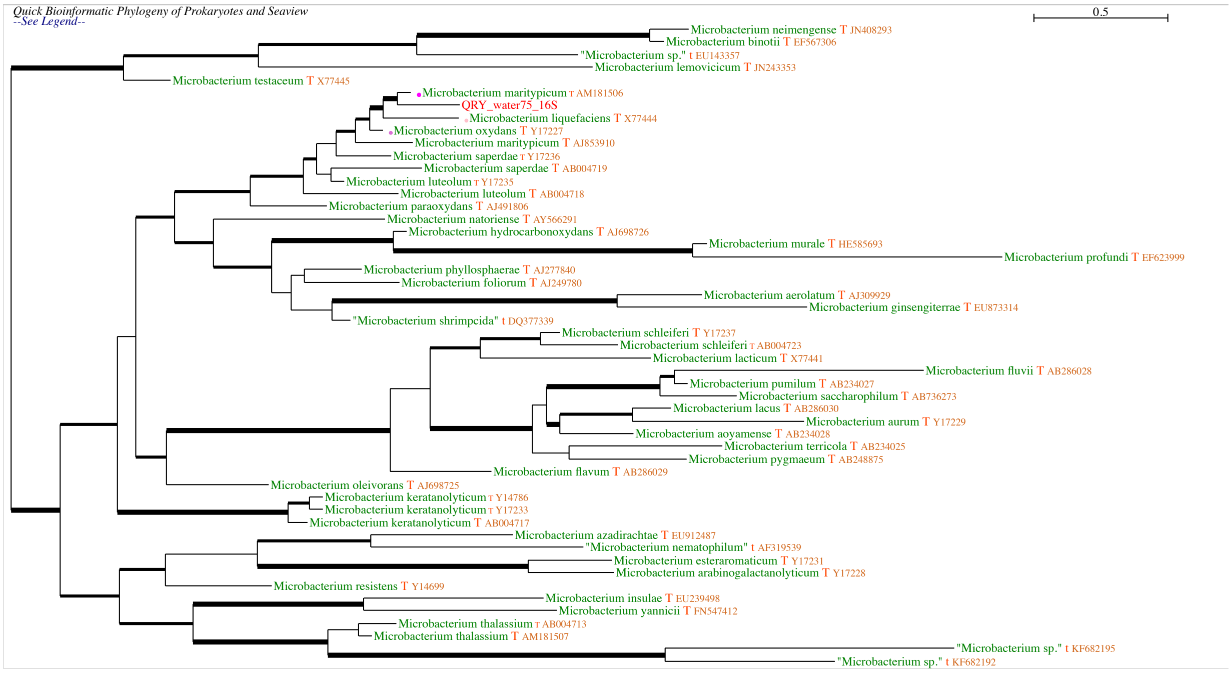

lebibi Quick BioInformatic Phylogeny of Prokaryotes

Summary of the methods and database used during this session

Analysis Extraction of similar sequences for ingroup sequences: Query
There are 44 sequences in your batch
BLAST using the procaryota_SSU-rDNA-16S_TS-stringent database. Trying to extract 50 sequences with expect lower than 0.1
ALIGNMENT using mafft Options : speed oriented
BMGE use for alignment optimization Options :defaults parameters
TREE : phylogenetic tree using fasttree
Evolution model GTR -gamma

RESULTS

Results

Query data

Database: **procaryota_SSU-rDNA-16S_TS-stringent**
Note: The use of the stringent database (all the GenBank sequences for a given species) may lead to errors due to the low quality identification of some sequences, a careful analysis is necessary.
Original name of the query (from fasta) : **QRY_water76_16S.** leBiBI ref : pqpIvwPtL

Sequence composition

Length of Query sequence is : 1270
Nucleotide Composition 313 A - 245 T - 303 C - 409 G - **0 N 0.0 %** **Gaps % =0.0**
GC%=0.56

Quality of the BLAST analysis

The number of Blast hits is **OK**
All is OK, the best BLAST hit length (**1266**) is =99.6% of the query length

Biodiversity level

All extracted sequences appear to belong to Bacteria and their lowest shared taxon is: Bacteria-Actinobacteria-Actinobacteridae-Actinomycetales-Micrococcineae-Microbacteriaceae-Microbacterium
CAUTION ! One genus only in the extracted sequences. No outgroup *may* be pertinent enough for a good phylogeny. You may need to increase the number of desired sequences or use a *more stringent database*.

Phylogenetic tree analysis

Proximal Cluster ['Microbacterium_lacticum T X77441']

ANALYSIS OF PATRISTIC DISTANCES
Microbacterium_lacticum T X77441 is the closest sequence based on patristic distances
On the basis of patristic Distances, the **closest** sequence is: Microbacterium_lacticum T X77441 AND is in the proximal cluster
No statistics available for patristic distances between members of the species of the closest sequence
Remark: the patristic distance to Microbacterium_lacticum T X77441 is in the 75th percentile of the Microbacterium genus inter-species patristic distances : the sequence may correspond to a member of this genus

---

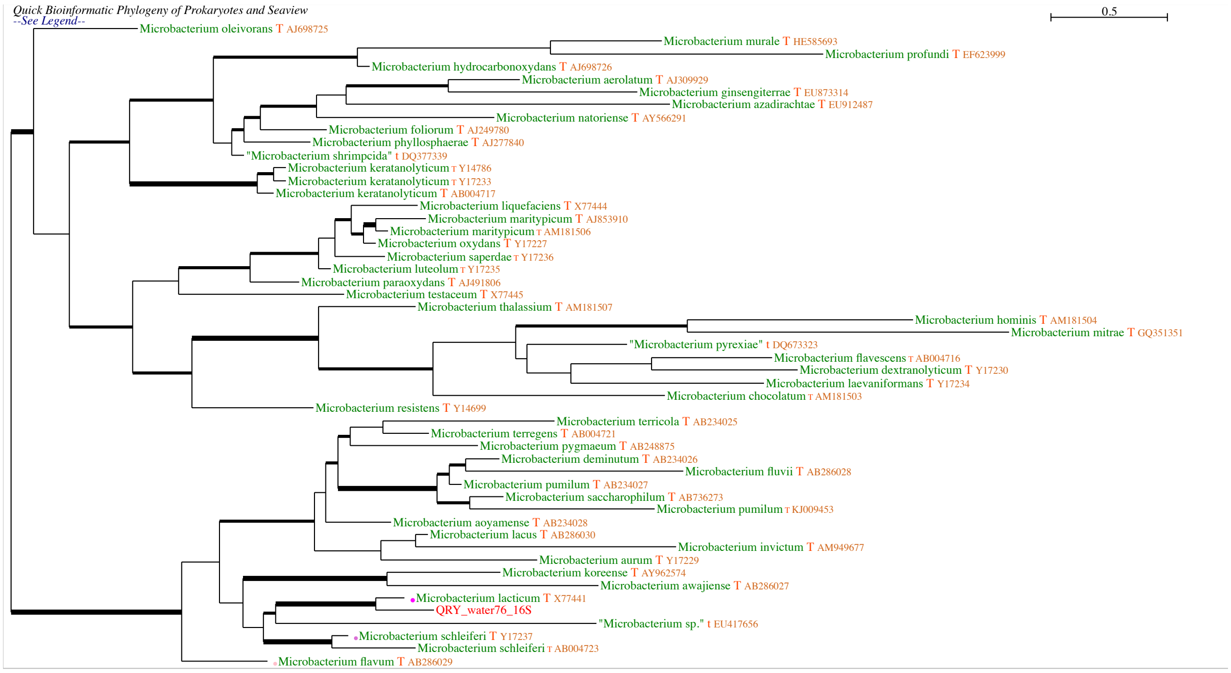

lebibi Quick BioInformatic Phylogeny of Prokaryotes

Summary of the methods and database used during this session

Analysis Extraction of similar sequences for ingroup sequences: Query
There are 44 sequences in your batch
BLAST using the procaryota_SSU-rDNA-16S_TS-stringent database. Trying to extract 50 sequences with expect lower than 0.1
ALIGNMENT using mafft Options : speed oriented
BMGE use for alignment optimization Options :defaults parameters
TREE : phylogenetic tree using fasttree
Evolution model GTR -gamma

RESULTS

Results

Query data

Database: **procaryota_SSU-rDNA-16S_TS-stringent**
Note: The use of the stringent database (all the GenBank sequences for a given species) may lead to errors due to the low quality identification of some sequences, a careful analysis is necessary.
Original name of the query (from fasta) : **QRY_water77_16S.** leBiBI ref : pqpIvwPtL

Sequence composition

Length of Query sequence is : 1230
Nucleotide Composition 294 A - 244 T - 297 C - 395 G - **0 N 0.0 % Gaps % =0.0**
GC%=0.56

Quality of the BLAST analysis

The number of Blast hits is **OK**
All is OK, the best BLAST hit length (**1230**) is =100.% of the query length

Biodiversity level

All extracted sequences appear to belong to Bacteria and their lowest shared taxon is: Bacteria-Proteobacteria-Alphaproteobacteria-Rhizobiales-Methylobacteriaceae-Methylobacterium
CAUTION ! One genus only in the extracted sequences. No outgroup *may* be pertinent enough for a good phylogeny. You may need to increase the number of desired sequences or use a *more stringent database*.

Phylogenetic tree analysis

Proximal Cluster ['Methylobacterium_thiocyanatum T U58018', 'Methylobacterium_rhodesianum T AB175643', 'Methylobacterium_rhodesianum T AB175642']

ANALYSIS OF PATRISTIC DISTANCES
Methylobacterium_rhodesianum T AB175643 is the closest sequence based on patristic distances
On the basis of patristic Distances, the **closest** sequence is: Methylobacterium_rhodesianum T AB175643 AND is in the proximal cluster
No statistics available for patristic distances between members of the species of the closest sequence
Remark: the patristic distance to Methylobacterium_rhodesianum T AB175643 is in the 75th percentile of the Methylobacterium genus inter-species patristic distances : the sequence may correspond to a member of this genus
The closest sequence based on patristic distances (Methylobacterium_rhodesianum T AB175643) is NOT the FIRST BLAST hit.

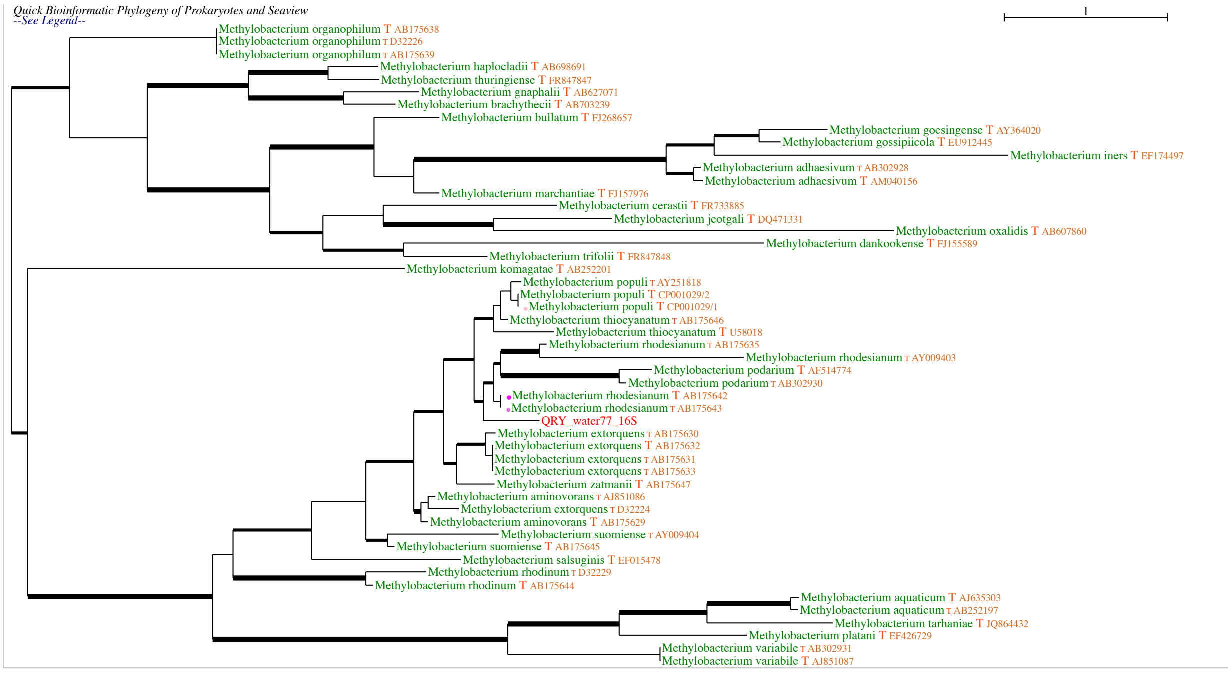

in short | How-to | Cite | Tests sets | Databases | Contact us

lebibi Quick BioInformatic Phylogeny of Prokaryotes

Summary of the methods and database used during this session

Analysis Extraction of similar sequences for ingroup sequences: Query
There are 44 sequences in your batch
BLAST using the procaryota_SSU-rDNA-16S_TS-stringent database. Trying to extract 50 sequences with expect lower than 0.1
ALIGNMENT using mafft Options : speed oriented
BMGE use for alignment optimization Options :defaults parameters
TREE : phylogenetic tree using fasttree
Evolution model GTR -gamma

RESULTS

Results

Query data

Database: **procaryota_SSU-rDNA-16S_TS-stringent**

Note: The use of the stringent database (all the GenBank sequences for a given species) may lead to errors due to the low quality identification of some sequences, a careful analysis is necessary.

Original name of the query (from fasta) : **QRY_water78_16S.** leBiBI ref : pqpIvwPtL

Sequence composition

Length of Query sequence is : 1270

Nucleotide Composition 313 A - 242 T - 304 C - 411 G - **0 N 0.0 %** **Gaps % =0.0**

GC%=0.56

Quality of the BLAST analysis

The number of Blast hits is **OK**

All is OK, the best BLAST hit length (**1270**) is =100.% of the query length

Biodiversity level

All extracted sequences appear to belong to Bacteria and their lowest shared taxon is: Bacteria-Actinobacteria-Actinobacteridae-Actinomycetales-Micrococcineae-Microbacteriaceae-Microbacterium

CAUTION ! One genus only in the extracted sequences. No outgroup *may* be pertinent enough for a good phylogeny. You may need to increase the number of desired sequences or use a *more stringent database*.

Phylogenetic tree analysis

Proximal Cluster ['Microbacterium_lacticum T X77441']

ANALYSIS OF PATRISTIC DISTANCES

Microbacterium_lacticum T X77441 is the closest sequence based on patristic distances

On the basis of patristic Distances, the **closest** sequence is: Microbacterium_lacticum T X77441 AND is in the proximal cluster

No statistics available for patristic distances between members of the species of the closest sequence

Remark: the patristic distance to Microbacterium_lacticum T X77441 is in the 75th percentile of the Microbacterium genus inter-species patristic distances : the sequence may correspond to a member of this genus

---

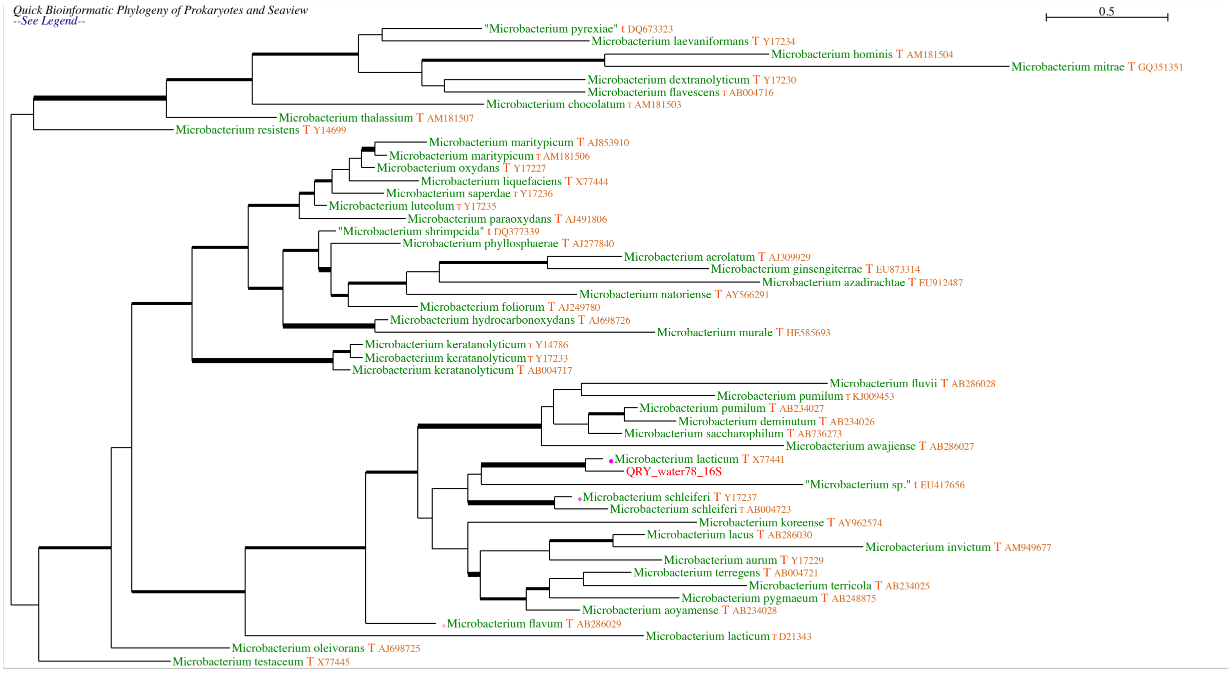

in short | How-to | Cite | Tests sets | Databases | Contact us

lebibi Quick BioInformatic Phylogeny of Prokaryotes

Summary of the methods and database used during this session

Analysis Extraction of similar sequences for ingroup sequences: Query
There are 44 sequences in your batch
BLAST using the procaryota_SSU-rDNA-16S_TS-stringent database. Trying to extract 50 sequences with expect lower than 0.1
ALIGNMENT using mafft Options : speed oriented
BMGE use for alignment optimization Options :defaults parameters
TREE : phylogenetic tree using fasttree
Evolution model GTR -gamma

RESULTS

Results

Query data

Database: **procaryota_SSU-rDNA-16S_TS-stringent**
Note: The use of the stringent database (all the GenBank sequences for a given species) may lead to errors due to the low quality identification of some sequences, a careful analysis is necessary.
Original name of the query (from fasta) : **QRY_water80_16S.** leBiBI ref : pqpIvwPtL

Sequence composition

Length of Query sequence is : 1269
Nucleotide Composition 316 A - 241 T - 303 C - 409 G - **0 N 0.0 % Gaps % =0.0**
GC%=0.56

Quality of the BLAST analysis

The number of Blast hits is **OK**
All is OK, the best BLAST hit length (**1265**) is =99.6% of the query length

Biodiversity level

All extracted sequences appear to belong to Bacteria and their lowest shared taxon is: Bacteria-Actinobacteria-Actinobacteridae-Actinomycetales-Micrococcineae-Microbacteriaceae-Microbacterium
CAUTION ! One genus only in the extracted sequences. No outgroup *may* be pertinent enough for a good phylogeny. You may need to increase the number of desired sequences or use a *more stringent database*.

Phylogenetic tree analysis

Proximal Cluster ['Microbacterium_mitrae T GQ351351', 'Microbacterium_invictum T AM949677']

ANALYSIS OF PATRISTIC DISTANCES
Absence of the closest sequence based on patristic distances Microbacterium_terricola T AB234025 from the proximal cluster.
Look at the tree and branch support
On the basis of patristic Distances, the **closest TS** sequence is: Microbacterium_terricola T AB234025 AND is NOT in the proximal cluster
The closest sequence has not the same species name as the closest TS
No statistics available for patristic distances between members of the species of the closest sequence
Remark: the patristic distance to Microbacterium_terricola T AB234025 is in the 75th percentile of the Microbacterium genus inter-species patristic distances : the sequence may correspond to a member of this genus
The closest sequence based on patristic distances (Microbacterium_terricola T AB234025) is NOT the FIRST BLAST hit.

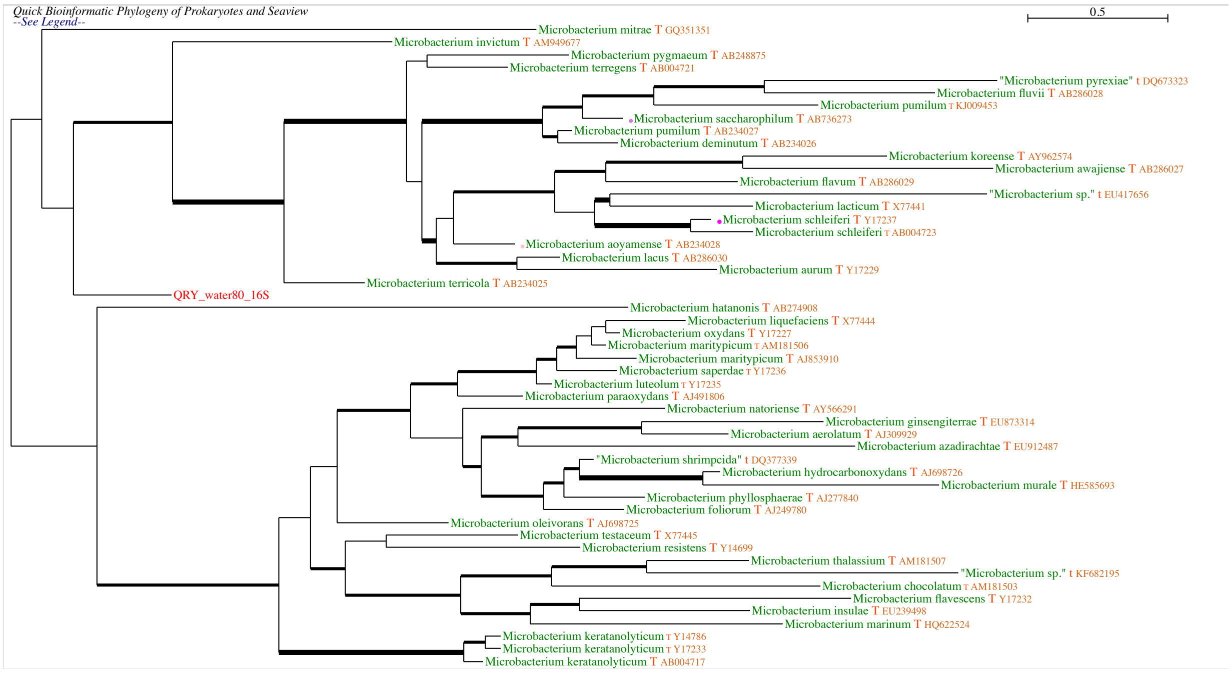

lebibi Quick BioInformatic Phylogeny of Prokaryotes

Summary of the methods and database used during this session

Analysis Extraction of similar sequences for ingroup sequences: Query
There are 44 sequences in your batch
BLAST using the procaryota_SSU-rDNA-16S_TS-stringent database. Trying to extract 50 sequences with expect lower than 0.1
ALIGNMENT using mafft Options : speed oriented
BMGE use for alignment optimization Options :defaults parameters
TREE : phylogenetic tree using fasttree
Evolution model GTR -gamma

RESULTS

Results

Query data

Database: **procaryota_SSU-rDNA-16S_TS-stringent**
Note: The use of the stringent database (all the GenBank sequences for a given species) may lead to errors due to the low quality identification of some sequences, a careful analysis is necessary.
Original name of the query (from fasta) : **QRY_water37_16S.** leBiBI ref : pqpIvwPtL

Sequence composition

Length of Query sequence is : 1279
Nucleotide Composition 317 A - 244 T - 306 C - 412 G - **0 N 0.0 % Gaps % =0.0**
GC%=0.56

Quality of the BLAST analysis

The number of Blast hits is **OK**
All is OK, the best BLAST hit length (**1279**) is =100.% of the query length

Biodiversity level

All extracted sequences appear to belong to Bacteria and their lowest shared taxon is: Bacteria-Actinobacteria-Actinobacteridae-Actinomycetales-Micrococcineae-Microbacteriaceae-Microbacterium
CAUTION ! One genus only in the extracted sequences. No outgroup *may* be pertinent enough for a good phylogeny. You may need to increase the number of desired sequences or use a *more stringent database*.

Phylogenetic tree analysis

Proximal Cluster ['Microbacterium_lacticum T X77441']

ANALYSIS OF PATRISTIC DISTANCES
Microbacterium_lacticum T X77441 is the closest sequence based on patristic distances
On the basis of patristic Distances, the **closest** sequence is: Microbacterium_lacticum T X77441 AND is in the proximal cluster
No statistics available for patristic distances between members of the species of the closest sequence
Remark: the patristic distance to Microbacterium_lacticum T X77441 is in the 75th percentile of the Microbacterium genus inter-species patristic distances : the sequence may correspond to a member of this genus

---

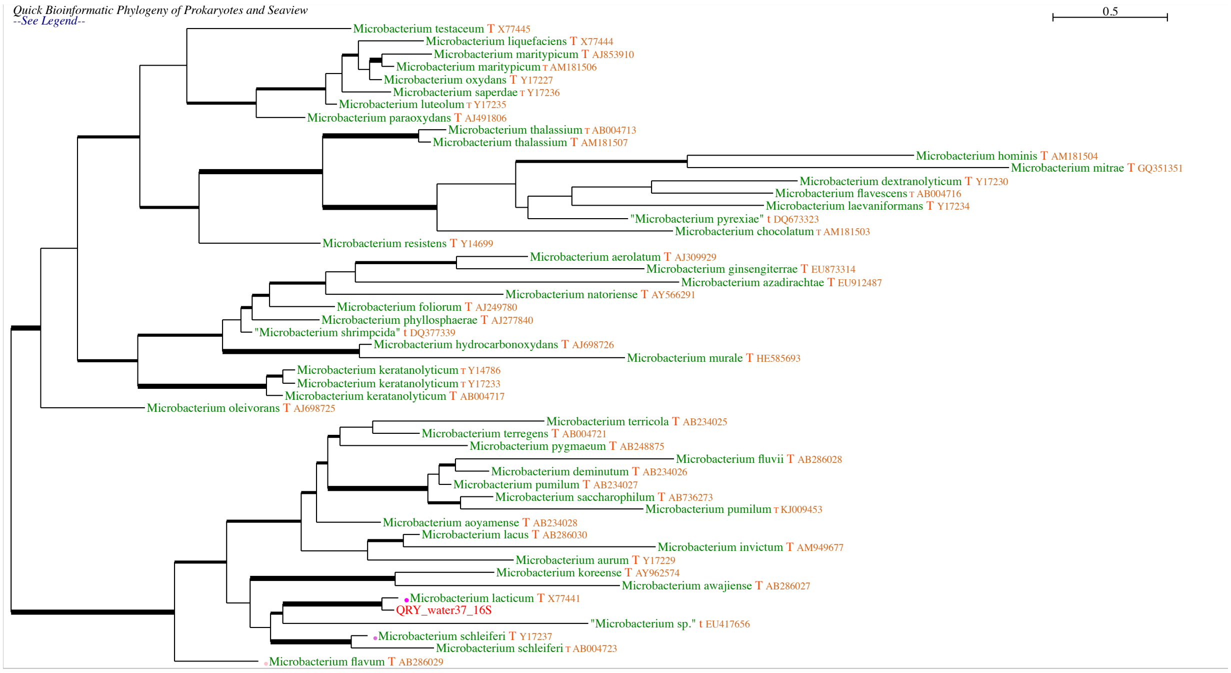

in short | How-to | Cite | Tests sets | Databases | Contact us

lebibi Quick BioInformatic Phylogeny of Prokaryotes

Summary of the methods and database used during this session

Analysis Extraction of similar sequences for ingroup sequences: Query
There are 44 sequences in your batch
BLAST using the procaryota_SSU-rDNA-16S_TS-stringent database. Trying to extract 50 sequences with expect lower than 0.1
ALIGNMENT using mafft Options : speed oriented
BMGE use for alignment optimization Options :defaults parameters
TREE : phylogenetic tree using fasttree
Evolution model GTR -gamma

RESULTS

Results

Query data

Database: **procaryota_SSU-rDNA-16S_TS-stringent**
Note: The use of the stringent database (all the GenBank sequences for a given species) may lead to errors due to the low quality identification of some sequences, a careful analysis is necessary.
Original name of the query (from fasta) : **QRY_water81_16S.** leBiBI ref : pqpIvwPtL

Sequence composition

Length of Query sequence is : 1233
Nucleotide Composition 292 A - 244 T - 296 C - 401 G - **0 N 0.0 % Gaps % =0.0**
GC%=0.56

Quality of the BLAST analysis

The number of Blast hits is **OK**
All is OK, the best BLAST hit length (**1233**) is =100.% of the query length

Biodiversity level

All extracted sequences appear to belong to Bacteria and their lowest shared taxon is: Bacteria-Proteobacteria-Alphaproteobacteria-Rhizobiales-Methylobacteriaceae-Methylobacterium
CAUTION ! One genus only in the extracted sequences. No outgroup *may* be pertinent enough for a good phylogeny. You may need to increase the number of desired sequences or use a *more stringent database*.

Phylogenetic tree analysis

Proximal Cluster ['Methylobacterium_thiocyanatum T U58018', 'Methylobacterium_rhodesianum T AB175643', 'Methylobacterium_rhodesianum T AB175642']

ANALYSIS OF PATRISTIC DISTANCES
Methylobacterium_rhodesianum T AB175643 is the closest sequence based on patristic distances
On the basis of patristic Distances, the **closest** sequence is: Methylobacterium_rhodesianum T AB175643 AND is in the proximal cluster
No statistics available for patristic distances between members of the species of the closest sequence
Remark: the patristic distance to Methylobacterium_rhodesianum T AB175643 is in the 75th percentile of the Methylobacterium genus inter-species patristic distances : the sequence may correspond to a member of this genus
The closest sequence based on patristic distances (Methylobacterium_rhodesianum T AB175643) is NOT the FIRST BLAST hit.

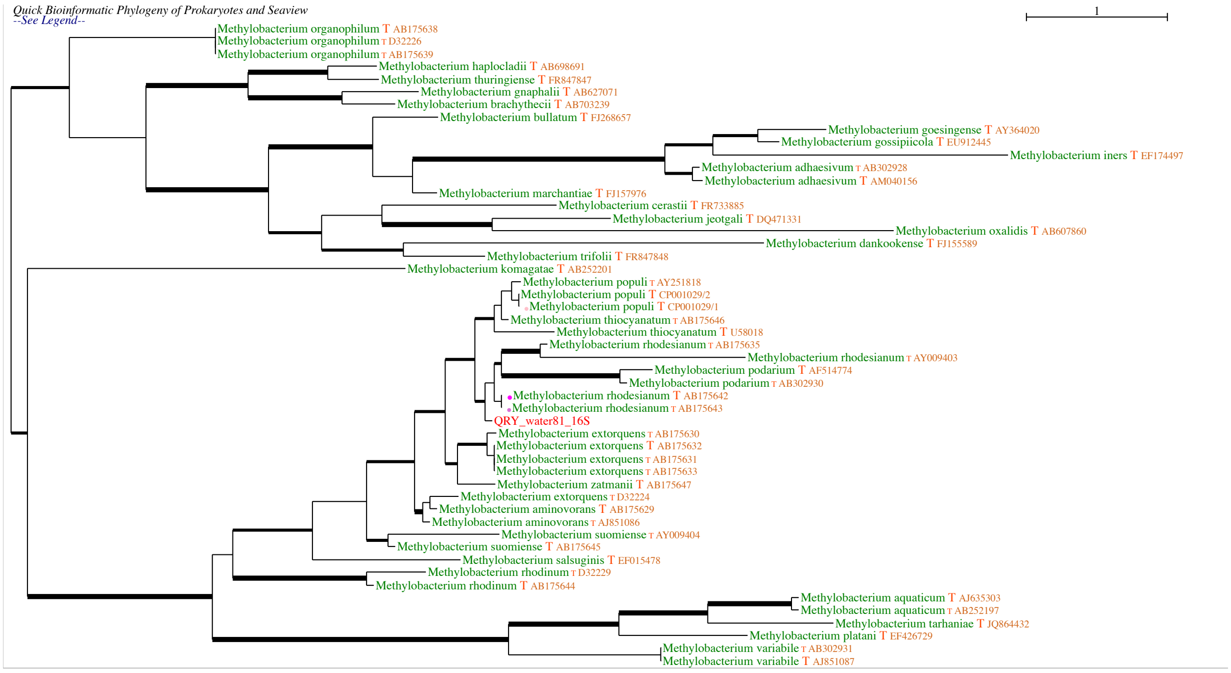

leBIBI QBPP Quick Bioinformatic Phylogeny of Prokaryotes | in short | How-to | Cite | Tests sets | Databases | Contact us | LBBE | prabi

lebibi Quick BioInformatic Phylogeny of Prokaryotes

Summary of the methods and database used during this session

Analysis Extraction of similar sequences for ingroup sequences: Query
There are 44 sequences in your batch
BLAST using the procaryota_SSU-rDNA-16S_TS-stringent database. Trying to extract 50 sequences with expect lower than 0.1
ALIGNMENT using mafft Options : speed oriented
BMGE use for alignment optimization Options :defaults parameters
TREE : phylogenetic tree using fasttree
Evolution model GTR -gamma

RESULTS

Results

Query data

Database: **procaryota_SSU-rDNA-16S_TS-stringent**
Note: The use of the stringent database (all the GenBank sequences for a given species) may lead to errors due to the low quality identification of some sequences, a careful analysis is necessary.
Original name of the query (from fasta) : **QRY_water82_16S.** leBiBI ref : pqpIvwPtL

Sequence composition

Length of Query sequence is : 1268
Nucleotide Composition 312 A - 243 T - 303 C - 410 G - **0 N 0.0 % Gaps % =0.0**
GC%=0.56

Quality of the BLAST analysis

The number of Blast hits is **OK**
All is OK, the best BLAST hit length (**1266**) is =99.8% of the query length

Biodiversity level

All extracted sequences appear to belong to Bacteria and their lowest shared taxon is: Bacteria-Actinobacteria-Actinobacteridae-Actinomycetales-Micrococcineae-Microbacteriaceae-Microbacterium
CAUTION ! One genus only in the extracted sequences. No outgroup *may* be pertinent enough for a good phylogeny. You may need to increase the number of desired sequences or use a *more stringent database*.

Phylogenetic tree analysis

Proximal Cluster ['Microbacterium_lacticum T X77441']

ANALYSIS OF PATRISTIC DISTANCES
Microbacterium_lacticum T X77441 is the closest sequence based on patristic distances
On the basis of patristic Distances, the **closest** sequence is: Microbacterium_lacticum T X77441 AND is in the proximal cluster
No statistics available for patristic distances between members of the species of the closest sequence
Remark: the patristic distance to Microbacterium_lacticum T X77441 is in the 75th percentile of the Microbacterium genus inter-species patristic distances : the sequence may correspond to a member of this genus

---

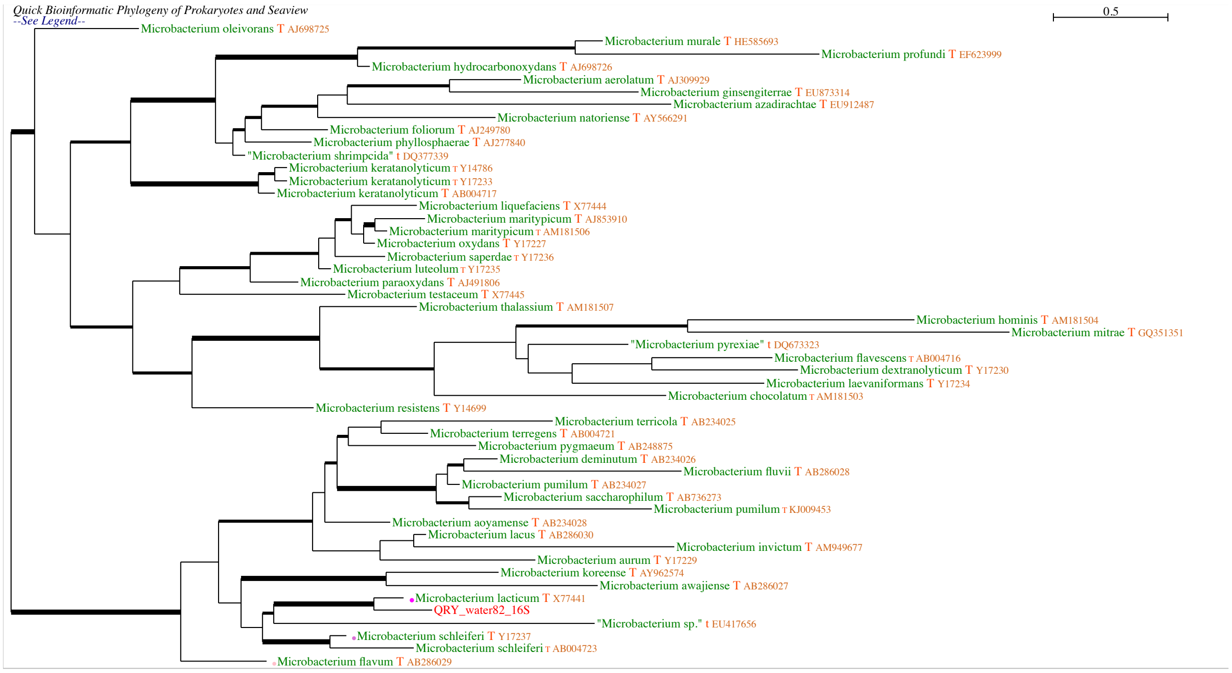

lebibi Quick BioInformatic Phylogeny of Prokaryotes

Summary of the methods and database used during this session

Analysis Extraction of similar sequences for ingroup sequences: Query
There are 44 sequences in your batch
BLAST using the procaryota_SSU-rDNA-16S_TS-stringent database. Trying to extract 50 sequences with expect lower than 0.1
ALIGNMENT using mafft Options : speed oriented
BMGE use for alignment optimization Options :defaults parameters
TREE : phylogenetic tree using fasttree
Evolution model GTR -gamma

RESULTS

Results

Query data

Database: **procaryota_SSU-rDNA-16S_TS-stringent**
Note: The use of the stringent database (all the GenBank sequences for a given species) may lead to errors due to the low quality identification of some sequences, a careful analysis is necessary.
Original name of the query (from fasta) : **QRY_water83_16S.** leBiBI ref : pqpIvwPtL

Sequence composition

Length of Query sequence is : 1268
Nucleotide Composition 311 A - 242 T - 304 C - 411 G - **0 N 0.0 %** **Gaps % =0.0**
GC%=0.56

Quality of the BLAST analysis

The number of Blast hits is **OK**
All is OK, the best BLAST hit length (**1266**) is =99.8% of the query length

Biodiversity level

All extracted sequences appear to belong to Bacteria and their lowest shared taxon is: Bacteria-Actinobacteria-Actinobacteridae-Actinomycetales-Micrococcineae-Microbacteriaceae-Microbacterium
CAUTION ! One genus only in the extracted sequences. No outgroup *may* be pertinent enough for a good phylogeny. You may need to increase the number of desired sequences or use a *more stringent database*.

Phylogenetic tree analysis

Proximal Cluster ['Microbacterium_lacticum T X77441']

ANALYSIS OF PATRISTIC DISTANCES
Microbacterium_lacticum T X77441 is the closest sequence based on patristic distances
On the basis of patristic Distances, the **closest** sequence is: Microbacterium_lacticum T X77441 AND is in the proximal cluster
No statistics available for patristic distances between members of the species of the closest sequence
Remark: the patristic distance to Microbacterium_lacticum T X77441 is in the 75th percentile of the Microbacterium genus inter-species patristic distances : the sequence may correspond to a member of this genus

---

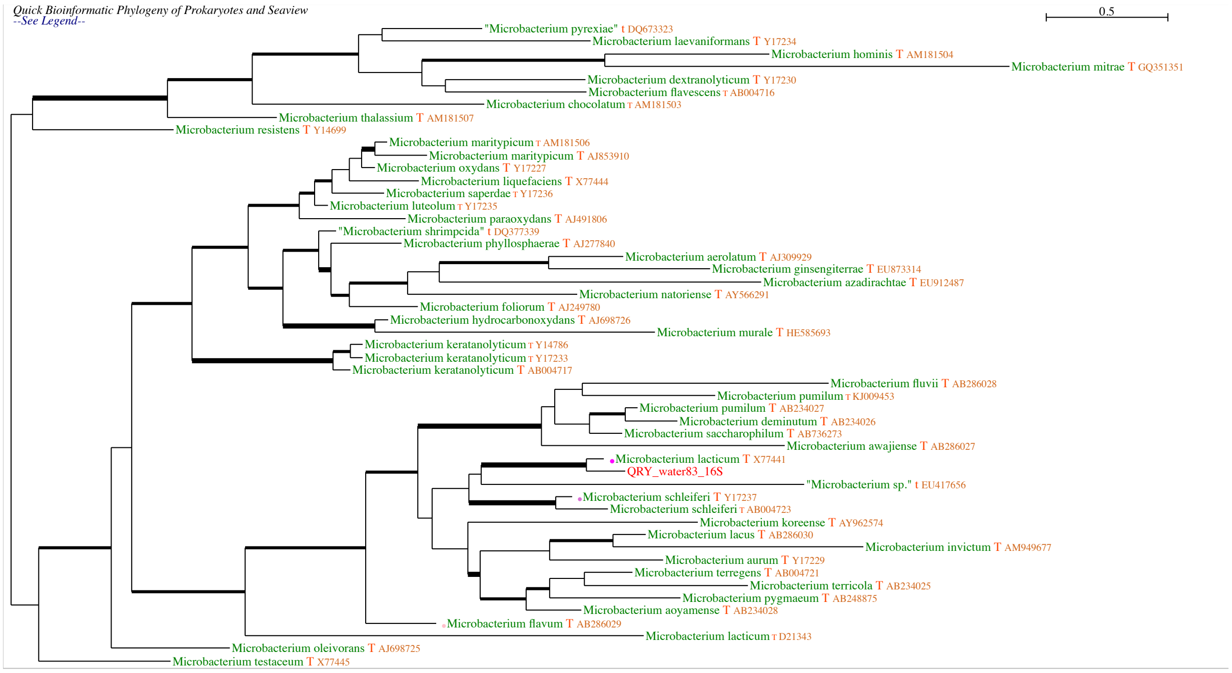

in short | How-to | Cite | Tests sets | Databases | Contact us

lebibi Quick BioInformatic Phylogeny of Prokaryotes

Summary of the methods and database used during this session

Analysis Extraction of similar sequences for ingroup sequences: Query
There are 44 sequences in your batch
BLAST using the procaryota_SSU-rDNA-16S_TS-stringent database. Trying to extract 50 sequences with expect lower than 0.1
ALIGNMENT using mafft Options : speed oriented
BMGE use for alignment optimization Options :defaults parameters
TREE : phylogenetic tree using fasttree
Evolution model GTR -gamma

RESULTS

Results

Query data

Database: **procaryota_SSU-rDNA-16S_TS-stringent**
Note: The use of the stringent database (all the GenBank sequences for a given species) may lead to errors due to the low quality identification of some sequences, a careful analysis is necessary.
Original name of the query (from fasta) : **QRY_water84_16S.** leBiBI ref : pqpIvwPtL

Sequence composition

Length of Query sequence is : 1231
Nucleotide Composition 290 A - 244 T - 296 C - 401 G - **0 N 0.0 % Gaps % =0.0**
GC%=0.56

Quality of the BLAST analysis

The number of Blast hits is **OK**
All is OK, the best BLAST hit length (**1229**) is =99.8% of the query length

Biodiversity level

All extracted sequences appear to belong to Bacteria and their lowest shared taxon is: Bacteria-Proteobacteria-Alphaproteobacteria-Rhizobiales-Methylobacteriaceae-Methylobacterium
CAUTION ! One genus only in the extracted sequences. No outgroup *may* be pertinent enough for a good phylogeny. You may need to increase the number of desired sequences or use a *more stringent database*.

Phylogenetic tree analysis

Proximal Cluster ['Methylobacterium_thiocyanatum T U58018', 'Methylobacterium_rhodesianum T AB175643', 'Methylobacterium_rhodesianum T AB175642']

ANALYSIS OF PATRISTIC DISTANCES
Methylobacterium_rhodesianum T AB175643 is the closest sequence based on patristic distances
On the basis of patristic Distances, the **closest** sequence is: Methylobacterium_rhodesianum T AB175643 AND is in the proximal cluster
No statistics available for patristic distances between members of the species of the closest sequence
Remark: the patristic distance to Methylobacterium_rhodesianum T AB175643 is in the 75th percentile of the Methylobacterium genus inter-species patristic distances : the sequence may correspond to a member of this genus
The closest sequence based on patristic distances (Methylobacterium_rhodesianum T AB175643) is NOT the FIRST BLAST hit.

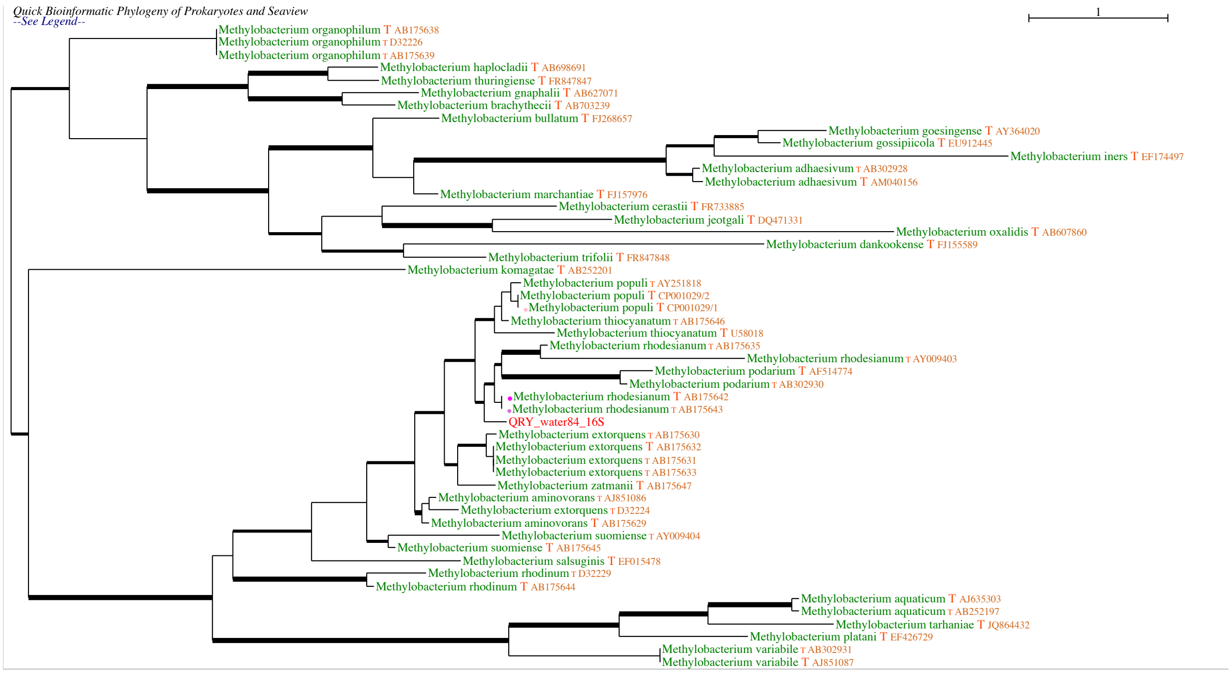

QBPP Quick Bioinformatic Phylogeny of Prokaryotes | in short | How-to | Cite | Tests sets | Databases | Contact us |

lebibi Quick BioInformatic Phylogeny of Prokaryotes

Summary of the methods and database used during this session

Analysis Extraction of similar sequences for ingroup sequences: Query
There are 44 sequences in your batch
BLAST using the procaryota_SSU-rDNA-16S_TS-stringent database. Trying to extract 50 sequences with expect lower than 0.1
ALIGNMENT using mafft Options : speed oriented
BMGE use for alignment optimization Options :defaults parameters
TREE : phylogenetic tree using fasttree
Evolution model GTR -gamma

RESULTS

Results

Query data

Database: **procaryota_SSU-rDNA-16S_TS-stringent**
Note: The use of the stringent database (all the GenBank sequences for a given species) may lead to errors due to the low quality identification of some sequences, a careful analysis is necessary.
Original name of the query (from fasta) : **QRY_water85_16S.** leBiBI ref : pqpIvwPtL

Sequence composition

Length of Query sequence is : 1231
Nucleotide Composition 291 A - 244 T - 296 C - 400 G - **0 N 0.0 % Gaps % =0.0**
GC%=0.56

Quality of the BLAST analysis

The number of Blast hits is **OK**
All is OK, the best BLAST hit length (**1229**) is =99.8% of the query length

Biodiversity level

All extracted sequences appear to belong to Bacteria and their lowest shared taxon is: Bacteria-Proteobacteria-Alphaproteobacteria-Rhizobiales-Methylobacteriaceae-Methylobacterium
CAUTION ! One genus only in the extracted sequences. No outgroup *may* be pertinent enough for a good phylogeny. You may need to increase the number of desired sequences or use a *more stringent database*.

Phylogenetic tree analysis

Proximal Cluster ['Methylobacterium_thiocyanatum T U58018', 'Methylobacterium_rhodesianum T AB175643', 'Methylobacterium_rhodesianum T AB175642']

ANALYSIS OF PATRISTIC DISTANCES
Methylobacterium_rhodesianum T AB175643 is the closest sequence based on patristic distances
On the basis of patristic Distances, the **closest** sequence is: Methylobacterium_rhodesianum T AB175643 AND is in the proximal cluster
No statistics available for patristic distances between members of the species of the closest sequence
Remark: the patristic distance to Methylobacterium_rhodesianum T AB175643 is in the 75th percentile of the Methylobacterium genus inter-species patristic distances : the sequence may correspond to a member of this genus
The closest sequence based on patristic distances (Methylobacterium_rhodesianum T AB175643) is NOT the FIRST BLAST hit.

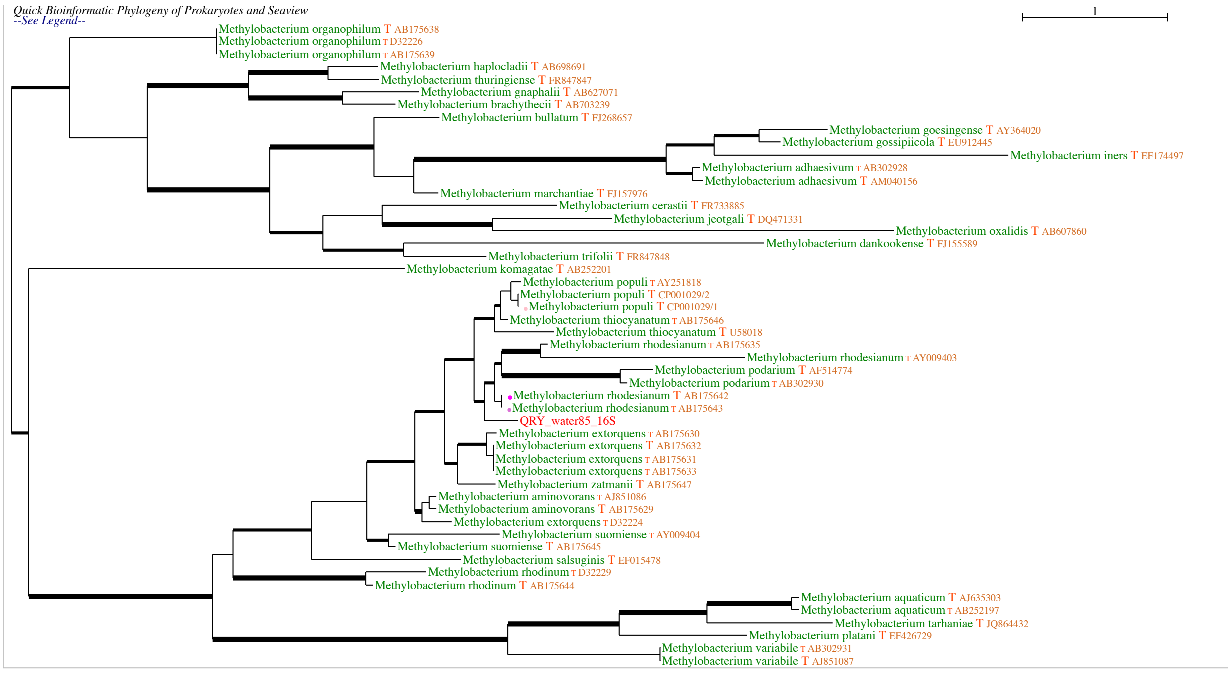

le BIBI QBPP Quick Bioinformatic Phylogeny of Prokaryotes | in short | How-to | Cite | Tests sets | Databases | Contact us | LBBE | prabi

lebibi Quick BioInformatic Phylogeny of Prokaryotes

Summary of the methods and database used during this session

Analysis Extraction of similar sequences for ingroup sequences: Query
There are 44 sequences in your batch
BLAST using the procaryota_SSU-rDNA-16S_TS-stringent database. Trying to extract 50 sequences with expect lower than 0.1
ALIGNMENT using mafft Options : speed oriented
BMGE use for alignment optimization Options :defaults parameters
TREE : phylogenetic tree using fasttree
Evolution model GTR -gamma

RESULTS

Results

Query data

Database: **procaryota_SSU-rDNA-16S_TS-stringent**
Note: The use of the stringent database (all the GenBank sequences for a given species) may lead to errors due to the low quality identification of some sequences, a careful analysis is necessary.
Original name of the query (from fasta) : **QRY_water38_16S.** leBiBI ref : pqpIvwPtL

Sequence composition

Length of Query sequence is : 1270
Nucleotide Composition 314 A - 243 T - 303 C - 410 G - **0 N 0.0 % Gaps % =0.0**
GC%=0.56

Quality of the BLAST analysis

The number of Blast hits is **OK**
All is OK, the best BLAST hit length (**1270**) is =100.% of the query length

Biodiversity level

All extracted sequences appear to belong to Bacteria and their lowest shared taxon is: Bacteria-Actinobacteria-Actinobacteridae-Actinomycetales-Micrococcineae-Microbacteriaceae-Microbacterium
CAUTION ! One genus only in the extracted sequences. No outgroup *may* be pertinent enough for a good phylogeny. You may need to increase the number of desired sequences or use a *more stringent database*.

Phylogenetic tree analysis

Proximal Cluster ['Microbacterium_flavum T AB286029', 'Microbacterium_lacticum T X77441']

ANALYSIS OF PATRISTIC DISTANCES
Microbacterium_lacticum T X77441 is the closest sequence based on patristic distances
On the basis of patristic Distances, the **closest** sequence is: Microbacterium_lacticum T X77441 AND is in the proximal cluster
No statistics available for patristic distances between members of the species of the closest sequence
Remark: the patristic distance to Microbacterium_lacticum T X77441 is in the 75th percentile of the Microbacterium genus inter-species patristic distances : the sequence may correspond to a member of this genus

---

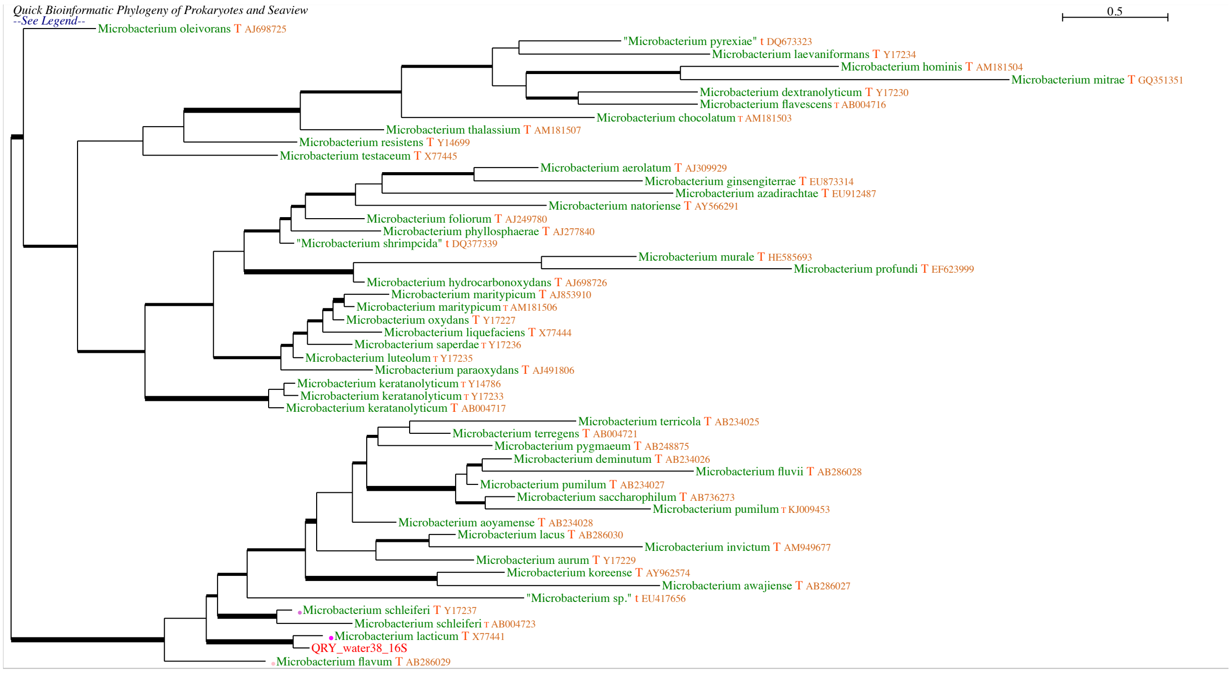

in short | How-to | Cite | Tests sets | Databases | Contact us

lebibi Quick BioInformatic Phylogeny of Prokaryotes

Summary of the methods and database used during this session

Analysis Extraction of similar sequences for ingroup sequences: Query
There are 44 sequences in your batch
BLAST using the procaryota_SSU-rDNA-16S_TS-stringent database. Trying to extract 50 sequences with expect lower than 0.1
ALIGNMENT using mafft Options : speed oriented
BMGE use for alignment optimization Options :defaults parameters
TREE : phylogenetic tree using fasttree
Evolution model GTR -gamma

RESULTS

Results

Query data

Database: **procaryota_SSU-rDNA-16S_TS-stringent**
Note: The use of the stringent database (all the GenBank sequences for a given species) may lead to errors due to the low quality identification of some sequences, a careful analysis is necessary.
Original name of the query (from fasta) : **QRY_water39_16S.** leBiBI ref : pqpIvwPtL

Sequence composition

Length of Query sequence is : 1278
Nucleotide Composition 318 A - 246 T - 303 C - 411 G - **0 N 0.0 %** **Gaps % =0.0**
GC%=0.55

Quality of the BLAST analysis

The number of Blast hits is **OK**
All is OK, the best BLAST hit length (**1273**) is =99.6% of the query length

Biodiversity level

All extracted sequences appear to belong to Bacteria and their lowest shared taxon is: Bacteria-Actinobacteria-Actinobacteridae-Actinomycetales-Micrococcineae-Microbacteriaceae
More than one genus in the extracted sequences. An outgroup will be present in the tree.

Phylogenetic tree analysis

Proximal Cluster ['Microbacterium_hatanonis T AB274908', 'Microbacterium_aurum T Y17229']

ANALYSIS OF PATRISTIC DISTANCES
Microbacterium_aurum T Y17229 is the closest sequence based on patristic distances
On the basis of patristic Distances, the **closest** sequence is: Microbacterium_aurum T Y17229 AND is in the proximal cluster
No statistics available for patristic distances between members of the species of the closest sequence
Remark: the patristic distance to Microbacterium_aurum T Y17229 is in the 75th percentile of the Microbacterium genus inter-species patristic distances : the sequence may correspond to a member of this genus

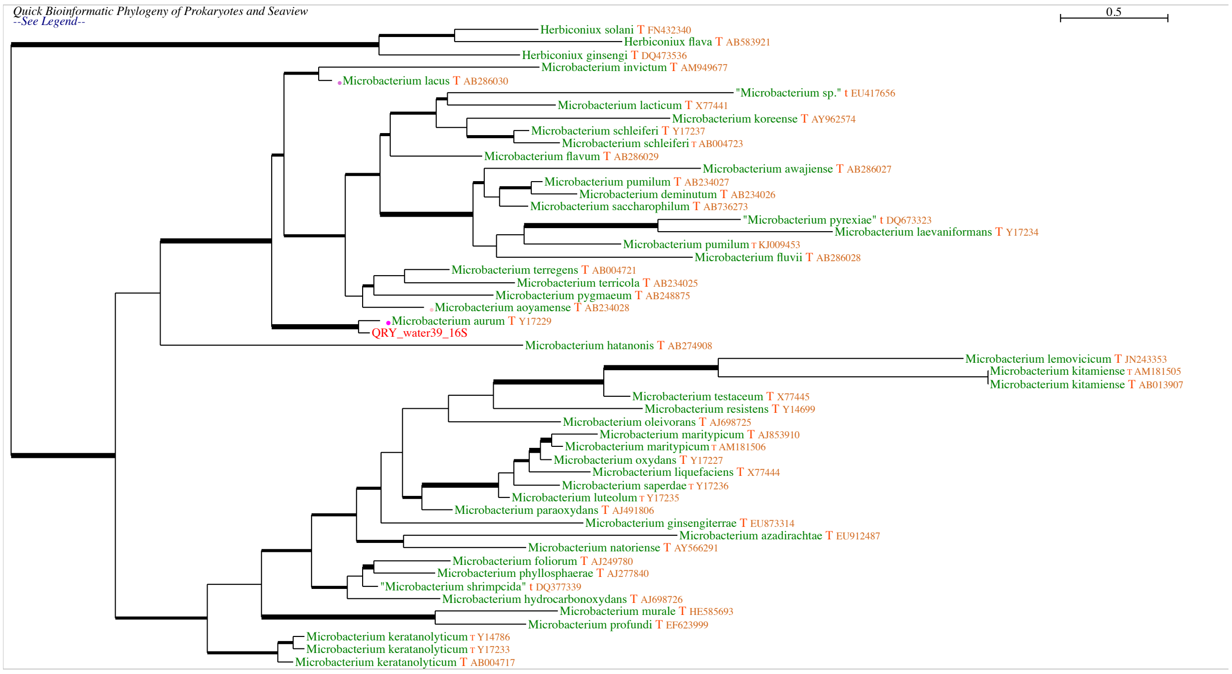

leBIBI QBPP Quick Bioinformatic Phylogeny of Prokaryotes | in short | How-to | Cite | Tests sets | Databases | Contact us | LBBE | prabi

lebibi Quick BioInformatic Phylogeny of Prokaryotes

Summary of the methods and database used during this session

Analysis Extraction of similar sequences for ingroup sequences: Query
There are 44 sequences in your batch
BLAST using the procaryota_SSU-rDNA-16S_TS-stringent database. Trying to extract 50 sequences with expect lower than 0.1
ALIGNMENT using mafft Options : speed oriented
BMGE use for alignment optimization Options :defaults parameters
TREE : phylogenetic tree using fasttree
Evolution model GTR -gamma

RESULTS

Results

Query data

Database: **procaryota_SSU-rDNA-16S_TS-stringent**
Note: The use of the stringent database (all the GenBank sequences for a given species) may lead to errors due to the low quality identification of some sequences, a careful analysis is necessary.
Original name of the query (from fasta) : **QRY_water40_16S.** leBiBI ref : pqpIvwPtL

Sequence composition

Length of Query sequence is : 1267
Nucleotide Composition 288 A - 258 T - 292 C - 428 G - **0 N 0.0 % Gaps % =1.0**
GC%=0.56

Quality of the BLAST analysis

The number of Blast hits is **OK**
All is OK, the best BLAST hit length (**1260**) is =99.4% of the query length

Biodiversity level

All extracted sequences appear to belong to Bacteria and their lowest shared taxon is: Bacteria-Actinobacteria-Actinobacteridae-Actinomycetales-Micrococcineae-Micrococcaceae
More than one genus in the extracted sequences. An outgroup will be present in the tree.

Phylogenetic tree analysis

Proximal Cluster ['Micrococcus_luteus T AM992194', 'Micrococcus_aloeverae T KF524364']

ANALYSIS OF PATRISTIC DISTANCES
Micrococcus_aloeverae T KF524364 is the closest sequence based on patristic distances
On the basis of patristic Distances, the **closest** sequence is: Micrococcus_aloeverae T KF524364 AND is in the proximal cluster
No statistics available for patristic distances between members of the species of the closest sequence
Remark: the patristic distance to Micrococcus_aloeverae T KF524364 is in the 75th percentile of the Micrococcus genus inter-species patristic distances : the sequence may correspond to a member of this genus
The closest sequence based on patristic distances (Micrococcus_aloeverae T KF524364) is NOT the FIRST BLAST hit.

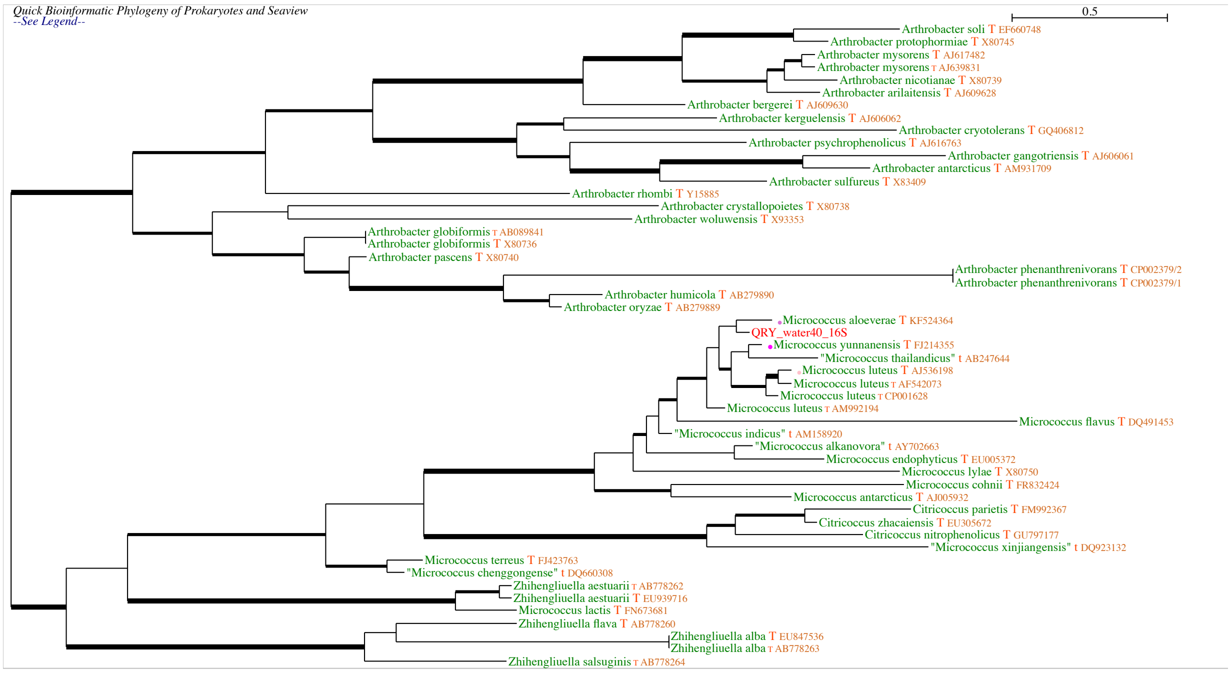

in short | How-to | Cite | Tests sets | Databases | Contact us

lebibi Quick BioInformatic Phylogeny of Prokaryotes

Summary of the methods and database used during this session

Analysis Extraction of similar sequences for ingroup sequences: Query
There are 44 sequences in your batch
BLAST using the procaryota_SSU-rDNA-16S_TS-stringent database. Trying to extract 50 sequences with expect lower than 0.1
ALIGNMENT using mafft Options : speed oriented
BMGE use for alignment optimization Options :defaults parameters
TREE : phylogenetic tree using fasttree
Evolution model GTR -gamma

RESULTS

Results

Query data

Database: **procaryota_SSU-rDNA-16S_TS-stringent**
Note: The use of the stringent database (all the GenBank sequences for a given species) may lead to errors due to the low quality identification of some sequences, a careful analysis is necessary.
Original name of the query (from fasta) : **QRY_water41_16S.** leBiBI ref : pqpIvwPtL

Sequence composition

Length of Query sequence is : 1278
Nucleotide Composition 318 A - 246 T - 303 C - 411 G - **0 N 0.0 %** **Gaps % =0.0**
GC%=0.55

Quality of the BLAST analysis

The number of Blast hits is **OK**
All is OK, the best BLAST hit length (**1273**) is =99.6% of the query length

Biodiversity level

All extracted sequences appear to belong to Bacteria and their lowest shared taxon is: Bacteria-Actinobacteria-Actinobacteridae-Actinomycetales-Micrococcineae-Microbacteriaceae
More than one genus in the extracted sequences. An outgroup will be present in the tree.

Phylogenetic tree analysis

Proximal Cluster ['Microbacterium_hatanonis T AB274908', 'Microbacterium_aurum T Y17229']

ANALYSIS OF PATRISTIC DISTANCES
Microbacterium_aurum T Y17229 is the closest sequence based on patristic distances
On the basis of patristic Distances, the **closest** sequence is: Microbacterium_aurum T Y17229 AND is in the proximal cluster
No statistics available for patristic distances between members of the species of the closest sequence
Remark: the patristic distance to Microbacterium_aurum T Y17229 is in the 75th percentile of the Microbacterium genus inter-species patristic distances : the sequence may correspond to a member of this genus

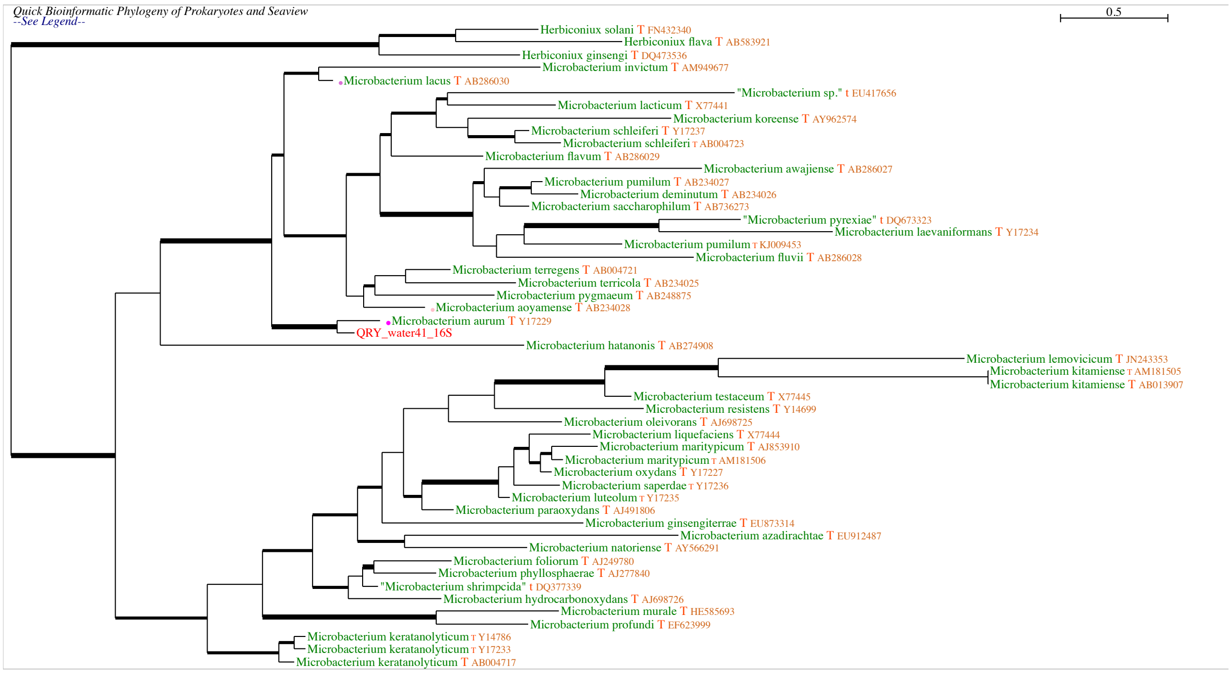

in short | How-to | Cite | Tests sets | Databases | Contact us

lebibi Quick BioInformatic Phylogeny of Prokaryotes

Summary of the methods and database used during this session

Analysis Extraction of similar sequences for ingroup sequences: Query
There are 44 sequences in your batch
BLAST using the procaryota_SSU-rDNA-16S_TS-stringent database. Trying to extract 50 sequences with expect lower than 0.1
ALIGNMENT using mafft Options : speed oriented
BMGE use for alignment optimization Options :defaults parameters
TREE : phylogenetic tree using fasttree
Evolution model GTR -gamma

RESULTS

Results

Query data

Database: **procaryota_SSU-rDNA-16S_TS-stringent**
Note: The use of the stringent database (all the GenBank sequences for a given species) may lead to errors due to the low quality identification of some sequences, a careful analysis is necessary.
Original name of the query (from fasta) : **QRY_water42_16S.** leBiBI ref : pqpIvwPtL

Sequence composition

Length of Query sequence is : 1280
Nucleotide Composition 316 A - 244 T - 307 C - 413 G - **0 N 0.0 %** **Gaps % =0.0**
GC%=0.56

Quality of the BLAST analysis

The number of Blast hits is **OK**
All is OK, the best BLAST hit length (**1280**) is =100.% of the query length

Biodiversity level

All extracted sequences appear to belong to Bacteria and their lowest shared taxon is: Bacteria-Actinobacteria-Actinobacteridae-Actinomycetales-Micrococcineae-Microbacteriaceae-Microbacterium
CAUTION ! One genus only in the extracted sequences. No outgroup *may* be pertinent enough for a good phylogeny. You may need to increase the number of desired sequences or use a *more stringent database*.

Phylogenetic tree analysis

Proximal Cluster ['Microbacterium_lacticum T X77441']

ANALYSIS OF PATRISTIC DISTANCES
Microbacterium_lacticum T X77441 is the closest sequence based on patristic distances
On the basis of patristic Distances, the **closest** sequence is: Microbacterium_lacticum T X77441 AND is in the proximal cluster
No statistics available for patristic distances between members of the species of the closest sequence
Remark: the patristic distance to Microbacterium_lacticum T X77441 is in the 75th percentile of the Microbacterium genus inter-species patristic distances : the sequence may correspond to a member of this genus

---

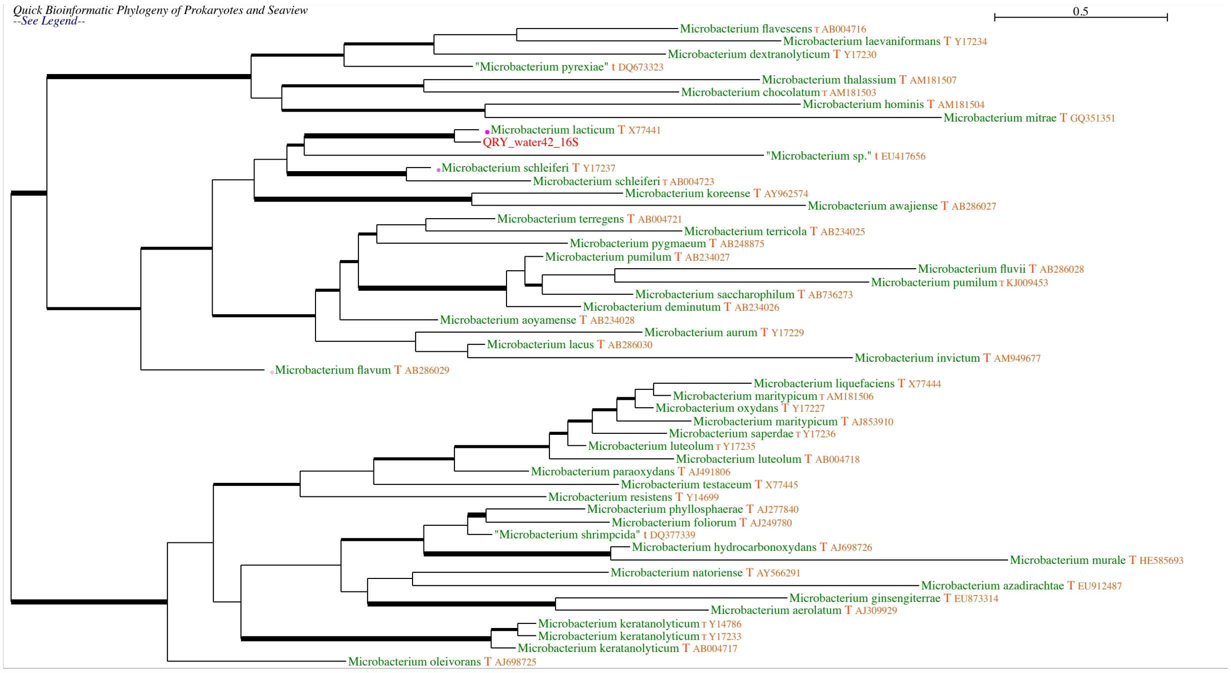

lebibi Quick BioInformatic Phylogeny of Prokaryotes

Summary of the methods and database used during this session

Analysis Extraction of similar sequences for ingroup sequences: Query
There are 44 sequences in your batch
BLAST using the procaryota_SSU-rDNA-16S_TS-stringent database. Trying to extract 50 sequences with expect lower than 0.1
ALIGNMENT using mafft Options : speed oriented
BMGE use for alignment optimization Options :defaults parameters
TREE : phylogenetic tree using fasttree
Evolution model GTR -gamma

RESULTS

Results

Query data

Database: **procaryota_SSU-rDNA-16S_TS-stringent**
Note: The use of the stringent database (all the GenBank sequences for a given species) may lead to errors due to the low quality identification of some sequences, a careful analysis is necessary.
Original name of the query (from fasta) : **QRY_water43_16S.** leBiBI ref : pqpIvwPtL

Sequence composition

Length of Query sequence is : 1269
Nucleotide Composition 312 A - 242 T - 304 C - 411 G - **0 N 0.0 % Gaps % =0.0**
GC%=0.56

Quality of the BLAST analysis

The number of Blast hits is **OK**
All is OK, the best BLAST hit length (**1269**) is =100.% of the query length

Biodiversity level

All extracted sequences appear to belong to Bacteria and their lowest shared taxon is: Bacteria-Actinobacteria-Actinobacteridae-Actinomycetales-Micrococcineae-Microbacteriaceae-Microbacterium
CAUTION ! One genus only in the extracted sequences. No outgroup *may* be pertinent enough for a good phylogeny. You may need to increase the number of desired sequences or use a *more stringent database*.

Phylogenetic tree analysis

Proximal Cluster ['Microbacterium_lacticum T X77441']

ANALYSIS OF PATRISTIC DISTANCES
Microbacterium_lacticum T X77441 is the closest sequence based on patristic distances
On the basis of patristic Distances, the **closest** sequence is: Microbacterium_lacticum T X77441 AND is in the proximal cluster
No statistics available for patristic distances between members of the species of the closest sequence
Remark: the patristic distance to Microbacterium_lacticum T X77441 is in the 75th percentile of the Microbacterium genus inter-species patristic distances : the sequence may correspond to a member of this genus

---

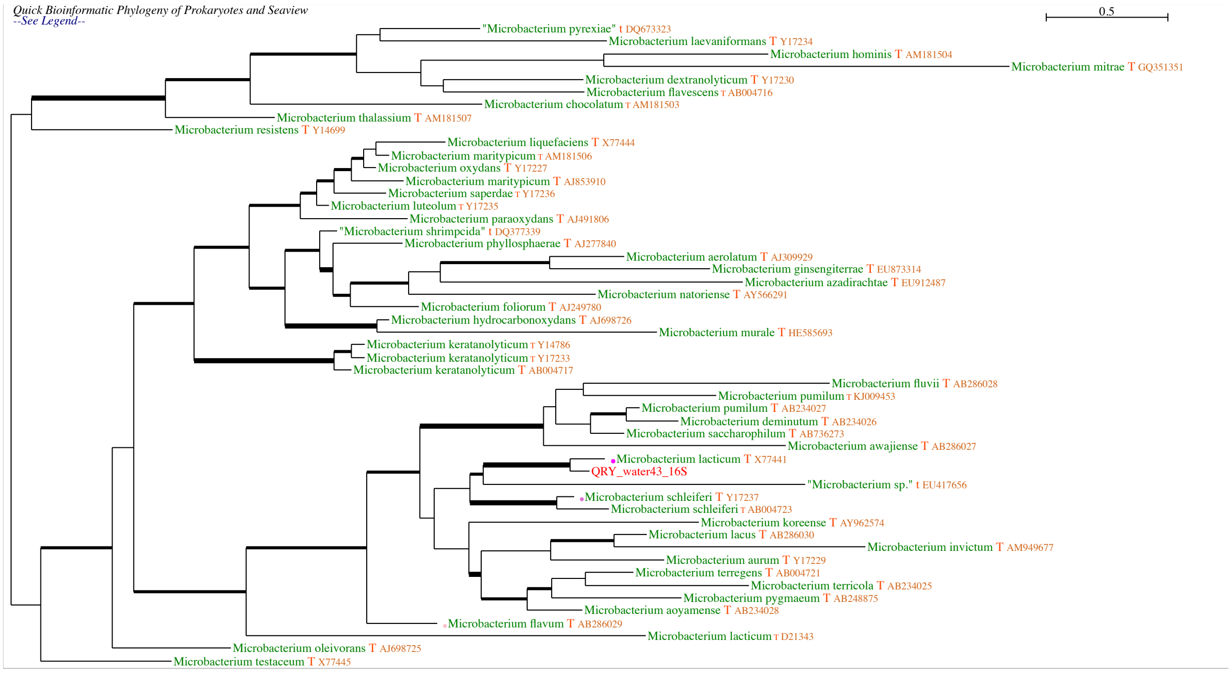

in short | How-to | Cite | Tests sets | Databases | Contact us

lebibi Quick BioInformatic Phylogeny of Prokaryotes

Summary of the methods and database used during this session

Analysis Extraction of similar sequences for ingroup sequences: Query
There are 44 sequences in your batch
BLAST using the procaryota_SSU-rDNA-16S_TS-stringent database. Trying to extract 50 sequences with expect lower than 0.1
ALIGNMENT using mafft Options : speed oriented
BMGE use for alignment optimization Options :defaults parameters
TREE : phylogenetic tree using fasttree
Evolution model GTR -gamma

RESULTS
